# Design, Synthesis, and Biological Activity of Novel Ornithine Decarboxylase (ODC) Inhibitors

**DOI:** 10.1021/acs.jmedchem.4c03120

**Published:** 2025-03-04

**Authors:** Chad R. Schultz, Bilal Aleiwi, X. Edward Zhou, Kelly Suino-Powell, Karsten Melcher, Nuno M. S. Almeida, Angela K. Wilson, Edmund L. Ellsworth, André S. Bachmann

**Affiliations:** † Department of Pediatrics and Human Development, College of Human Medicine, Michigan State University, Grand Rapids, Michigan 49503, United States; ‡ International Center for Polyamine Disorders, Grand Rapids, Michigan 49503, United States; § Department of Pharmacology and Toxicology, College of Human Medicine, East Lansing, Michigan 48824, United States; ∥ Department of Structural Biology, Van Andel Institute, Grand Rapids, Michigan 49503, United States; ⊥ Department of Chemistry, College of Natural Science, Michigan State University, East Lansing, Michigan 48824, United States

## Abstract

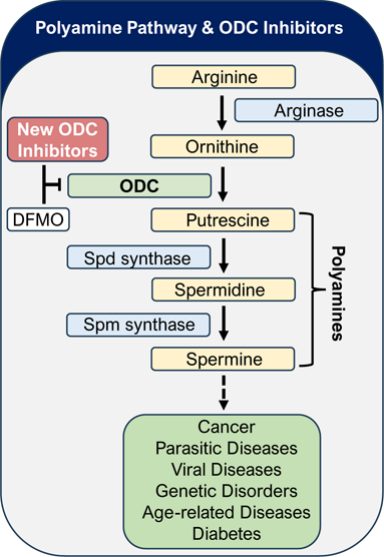

We here describe the design, synthesis, and biological activity of novel ornithine decarboxylase (ODC) inhibitors that show significantly higher potency *in vitro* than α-difluoromethylornithine (DFMO), a U.S. Food and Drug Administration (FDA) approved drug. We report two X-ray structures of ODC complexed with new ODC inhibitors, computational docking, molecular dynamics, and binding free energy calculations to validate the experimental models. The X-ray structures reveal that covalent adducts with pyridoxal phosphate (PLP) are formed in the active site of the human ODC enzyme, as verified by their preparation and enzymatic testing. Finally, we verified that the cellular activity of endogenous ODC was inhibited, and polyamine levels were reduced. Given that ODC is a clinically validated target, combined with the fact that DFMO is currently the only ODC inhibitor in clinical use for several indications, the further development of more potent ODC inhibitors with superior activity and physical properties is warranted.

## Introduction

Ornithine decarboxylase (ODC) is a pyridoxal phosphate (PLP)-dependent enzyme that catalyzes the conversion of ornithine to putrescine, a rate-limiting first step for the tightly regulated biosynthesis of polyamines (putrescine, spermidine, spermine).^1−3^ At the molecular level, positively charged polyamines interact with negatively charged RNA, DNA, and phospholipids, thereby directly contributing to chromatin biology.^4,5^ Polyamines regulate physiological processes during embryogenesis, spermatogenesis, and organogenesis. In cancer, they play a key role in cell cycle progression, proliferation, differentiation, apoptosis, tumorigenesis, and immune system function.^6−9^ ODC is a transcriptional target of the myelocytomatosis (MYC) protein, which belongs to a family of regulator genes and proto-oncogenes that code for transcription factors.^10,11^ MYC is frequently dysregulated/amplified in tumors including neuroblastoma,^12^ and pharmacological inhibition of ODC with α-difluoromethylornithine (**DFMO**, also known as eflornithine, ornidyl, or iwilfin) induces antiproliferative activity.^10−14^ Synthesized in 1978,^15^**DFMO** was historically developed as an anticancer agent (oral formulation)^16,17^ and later repurposed and U.S. Food and Drug Administration (FDA)-approved for the treatment of West African sleeping sickness (trypanosomiasis) (intravenous formulation)^18−22^ and hirsutism in women (topical formulation).^23,24^**DFMO** has received renewed attention for various cancer chemopreventive and chemotherapeutic applications in colorectal cancer, prostate cancer, and neuroblastoma^13,16,17,25−31^ and most recently for the treatment of a rare genetic disease called Bachmann-Bupp syndrome^32,33^ and type 1 diabetes.^34^ Importantly, on December 13, 2023, **DFMO** (Iwilfin) was approved by the FDA for the first time in its 45-year history as an orally dosed anticancer drug for patients with neuroblastoma. Since the approval of **DFMO**, a diversity of other inhibitors has been reported, yet none have been advanced to the clinic for evaluation.^35−40^

**Figure 1:**
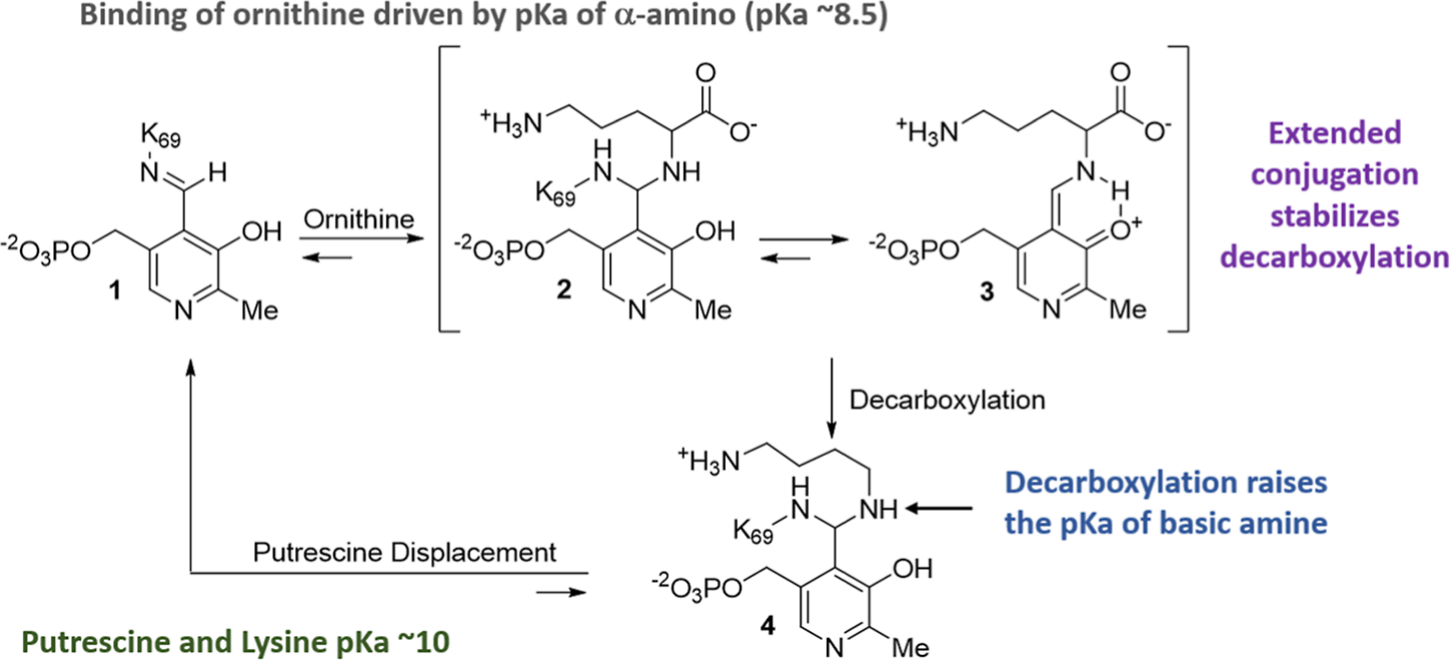
Catalytic cycle of the conversion of ornithine to putrescine by ornithine decarboxylase (ODC).

The ODC enzyme is a homodimer, with the dimer interface forming its catalytic site from residues from both monomers. The apo X-ray structure of human ODC (1D7K), detailing the features of the protein and binding domain is published.^41^ The binding pocket, typically occupied by the natural substrate ornithine, can be described as having two terminal polar regions, one binding the cofactor PLP in a reversible fashion. The catalytic cycle (Figure 1), driven by p*K*_a_ differences in water, impacts imine formation with the natural substrate ornithine and the product putrescine’s release from the active site. In the absence of ornithine, ODC covalently binds its cofactor PLP through the formation of an imine (**1**) from PLP and the ε-amino (p*K*_a_ ∼ 10.5) group of ODC Lys,^69^ demonstrated in its X-ray structure (1D7K). The α-amino group on ornithine (p*K*_a_ ∼ 8.7)^42^ competitively displaces the ε-amino group (**2**) of Lys^69^ to form an imine (**3**) with PLP. The lipophilic carbon train of ornithine stretches though a lipophilic channel and the terminal basic ε-amine likely makes binding interactions with Asp^332^ and/or Asp^361^. The formed ornithine-PLP imine (**3**) then decarboxylates to form a putrescine-PLP adduct (**4**), likely stabilized by Cys^360^. The decarboxylation increases the p*K*_a_ (∼10.5)^42^ of the resulting putrescine nitrogen allowing its displacement by Lys^69^ through transamination, thereby closing the catalytic cycle (Figure 1). Although putrescine is not an inhibitor of ODC, even at high (>500 μM) concentrations, an X-ray structure of putrescine bound ODC (*Trypanosoma brucei*) (1F3T) is published.^43^ It shows that the imine is stabilized by Cys^360^, rather than the formation of the aminal (**4**) formed with Lys^69^ and plays a role in the catalytic cycle.

**Figure 2:**
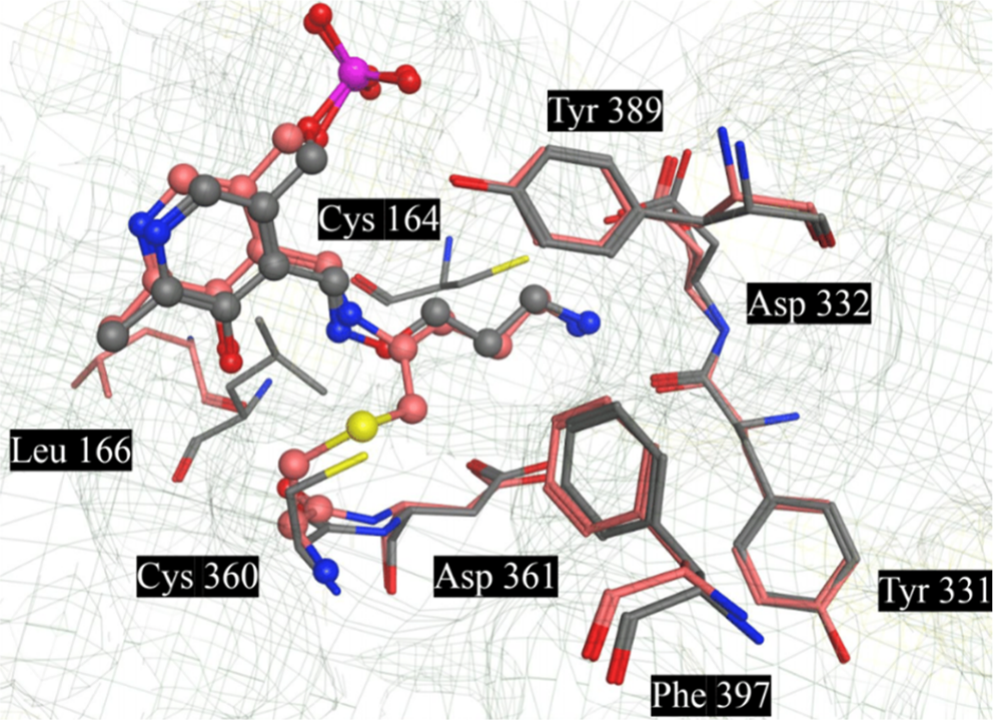
X-ray structures of α-difluoromethylornithine (**DFMO**) (2TOD) bound to ornithine decarboxylase (ODC) (*T. brucei*, red–orange) overlapped with 1-amino-oxy-3-aminopropane (**APA**) (7S3F) (human, Gray).

**DFMO** is water-soluble and demonstrates significant safety but has low ODC affinity and rapid clearance (Table 1)^44^ It requires an extremely high dosing paradigm to achieve the desired exposures needed for efficacy. Mechanistically, **DFMO** (α-NH_2_ p*K*_a_ ∼ 8.0) is accepted as a substrate by ODC, binding competitively with ornithine (α-NH2 p*K*_a_ ∼ 8.0), the binding favored in part due to its lower p*K*_a_. In the catalytic cycle, it forms an irreversible decarboxylated adduct, its irreversibility coming from the displacement by Cys^360^ of one of the fluorines of **DFMO** to deactivate the enzyme. An X-ray structure of the irreversible adduct bound to *T. brucei* ODC was published (2TOD, Figure 2).^45^ Key binding features include the terminal amine, binding through active site water molecules to Asp^332^ and Asp^361^, while the alkyl tether makes a key interaction with Tyr^389^, which is part of a lipophilic pocket formed between the two monomers.

**Table 1:**
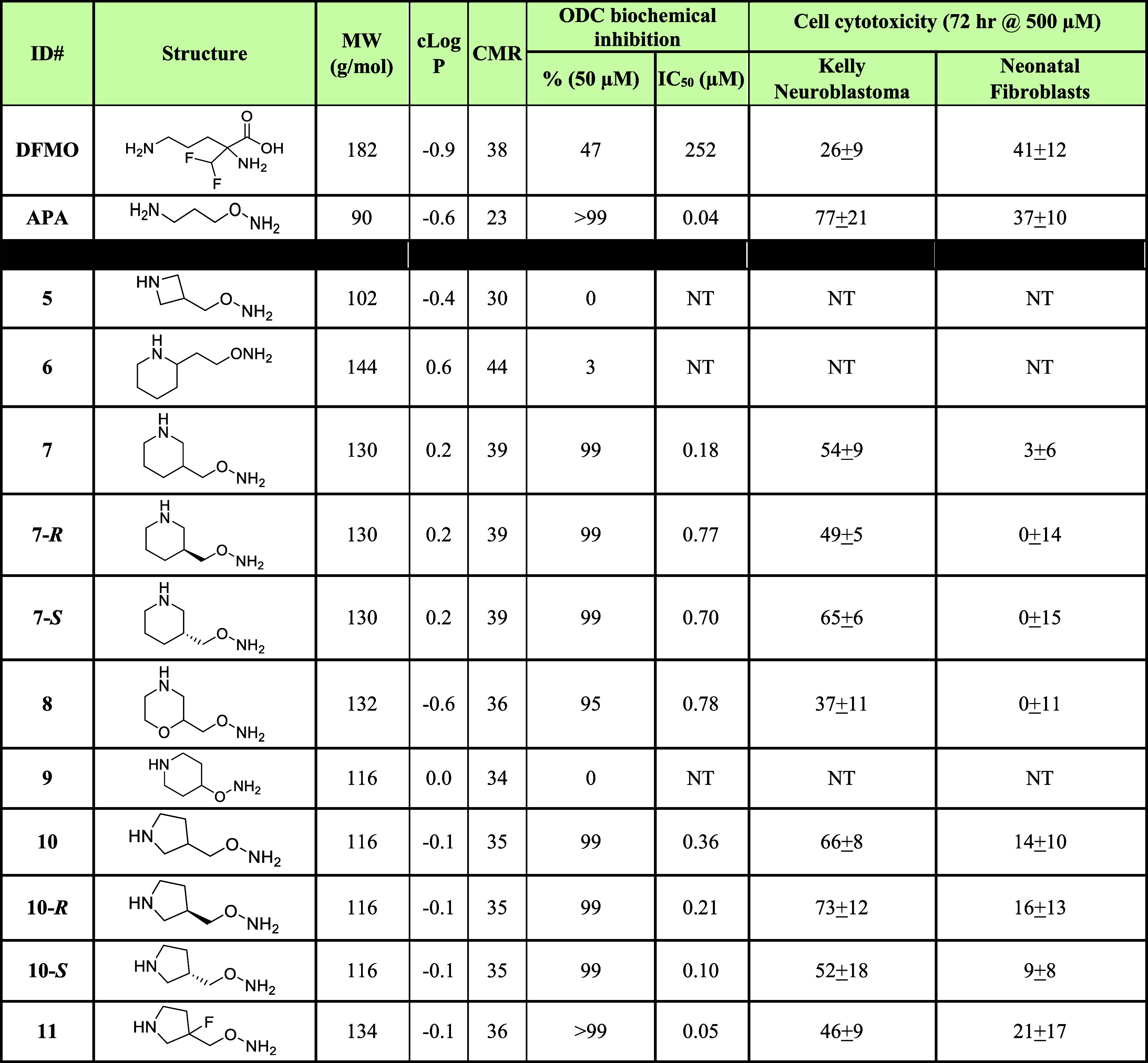
Structure-Activity Relationships (SAR) Studies Exploring Cyclic Analogs of 1-Amino-oxy-3-aminopropane (**APA**)^a^

a NT = not tested; CMR = calculated molar refractivity; cLogP = calculated logarithm of its partition coefficient between *n*-octanol and water; MW = molecular weight; IC_50_ = inhibitory concentration at 50%; APA = 1-amino-oxy-3-aminopropane; DFMO = α-difluoromethylornithine; ODC = ornithine decarboxylase.

The molecule 1-amino-oxy-3-aminopropane (**APA**) was first described in 1985 as a potent competitive inhibitor (Ki = 3.2 nM) of homogeneous mouse kidney ODC, with weaker activities as an irreversible inhibitor (Ki = 50 μM) of homogeneous liver adenosylmethionine decarboxylase and a competitive inhibitor (Ki = 2.3 μM) of homogeneous bovine brain spermidine synthase.^46^**APA** was found to inhibit cell proliferation through the inhibition of ODC and suppression of polyamines in leukemia L1210 cells^47^ and in Ehrlich ascites-tumor cells.^48^ This improved ODC inhibitor **APA** (p*K*_a_ –ONH_2_ ∼ 4.0, Table 2), and related near neighbor acyclic substituted analogs, demonstrated significantly improved ODC inhibition, with an IC_50_ against human ODC in the low nM range, i.e., a potency that is 10,000 times higher than that of DFMO^37,44,49−51^ and exhibiting low nM activity. The hydroxyl amine of these derivatives has a lower p*K*_a_ (∼5.9).^52^ At pH 7.2 it would be un-ionized and would be competitive with the binding of the natural substrate ornithine. However, the reported X-ray structure of **APA**(44) bound to ODC, unlike that seen with the natural substrate, does not make the predicted oxime, but instead makes a simple hydrogen bonding interaction with PLP aldehyde.

**Table 2:**
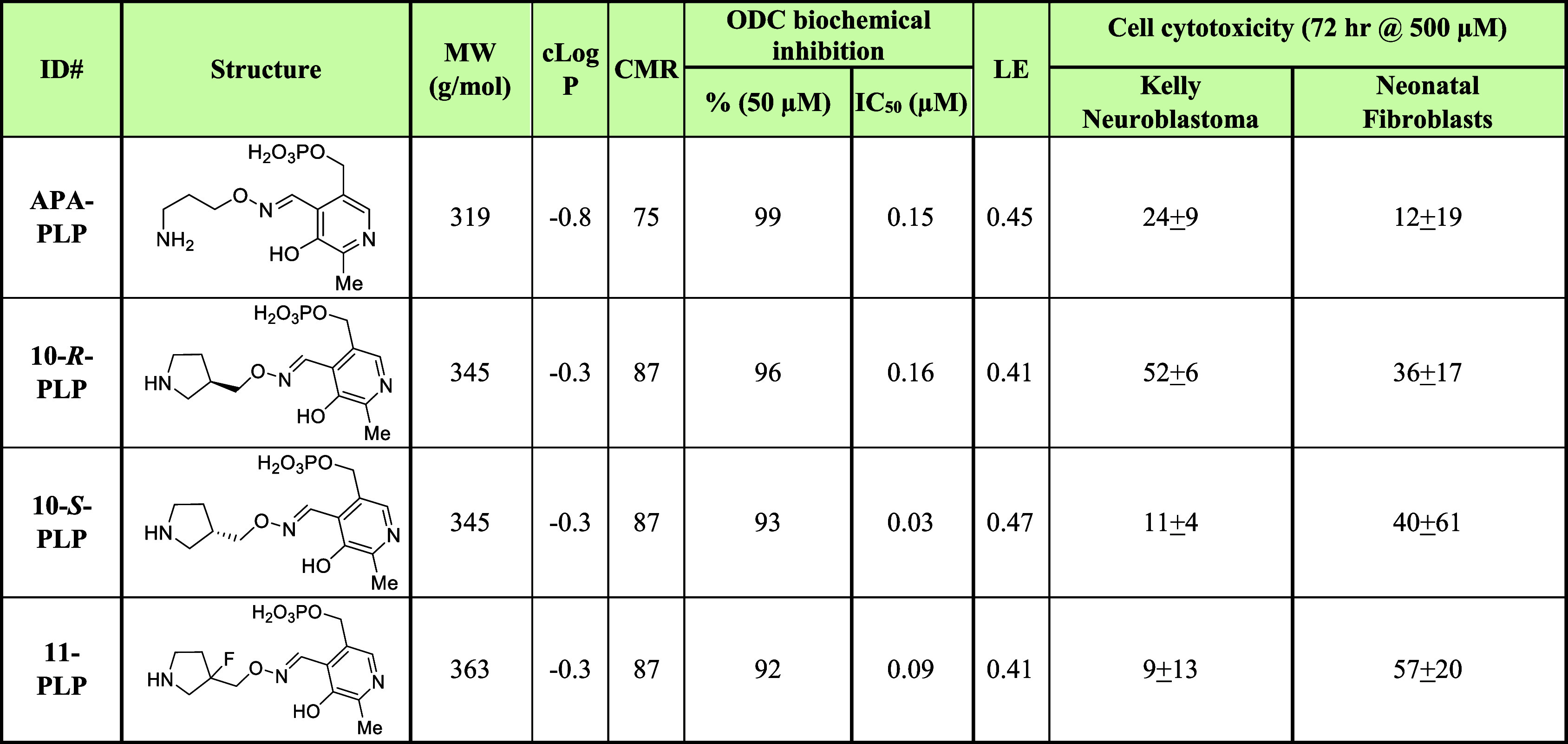
Structure-Activity Relationships (SAR) Studies Exploring Pyridoxal Phosphate (PLP) Adducts for Ornithine Decarboxylase (ODC) Activity^a^

a CMR = calculated molar refractivity; LE = ligand efficiency; cLog*P* = calculated logarithm of its partition coefficient between *n*-octanol and water; MW = Molecular Weight; IC_50_ = Inhibitory Concentration at 50%; APA = 1-amino-oxy-3-aminopropane; PLP = pyridoxal phosphate; ODC = ornithine decarboxylase.

We have, however, recently reported a new X-ray structure (7S3F) to human ODC in complex with **APA** at 1.7 Å resolution.^53^ In this case **APA** forms an oxime with PLP binding in the active site, a differentiating feature relative to that seen for **DFMO** (Figure 2) but consistent with the mechanism of the enzyme. This again is contrary to that noted in the X-ray structure of **APA**-ODC (*Leishmania donovani*) where oxime formation was not observed.^44^ Its lipophilic tether, between the oxime and the terminal amine, makes a lipophilic interaction with Tyr^389^. The terminal amine binds similarly to that seen in the reported X-ray structure of **DFMO** (2TOD),^45^ a H-bonding interaction with Asp^332^, and a second H-bonding interaction through a water to Asp^361^ and the hydroxyl of Tyr^361^ is also observed. Although there is the potential to make lipophilic interactions with Phe^397^, part of the cleft formed by the ODC monomers, it is not within distance to do so.

**DFMO** forms a covalent bond with the protein, leading to protein deactivation.**APA**, however, would not be considered an irreversible inhibitor as no covalent attachment to the protein is made, despite making a covalent attachment to PLP. The covalent attachment of an inhibitor to a cofactor in the active site of an enzyme represents, to the best of our knowledge, a novel observation and approach for enzyme inhibition, the covalent PLP adduct being the true inhibitor of the ODC enzyme.

Unfortunately, APA and the reported near-neighbor analogs are highly water-soluble and lack desirable drug-like properties^54^ to make it suitable for oral dosing. The reported series exhibits an unacceptably low calculated logarithm of its partition coefficient between *n*-octanol and water (cLog*P*) value, outside typical rule-of-5 compliance.^55^ Therefore, it, and its reported near-neighbor analogs, have not been further explored as a therapeutic. We herein report, an approach to overcome these weaknesses, taking advantage of a novel mechanism of action.

## Results

### APA-Related Studies

Our previous X-ray structure studies revealed that **APA** binds to ODC *via* the formation of an adduct between ODC and the cofactor PLP.^53^ A key question remaining was whether this adduct is formed in the active site or in solution prior to binding to the protein, there being a 10 to 50 μM concentration of PLP observed *in vivo* and in our *in vitro* ODC enzyme assay. To further explore, we prepared the adduct coupling **APA** to **PLP** (**APA-PLP**) and compared its IC_50_ vs **APA** (Tables 1 and 2), finding that it is indeed a potent inhibitor of ODC, with an inhibitory activity in a similar range. We then considered whether **APA-PLP** can be formed under the conditions of our *in vitro* ODC assay by excluding the enzyme. We saw no formation of the adduct **APA-PLP** under assay conditions (30 min at 37 °C) or even after stirring for 3 days. This supports the formation of the **APA-PLP** adduct as part of the catalytic cycle of the enzyme (Figure 1), able to be competitive with ornithine with a p*K*_a_ of ∼5.9.^52^ PLP itself, due to the phosphate ester, is unsuitable for inclusion into the design of a drug candidate, due to its ionization at pH 7, making it, in the absence of active uptake, impermeable. We have consequently pursued the novel approach of assembling potent inhibitors of ODC by the formation of the inhibitor adducts in the active site of the enzyme.

**APA** (Table 1)^56^ (WO9424094) exhibits an unacceptably low calculated cLogP value, outside the typical rule-of-5 compliance^57^ predicted to provide permeability and oral bioavailability to be a drug candidate. Permeability is typically seen for compounds having Log*P*s of 0.5–4. As the molecular weight (MW) of **APA** is relatively low, it is not considered to be a driver of permeability,^58^ which is typical for compounds having MWs greater than 500 g/mol.^55^ By contrast, relative to **APA**, **DFMO** is permeable due to an amino acid transporter.^59^ Assuming passive diffusion for new analogs of **APA**, we sought to increase the lipophilicity of new derivatives into more traditional drug-like space to favorably increase permeability^60^ without negatively impacting binding. The concept of centering the LogP into the desired range (0.5–4) to maximize permeability is well-documented.^61−64^

Ligand efficiency (LE) is a measure of binding energy/heavy atom for small molecule drugs. The typical values of drugs are ∼0.4–0.6.^65^ Although **APA** is a promising lead for the further development toward the identification of an antiproliferative drug,^66^ our X-ray structure of the **APA-PLP** adduct is the actual inhibitor.^53^ Taking this into account, this adduct (Table 2; ODC IC_50_ = 0.15 μM) (Figure 2) has a LE of 0.49, consistent with historical precedent for drugs.^67^

Calculated molar refractivity (CMR) is an alternative measure describing drug-like chemical space. The CMR of **APA** is outside the typical range (Table 1)^68−70^ of ∼40 to 130^71^ observed for most drugs. Increased molecular weight is a primary determinate for this parameter. The **APA-PLP** adduct demonstrates an improved CMR (Table 2), more typical of oral drug space, except for the liability of the phosphate ester functionality.^72^ The applicability of either LE or CMR to **APA**, for example, as “drug fragments” does not apply. Molecular weight, solubility, and cLogP, as drivers of permeability, are the key parameters for optimization.

To achieve the above, we explored the use of cyclic **APA**-like derivatives designed to maintain favorable interactions with the ODC binding site and to provide more favorable physical properties to enable permeability, the conformation restriction potentially limiting unwanted off pharmacology.^73−75^ They have increased molecular weight and cLog*P* values yet maintain water solubility. In the active site, these compounds form covalent attachments with PLP to inhibit the ODC enzyme with high potency. As a design feature, we sought to maintain the three-atom spacing between the basic terminal amine and the less basic –ONH_2_ present in **APA** (Table 1) also matching the α- to ε-amine length of **DFMO** and the natural substrate ornithine. We have also reasoned that using more rigid conformations of cyclic structures will enhance specificity for ODC relative to other PLP enzymes.

### Structure–Activity Relationships (SAR) Studies

Our strategy to identify novel ODC inhibitors was to first screen new analogs for ODC inhibition at 50 μM using our *in vitro* enzyme activity assay and purified human ODC (Table 1). Those showing significant inhibition were then titrated to provide an ODC IC_50_ followed by cell-based screening against both cancer (Kelly neuroblastoma) and noncancerous (neonatal fibroblasts) cell lines. For the cancer cell line, higher cytotoxicity was desired and for the noncancerous cell line counter screen, lower cytotoxicity was desired.

Exploring cyclic structures (Table 1), we first looked at the 4-membered azetidine-substituted **5**, finding it to be inactive, the compound likely constrained in an unfavorable orientation for binding. Moving to 6-membered rings, maintaining the 4-atom spacer theme of **APA**, we prepared both **6** and **7**. **6** demonstrated no ODC activity, while **7** was highly potent (ODC IC_50_ ∼ 0.18 μM). The enantiomers of compound **7**, **7**-*R* and **7**-*S* reproducibly produced similar IC_50_s at 0.77 and 0.70 μM, respectively. While neither enantiomer had superior activity relative to the other, they exhibited an unexplainably ∼4x lower activity relative to racemic **7**. Exploring further, compound **8** replaces the piperidine ring of **7** with a morpholine ring. It has similar ODC activity as that seen with **7**, **7**-*R* and **7**-*S*. Compound **9** was also prepared to probe the lipophilic binding channel and a potential H-bonding interaction with Asp^331^ as was seen in our X-ray structure of our **APA-PLP** adduct. Unfortunately, like **5** and **6**, it demonstrates no inhibitory activity against ODC.

We then turned our attention to the inclusion of the 5-membered pyrrolidine analogs. Compounds **10**, **10**-*R* and **10**-*S* had ODC IC_50_ values of 0.36, 0.21, and 0.10 μM, respectively. Like that observed for the piperidine derivatives (**7***-R* and *-S*), the IC_50_s values are only slightly differentiated favoring the *S*-enantiomer and in a range similar to that observed for **APA**. As before, it is anticipated that the PLP adducts are formed in the active site and are the true inhibitors. To help verify, we have also prepared **10**-*R*-**PLP** and **10**-*S*-**PLP** (Table 2) and found that they both demonstrate inhibition of ODC enzymatic activity, with IC_50_ values of 0.16 and 0.03 μM, respectively. The activity still favors, as observed for **10**-*R* and -*S*, the *S*-enantiomer with 8-fold relative activity.

To further explore, we prepared both racemic **11** (Table 1) and **11-PLP** (Table 2). Derivative **11** demonstrates an IC_50_ of 0.05 μM, similar to the previously reported fluorine containing acyclic analog,^50^ yet with greater lipophilicity. The corresponding **11-PLP** derivative reproducibly has a similar IC_50_ of 0.09 μM. This is in contrast to the corresponding PLP derivative of acyclic analog previously reported as having single digit μM activity in the enzyme assay.^50^ Relative to compounds **10**, **10**-*R* and **10**-*S*, we see an improvement is IC_50_ binding to ODC for compound **11** (racemate) and its PLP adduct, **11-PLP**.

### Modeling

To enable the modeling of new derivatives, we docked the **APA-PLP** adduct using the PDB: 2OO0 crystal structure^44^ with the Molecular Operating Environment (MOE). **APA-PLP** was placed into the active site of ODC through induced docking, and the highest ranked pose was used for analysis in MOE.^76−78^ The results were compared with our reported X-ray structure (PDB 7S3F)^53^ and our modeling of the **APA-PLP** poses were almost identical to the X-ray crystal structure. After this step, the analogs in Tables 1 and 2, with PLP adducts were also docked. To explore the quality of our modeling further, we compared the active analogs, forming the PLP adducts, and found a reasonable correlation between the *in vitro* activity of our analogs from Table 2 and their calculated binding energies, validating the approach (Supporting Information). For this validation, molecular dynamic simulations were performed with the **10**-*R* crystal structure for 30 ns with AMBER 2020 (https://ambermd.org/doc12/Amber20.pdf). After this step, the Molecular Mechanics Poisson–Boltzmann surface area (MM-PBSA)^79^ was utilized to assess binding free energies of the ligands with PLP. (More details on the simulation conditions and free binding energy calculations are provided in the Supporting Information).

**Figure 3:**
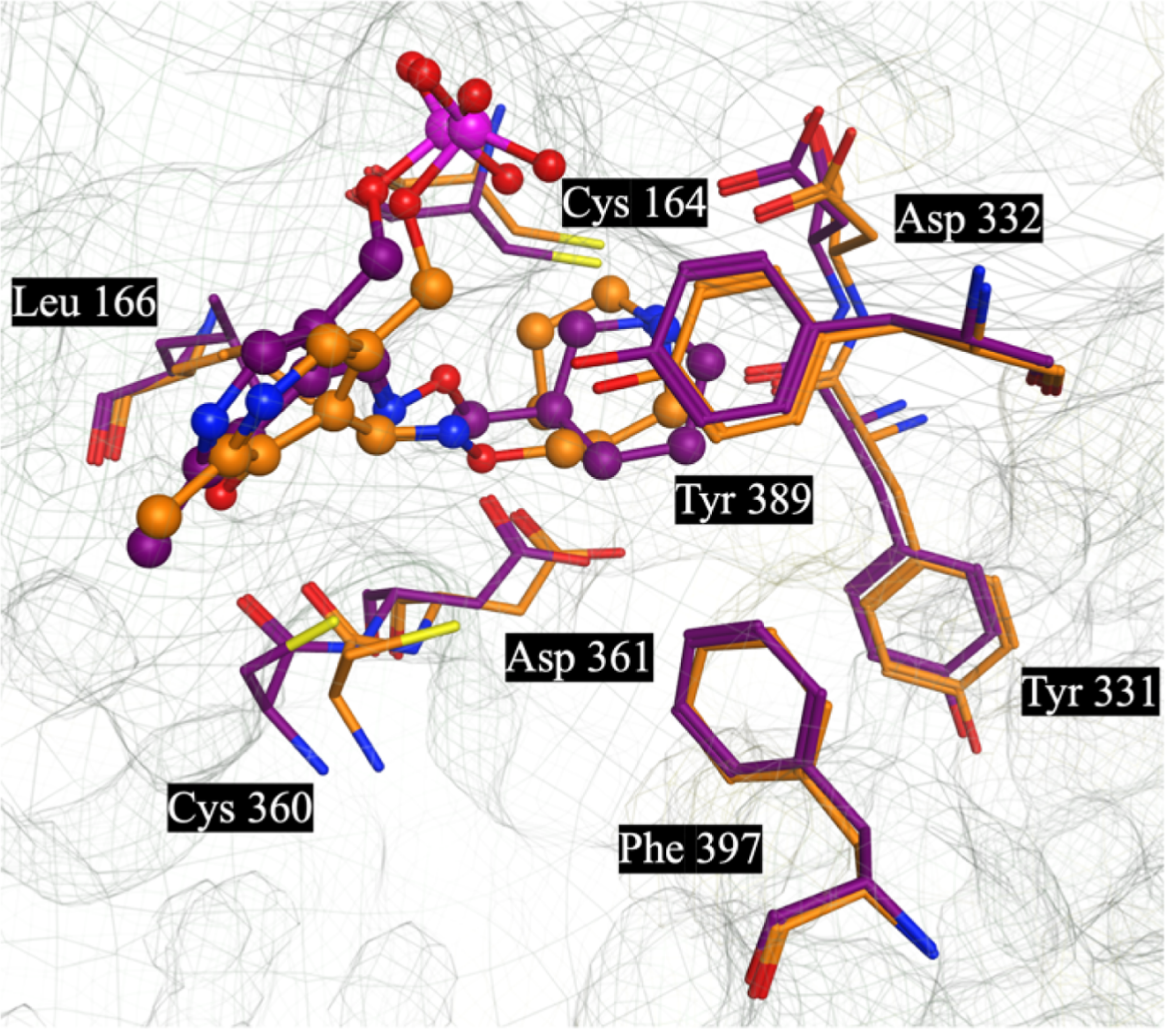
Modeled structures of **7**-*R*-**PLP** (Purple) and **7**-*S*-**PLP** (Orange) overlapped with one-another and their key interactions with human ornithine decarboxylase (ODC).

The less potent, relative to **APA-PLP**, modeled piperidine structures of **7**-*R*-**PLP** and **7**-*S*-**PLP** (Figure 3), beyond filling the PLP binding space, make key interactions between the terminal –NH_3_^+^ and lipophilic interactions stretching through the lipophilic “cradle” formed by PheB^397^ and TyrA^389^. For both, we see binding interactions with Tyr^389^ with the piperidine rings and some longer-range lipophilic interactions with Phe^397^. However, unlike **APA-PLP**, the constrained piperidines are unable to make H-bonding interactions with Asp^331^, which potentially explains the reduced ODC activities versus **APA-PLP**. Also, as the piperidine rings are solvent exposed, it becomes clear why compounds **7**-*R*, **7**-*S* and **8** demonstrate similar activities *in vitro*.

**Figure 4:**
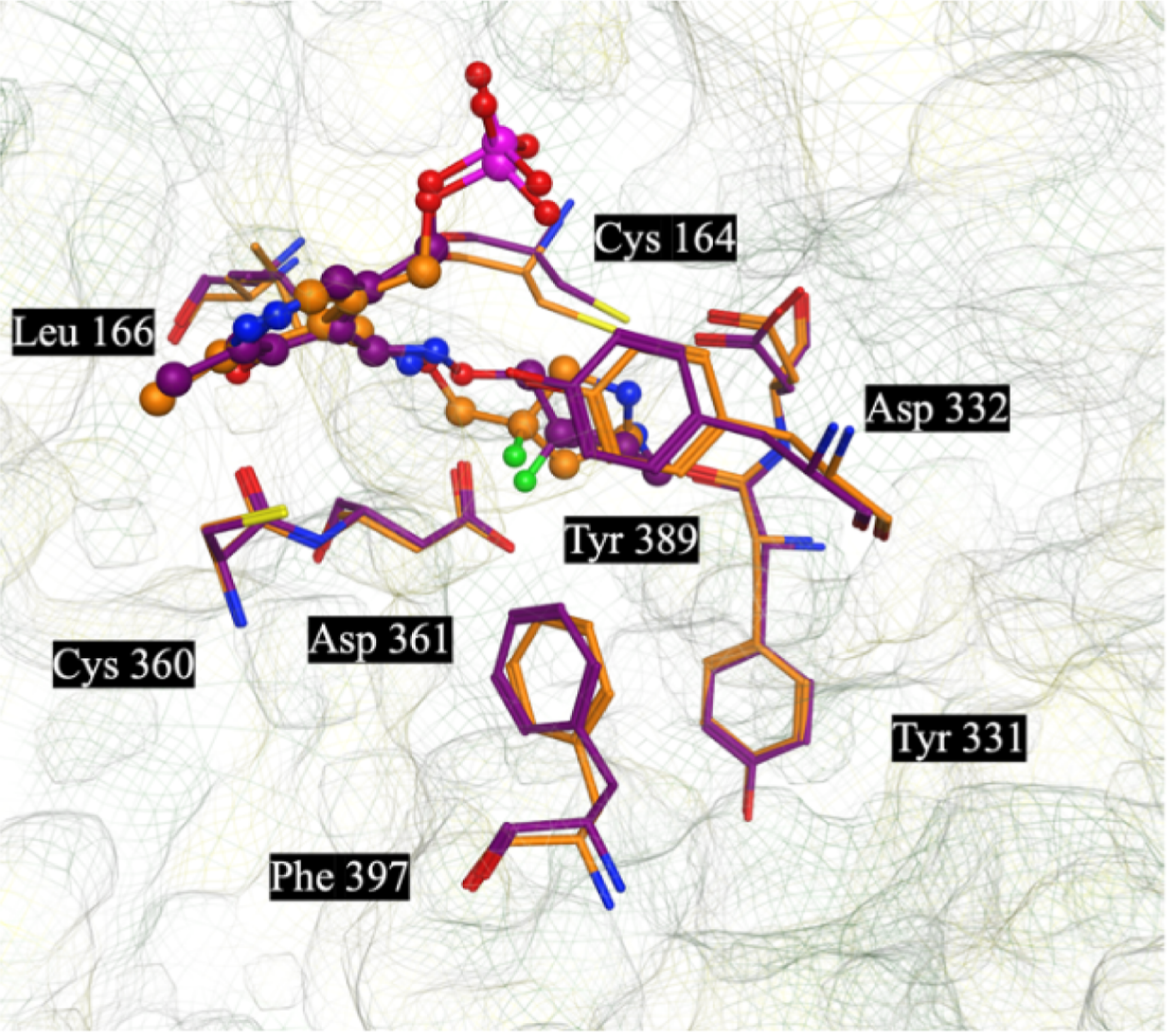
Modeled structures of **11**-*R*-**PLP** (Purple) and **11**-*S*-**PLP** (Orange) overlapped with one-another and their key interactions with human ornithine decarboxylase (ODC).

Similarly, the modeled structures of racemic **11-PLP**, modeled as **11**-*S*-**PLP and 11**-*R***-PLP** (Figure 4), using the same methodology, provide further insights into the binding of this analog. In Tables 1 and 2, racemic **11** and its PLP adduct **11-PLP** are highly potent, demonstrating activities similar to or better than **APA** and the **APA-PLP** adduct. The binding orientation is like that observed by the analogs described above but with an additional potential binding interaction, from the fluorine atom to the hydrogen of Phe^397^ (∼2.4 Å) for compound **11**-*S*, *i.e*., a potential new hydrogen bond.

### X-Ray Structures

**Figure 5:**
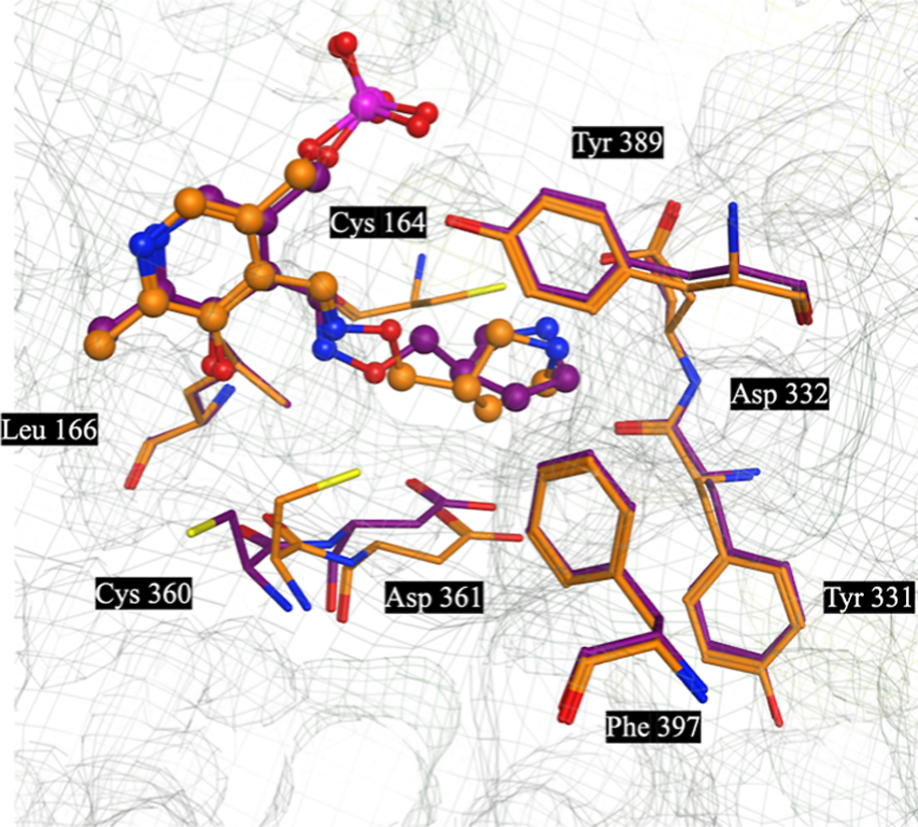
X-ray structures of **10**-*R*-**PLP** (Purple) (PDB: 9B8M) and **10**-*S*-**PLP** (Orange) (PDB: 9B8N) overlapped with one-another and their key interactions with human ornithine decarboxylase (ODC).

We prepared and refined two high-resolution X-ray structures from soaking experiments with **10**-***R*** (PDB: 9B8M) and **10**-*S* (PDB: 9B8N) with human ODC/PLP crystals (Figure 5) in the same fashion as we have described previously.^53^ In each case, as we saw with **APA**, the adducts **10**-*R*-**PLP** (2.9 Å resolution) and **10**-*S*-**PLP** (2.0 Å resolution) were formed, forming *trans*-oximes with PLP. The lipophilic cyclic pyrrolidines of **10**-*R*-**PLP** and -*S*-**PLP**, form strong lipophilic interactions with Tyr389 and weaker lipophilic interactions with Phe^397^, the pyrrolidine rings orientated in an orthogonal manner relative to each other. The basic pyrrolidine amine of each makes a H-bonding interaction with Asp^331^. The modeled structures are highly similar. Although the structure associated with **10**-*R*-**PLP** is of lower resolution and does not include water molecules in the structure, a well-defined H-bond network of direct interactions is available, which explains the similarity of activities observed. While there are some small differences between each structure, both networks appear to be equally strong.

### Cellular Activities

To investigate the cell-based activities of new analogs, we used Kelly neuroblastoma cells to assess anticancer activity and neonatal fibroblasts to address undesirable cytotoxicity (Table 1). **DFMO** demonstrates relatively moderate cytotoxic activity against Kelly neuroblastoma cells, and greater cytotoxic activity against neonatal fibroblasts. By comparison, **APA** exhibits greater anticancer activity with somewhat reduced overt cytotoxic activity. Our cyclic non-PLP-derivatives (**7**, **7***-R*, **7***-S*, **8**, **10**, **10**-*R*, **10**-*S*) all have greater cLog*P* values, molecular weights and are more rigid than **APA** suggesting more drug-like features. All demonstrate similar activities against Kelly neuroblastoma cells, despite differing levels of ODC inhibition between the analogs and modulated cLogPs, etc. All clearly demonstrate reduced cytotoxic activity against noncancerous neonatal fibroblasts relative to both **DFMO** and **APA** (Table 1). We also tested in the same cell-based assays our PLP adducts (**APA**-**PLP**, **10**-*R*-**PLP**, **10**-*S*-**PLP** and **11**-**PLP**), which demonstrated disappointing neuroblastoma cellular activities and were generally more toxic in neonatal fibroblasts. These activities are anticipated to be due to reduced permeability and membrane effects.

**Figure 6:**
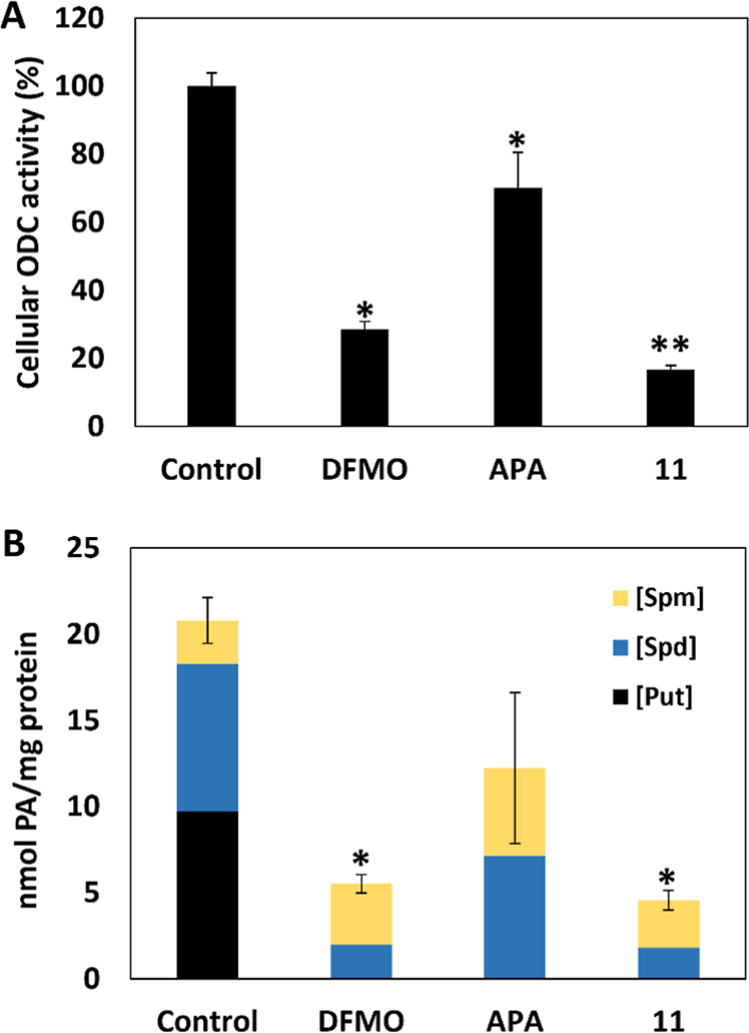
Kelly neuroblastoma cells treated with compound **11** have reduced endogenous ornithine decarboxylase (ODC) activity and polyamine levels. For the cell-based ODC activity (A), the data represents mean ± the standard error (S.E.) of at least three independent experiments done in duplicate (*N* = 6). For the polyamine pool analysis (B), the data represents the mean ± SE of at least three independent experiments (*N* = 3). *Denotes statistically significant reduction compared to control (*p* < 0.05). **Denotes statistically significant reduction compared to control, α-difluoromethylornithine (**DFMO**) and 1-amino-oxy-3-aminopropane (**APA**) (*p* < 0.05).

To mechanistically explore the cell-based activities of compound **11** further, we directly compared the inhibition of endogenous ODC activity by **DFMO**, **APA**, and **11** in Kelly neuroblastoma cells. Each significantly reduced the cellular ODC activity relative to control (Figure 6A). The greatest reduction, however, was observed with **11**. The reduced activity of **APA** was likely due to lower permeability associated with a lower cLogP relative to **11**. A direct response of ODC inhibition is also a measurable change in polyamine production (putrescine, spermidine, and spermine).^1,6,7,12,80^ All three compounds reduced polyamine levels in Kelly neuroblastoma cells, relative to control (Figure 6B). Putrescine was undetected for all three analogs. **DFMO** and **11**, however, had the greatest reductions in spermidine and spermine levels relative to **APA**. This is despite **APA** and **11** having similar IC_50_ values against the ODC enzyme. The superior cell-based activity of **11**, is likely associated with improved physical properties relative to APA.

### Synthesis of ODC Inhibitor Analogs

**Scheme 1:**
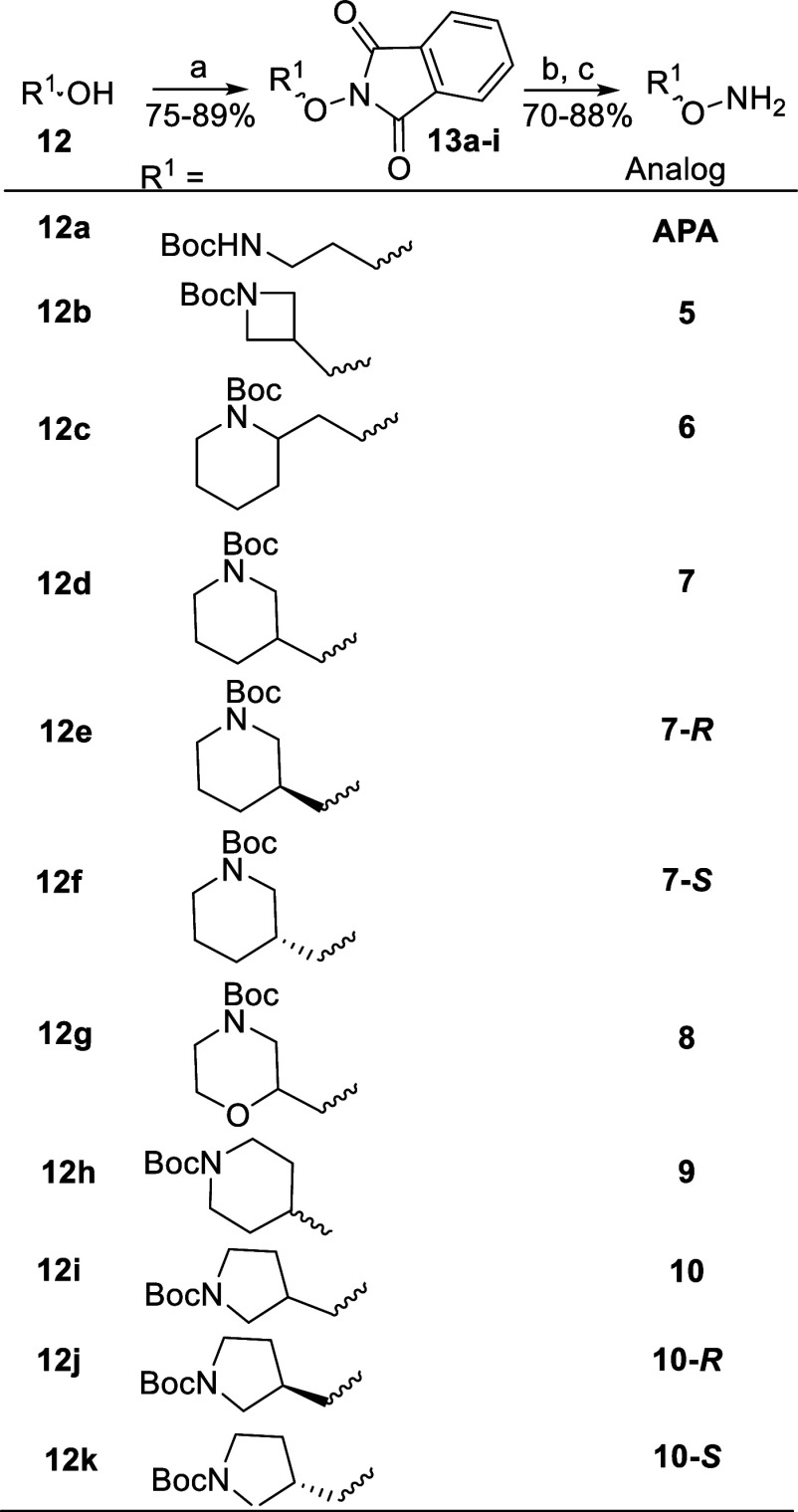
General Syntheses of 1-Amino-oxy-3-aminopropane (**APA**) and **APA**-like Ornithine Decarboxylase (ODC) Inhibitors (Compounds **5**, **6**, **7**, **7**-*R*, **7**-*S*, **8**, **9**, **10**, **10**-*R*, **10**-*S*) Reagents and conditions. (a) Ph_3_P, diisopropyl azodicarboxylate (DIAD), 2-hydroxy-1*H*-Isoindole-1,3(2*H*)-dione, tetrahydrofuran, 0 °C to RT; (b) hydrazine.H_2_O, dichloromethane; (c) HCl, dioxane, methanol.

**Scheme 2:**
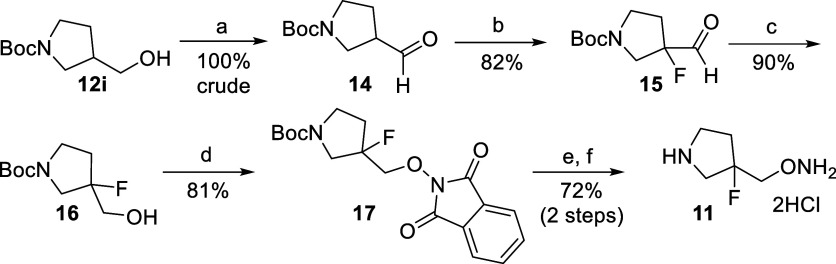
Preparation of Analog 11 Reagents and Condition. for the preparation of **11**: (a) DMP, dichloromethane; (b) pyrrolidine, MTBE, *N*-fluorobenzenesulfonimide, 40 °C; (c) NaBH_4_, MeOH; (d) Ph_3_P, diisopropyl azodicarboxylate (DIAD), 2-hydroxy-1*H*-isoindole-1,3(2*H*)-dione, tetrahydrofuran, 0 °C to RT; (e) hydrazine.H_2_O, dichloromethane; (f) HCl, dioxane, methanol.

**APA** and its cyclic analogs (Table 1) were prepared by the general synthetic process (Scheme 1) via a simple 3 step process from available Boc-protected amino alcohols **12a**–**k**, except for compound **11** (Scheme 2). The Boc-protected amino alcohols (**12a**–**k**) were coupled to 2-hydroxy-1*H*-isoindole-1,3(2*H*)-dione, via a Mitsunobu reaction,^81^ producing intermediates (**13a**–**k**) in high yields. These were then, in turn, treated with aqueous hydrazine and subsequently deprotected with 2.0 M HCl in dioxane to produce the compounds **5**,**6**, **6**-*R*, **6**-*S*, **7**, **8**, **9**, **9**-*R*, **9**-*S*, **10**, and **11** (Table 1) also in high yields. Compound **11** was synthesized from Boc-amino alcohol **17**, which was prepared as shown in Scheme 2.

**Scheme 3:**
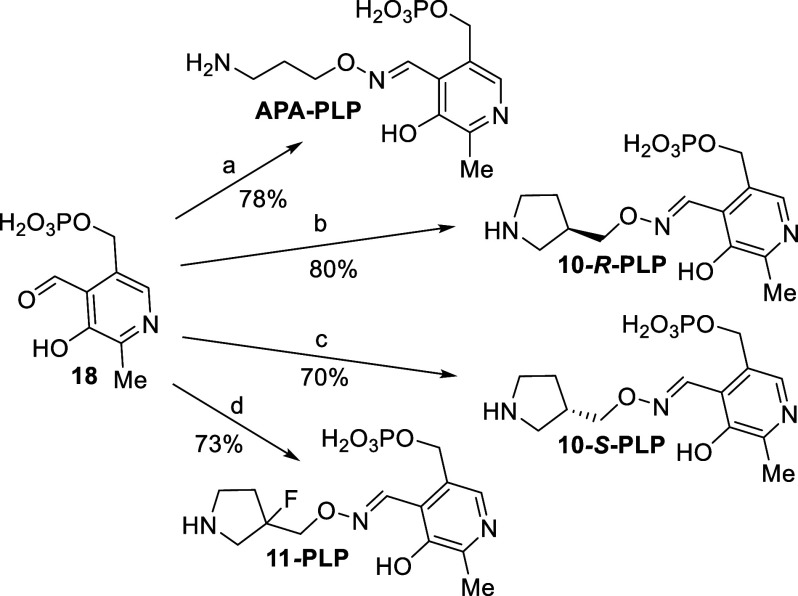
Preparation of Pyridoxal Phosphate (PLP) Adducts Reagents and conditions for the preparation of PLP adducts: (a) 1-amino-oxy-3-aminopropane (**APA**); (b) **10**-*R*, PBS buffer (pH 7.4); (c) **10**-*S*, PBS buffer (pH 7.4); (d) **11**, PBS buffer (pH 7.4).

To produce **11**, we started from commercially available **12g** where **14** was produced via oxidation, using the Dess–Martin periodinane in high yield. This intermediate was then fluorinated^82^ to produce **15**, which was then reduced to provide **16**, the overall yield being 74% over the 3 steps. Intermediate **16** was then converted to **11** in a similar fashion as shown in Scheme 1. The PLP-adducts (Table 2) were prepared according to Scheme 3 where **APA** analogs were each treated with pyridoxal phosphate (**18**). After reverse-phase purification, **APA-PLP**, **10**-*R*-**PLP**, **10**-*S*-**PLP and 11-PLP** were produced in high yields.

## Discussion and Conclusions

Although the oral formulation of **DFMO** and its clinical target ODC were recently validated through its FDA approval for pediatric and adult patients with high-risk neuroblastoma, high concentrations of **DFMO** are needed in tumor cell cultures (higher μM to low mM),^12,83^ cancer mouse models (1–2% w/v in drinking water)^14,84−86^ and patients (500–9000 mg/m^2^ BID)^27,28,31,32^ to achieve efficacy. The combination of low potency, against ODC, and modest pharmacokinetic properties, including rapid renal clearance, requires high **DFMO** concentrations to inhibit ODC and limits its attractiveness. We have consequently sought to design new ODC inhibitor analogs that can be developed into superior ODC inhibitors. The X-ray structures of ODC with bound inhibitors ligands, including **DFMO**, our new analogs, or putrescine, demonstrate the presence of PLP in the active side and a relatively narrow lipophilic channel, occupied by the hydrocarbon portions of these compounds, leading to a hydrophilic space with opportunities to bind to Asp^332^, Asp^331^ and the hydroxyl of Tyr^361^. Rather than being suicide inhibitors, like **DFMO**, we have uncovered a novel strategy for the inhibition of the PLP dependent ODC enzyme by forming irreversible adducts with PLP in the active site of the ODC enzyme, as part of the catalytic cycle. The active-site formed PLP adducts are the true inhibitors, as demonstrated from our X-ray structures, our *in vitro* experiments, and the ability of our prepared PLP adducts (Table 2) to bind and inhibit the enzyme. The design of new inhibitors is enabled by X-ray crystallography and modeling. To the best of our knowledge, this has never been studied as a design strategy, i.e., the formation of an inhibitor in the active site of an enzyme. This contrasts with the plethora of new inhibitors, now under study, which make covalent attachments to their therapeutic targets.^87^ The success of a covalent attachment design strategy is highly dependent on the ability of an inhibitor to bind to a target enzyme with high potency combined with the functionality enabling protein derivatization in the active site. This approach is demonstrated by **DFMO**, but it is simply a poor ODC binder (Table 1) having high micromolar IC_50_ activity. For ODC, the size of the binding domain limits the potential of identifying, from SAR studies, desirable low nanomolar inhibitory activity. The compounds of Table 1, when binding to ODC are differentiated from **DFMO** as they form covalent irreversible adducts in the active site of ODC. The resulting adducts are inhibitors with the potential of low nanomolar activities taking advantage of the expanded binding domain occupied by PLP. For ODC and other small “active site” targets, this approach is attractive, particularly in an environment where few options exist to prepare bioavailable and permeable mimics of PLP. The activities seen with this approach are superior to that seen for transition-state mimics reported by Wu and Gehring.^39^ Those reported analogs do not reduce polyamine levels as seen with **DFMO** and compound **11** (Figure 6B), despite demonstrating cytotoxic activity against cancer cell lines suggestive of off-target biology.

A key challenge of this new approach are the low molecular weights and nondrug-like physical properties of compounds like **APA**. We have found, however, that by structural modification, focused on both increasing the molecular weights and increasing the cLog*P*, analogs of **APA** (Table 1, analogs **7**–**11**) have improved drug-like physical properties favoring enhanced permeability. Specifically, compound **11** inhibits the ODC enzyme in neuroblastoma cells with greater activity than that seen by DFMO and **APA** (Figure 6). Its improved permeability due to an increased LogP, despite having a similar enzyme IC_50_ value relative to **APA**, enhances its ability to reduce polyamines, specifically putrescine and spermidine, which are essential for the suppression of neuroblastoma and other polyamine dependent diseases.^6,7,12,13,88−90^ The approach of constructing an inhibitor with a cofactor in the active site of ODC has implications for the design of other, difficult to target, PLP enzymes as therapeutic targets, having small active sites, where covalent attachment to a cofactor might generate a tightly bound inhibitor.^91^

The new ODC inhibitors designed in this study are significantly improved compared to **DFMO**, a low potency ODC inhibitor with poor PK properties and so far, the only FDA-approved ODC inhibitor in the clinic. While **DFMO** has an excellent safety profile and received FDA approval for West African sleeping sickness (trypanosomiasis), hirsutism, and cancer (neuroblastoma), additional uses for ODC inhibitors in the medical field are being identified, including rare genetic diseases (Bachmann-Bupp Syndrome, Snyder-Robinson Syndrome)^32,33,92,93^ and diabetes.^34^ Analogs, such as the more rigid, similarly potent, and more permeable compound **11**, relative to **APA**, demonstrate superior activity and mechanistically reduce polyamine syntheses when compared to both **DFMO** and **APA** (Figure 6A,B). This approach will be explored further. Finally, polyamines are known to impact the development of human viral diseases (Ebola, HIV, Dengue, etc.)^94−96^ and age-related diseases (Alzheimer’s disease, Parkinson’s disease, osteoarthritis, sarcopenia, and osteoporosis),^97^ thus warranting the further development and *in vivo* assessment of alternative ODC inhibitors, such as compound **11**, with superior physical properties and improved cellular potency.

## Experimental Section

### Instrumentation and Reagents

All commercial reagents and solvents were used as received. Anhydrous solvents were dried over 4 Ao molecular sieves. Thin layer chromatography (TLC) was performed on silica gel 60G F254 glass plates. ^1^H NMR and ^13^C NMR spectra were recorded on an Agilent VXR (500 MHz). Purity for final compounds is greater than 95% by NMR. Purity of PLP analogs was measured using Agilent 1200 series high performance liquid chromatography (HPLC) systems with UV detection at 214 nm and using a Delta-Pak 300 × 3.9 mm C18 15 μm, 300 Å column with isocratic elution at 15% methanol in water. High resolution mass spectra were recorded with a Waters G2-XS-QTof using an electrospray ionization (ESI) mode. Optical rotations were measured on a PerkinElmer polarimeter (concentration (c) is given as g/mL). The ODC inhibitor **DFMO** was provided by the late Dr. Patrick Woster (Medical University of South Carolina, Charleston, SC). Reactions were monitored using TLC on precoated silica gel plates (Merck Silica Gel 60 F254) or by liquid chromatography/mass spectrometry (LC/MS) (Advion A-2053, Advion Express-CMS).

### Compound Syntheses

All compounds are >95% pure by NMR analysis and similarly by HPLC where appropriate.

### *tert*-Butyl {3-[(1,3-Dioxo-1,3-dihydro-2*H*-isoindol-2-yl)oxy]propyl}carbamate

A mixture of 3-(*tert*-butoxycarbonylamino)-1-propanol (1.0 g, 5.71 mmol), *N*-hydroxyphthalimide (1.03 g, 6.28 mmol) and triphenylphosphine (1.60 g, 6.28 mmol) in dry tetrahydrofuran (20 mL), under an argon atmosphere, was stirred at 0 °C for 20 min. A solution of diisopropyl azodicarboxylate (1.30 g, 6.28 mmol) in tetrahydrofuran (10 mL) was then added dropwise and allowed to slowly warm to room temperature (2 h). Upon completion, the reaction was concentrated in vacuo. The resulting residue was purified via medium pressure liquid chromatography (SiO_2_, 100% hexanes to 60% ethyl acetate/hexanes (1.54 g, 84% yield). ^1^H NMR (500 MHz, Chloroform-*d*): δ 7.84 (dd, *J* = 5.4, 3.1 Hz, 2H), 7.76 (dd, *J* = 5.5, 3.1 Hz, 2H), 4.27 (t, *J* = 5.9 Hz, 2H), 3.42 (q, *J* = 6.4 Hz, 2H), 1.95 (q, *J* = 6.1 Hz, 2H), 1.44 (s, 9H).

### 3-(Aminooxy)propan-1-Amine Dihydrochloride (APA)^98^

To a solution of *tert*-butyl {3-[(1,3-dioxo-1,3-dihydro-2*H*-isoindol-2-yl)oxy]propyl}carbamate (1.0 g, 3.13 mmol) in dry dichloromethane (15 mL), under an argon atmosphere, was added hydrazine monohydrate (0.30 mL, 3.13 mmol) dropwise during 5 min. The solution turned into a suspension in 30 min. After 2 h, the white precipitate was filtered off and washed with cold dichloromethane (10 mL). The filtrate was concentrated in vacuo and the resulting residue was used without further purification. The residue was taken up in methanol (5 mL) and treated dropwise with hydrochloric acid (4.0 N in dioxane, 4.0 mL, 16.0 mmol). The mixture was stirred at rt, under an argon atmosphere, for 2 h and concentrated. The resulting residue was taken up in water (10 mL), washed with ethyl acetate (3 × 5 mL) and lyophilized to give the di-HCl salt of 3-(aminooxy)propan-1-amine (**4**) as a semisolid hygroscopic material (0.40 g, 78% yield for two steps). ^1^H NMR (500 MHz, D_2_O): δ 4.03 (t, *J* = 5.9 Hz, 2H), 2.96 (t, *J* = 7.5 Hz, 2H), 1.91 (p, *J* = 6.4 Hz, 2H). ^13^C NMR (126 MHz, D_2_O): δ 72.32, 36.54, 25.14. HRMS (ESI) *m*/*z* calcd for C3H_11_N_2O_ [M + H], 91.0871; found, 91.0879.

### ({4-[(*E*)-[(3-Aminopropoxy)imino]methyl]-5-hydroxy-6-methylpyridin-3-yl}methoxy)phosphonic Acid (APA-PLP)

To a solution of 3-(aminooxy)propan-1-amine-di-HCl salt (**APA**) (0.105 g, 0.65 mmol) in PBS buffer (2.0 mL, pH 7.40) was added pyridoxal 5′-phosphate **2** (0.16 g, 0.65 mmol) in PBS buffer (2.0 mL, pH 7.40). The resulting mixture was stirred at rt for 18 h. Subsequently, the reaction mixture was passed through a sintered glass filter. The filtrate was lyophilized to give the title compound as a pasty solid material (0.16 g, 78% yield). ^1^H NMR (500 MHz, D_2_O): δ 8.60–8.56 (m, 1H), 8.05 (d, *J* = 0.8 Hz, 1H), 4.96 (d, *J* = 7.7 Hz, 2H), 4.35 (t, *J* = 5.8 Hz, 2H), 3.02 (t, *J* = 7.4 Hz, 2H), 2.52 (s, 3H), 2.05–1.96 (m, 2H). ^13^C NMR (126 MHz, D_2_O): δ 152.78, 146.74, 144.72, 133.31, 133.25, 129.75, 127.31, 73.13, 61.68, 61.65, 36.86, 26.11, 14.24. HRMS (ESI) *m*/*z* calcd for C_11_H_19_N_3_O_6_P [M + H], 320.1011; found, 320.1045. Melting point: 160 °C (Decomposition). HPLC purity 96.7% contaminated with ∼3% ({4-[(*Z*)-[(3-aminopropoxy)imino]methyl]-5-hydroxy-6-methylpyridin-3-yl}methoxy)phosphonic acid observed by ^1^HNMR and HPLC.

### *tert*-Butyl 3-{[(1,3-Dioxo-1,3-dihydro-2*H*-isoindol-2-yl)oxy]methyl}piperidine-1-carboxylate

A mixture of *tert*-butyl 3-(hydroxymethyl)piperidine-1-carboxylate (2.0 g, 9.29 mmol), *N*-hydroxyphthalimide (1.70 g, 10.22 mmol) and triphenylphosphine (2.70 g, 10.22 mmol) in dry tetrahydrofuran (40 mL), under an argon atmosphere, was stirred at 0 0C for 20 min. A solution of diisopropyl azodicarboxylate (2.10 g, 10.22 mmol) in tetrahydrofuran (20 mL) was then added dropwise and allowed to slowly warm to room temperature (2 h). Upon completion, the reaction was concentrated in vacuo. The resulting residue was purified via medium pressure liquid chromatography (SiO_2_, 100% hexanes to 60% ethyl acetate/hexanes (3.0 g, 89% yield) to provide the title compound. ^1^H NMR (500 MHz, chloroform-*d*): δ 7.83 (dd, *J* = 5.4, 3.1 Hz, 2H), 7.74 (dd, *J* = 5.5, 3.1 Hz, 2H), 4.06 (m, 2H), 3.87 (m, 1H), 2.93–2.78 (m, 2H), 2.08–1.93 (m, 1H), 1.70 (m, 1H), 1.53–1.34 (m, 4H), 1.46 (s, 9H).

### 3-[(Aminooxy)methyl]piperidine Dihydrochloride (**7**)

To a solution of *tert*-butyl 3-{[(1,3-dioxo-1,3-dihydro-2H-isoindol-2-yl)oxy]methyl}piperidine-1-carboxylate (3.0 g, 8.34 mmol) in dry dichloromethane (42 mL), under an argon atmosphere, was added hydrazine monohydrate (0.70 mL, 8.75 mmol) dropwise during 5 min. The solution turned into a suspension in 30 min. After 2 h, the white precipitate was filtered off and washed with cold dichloromethane (20 mL). The filtrate was concentrated in vacuo and the resulting residue was used without further purification. The residue was taken up in methanol (10 mL) and treated dropwise with hydrochloric acid (4.0 N in dioxane, 10.50 mL, 41.70 mmol). The mixture was stirred at rt, under an argon atmosphere, for 2 h and concentrated. The resulting residue was taken up in water (20 mL), washed with ethyl acetate (3 × 10 mL) and lyophilized to give the di-HCl salt of 3-[(aminooxy)methyl]piperidine (7) as a fine white powder (1.29 g, 76% yield for two steps). ^1^H NMR (500 MHz, D_2_O): δ 3.91 (dd, *J* = 8.5, 5.0 Hz, 1H), 3.83 (dd, *J* = 8.6, 7.1 Hz, 1H), 3.35–3.28 (m, 1H), 3.27–3.20 (m, 1H), 2.81–2.65 (m, 2H), 2.14–2.01 (m, 1H), 1.81 (dq, *J* = 13.8, 3.3 Hz, 1H), 1.74 (ddt, *J* = 15.3, 4.0, 2.0 Hz, 1H), 1.57 (dtt, *J* = 14.5, 12.9, 4.0 Hz, 1H), 1.23 (qd, *J* = 12.7, 3.9 Hz, 1H). ^13^C NMR (126 MHz, D_2_O): δ 76.03, 45.42, 43.98, 32.38, 23.93, 21.17. HRMS (ESI) *m*/*z* calcd for C_6_H_15_N_2O_ [M + H], 131.1184; found, 131.1189.

Chemical Procedures for remaining analogs are listed in Supporting Information

### ODC Enzyme Activity Assay

*In vitro* ODC activity was measured using 100 ng of purified human ODC enzyme, as we previously described.^53,99^ ODC was incubated with increasing concentrations of inhibitors for 30 min at room temperature in a buffer containing 25 mM Tris and 0.1 mM EDTA. Each of the reactions was added to 200 μL of assay mix containing 6.25 mM Tris HCl (pH 7.5), 100 μM l-ornithine, 50 μM pyridoxal-5-phosphate, 1.56 mM DTT and 0.1 μCi [1–14C] l-ornithine (American Radiolabeled Chemicals, Inc., specific activity 55 mCi/mmol) in a microcentrifuge tube. In the cell-based ODC activity assay, Kelly neuroblastoma cells that were treated with 500 μM inhibitors were harvested in a buffer containing 25 mM Tris HCl, 0.1 mM EDTA, and 2.5 mM DTT. Fifty microliters of each homogenate were added to 200 μL of assay mix containing 6.25 mM tris HCl (pH 7.5), 500 μM l-ornithine, 50 μM pyridoxal-5-phosphate, 1.56 mM DTT and 0.1 μCi [1–14C] l-ornithine (American Radiolabeled Chemicals, Inc., specific activity 55 mCi/mmol) in a microcentrifuge tube. For both the *in vitro* and cell-based assay, the microcentrifuge tubes were then placed into scintillation vials containing a piece of filter paper saturated with 200 μL of 0.1 M NaOH to capture the release of radiolabeled carbon dioxide. The samples were incubated in a 37 °C incubator while shaking for 30 min (*in vitro*) or 2 h (cell-based). The enzymatic reaction was stopped by adding 250 μL of 5 M sulfuric acid to each sample and incubating at 37 °C while shaking for 30 min. The microcentrifuge tubes were removed from the scintillation vials, and 5 mL of scintillation fluid was added. Disintegrations per minute (DPM) of each sample was measured using a TriCarb liquid scintillation counter (PerkinElmer). The specific ODC activity is expressed as nmol CO_2_/min/mg protein (*in vitro*) or pmol CO_2_/min/mg protein (cell-based). The individual ODC inhibitor IC_50_ values were determined using Graphpad Prism 8.

### Polyamine Analysis

Polyamines from Kelly neuroblastoma cells treated with 500 μM inhibitors were isolated, dansylated, and analyzed by HPLC. Briefly, polyamines were extracted and protonated in perchloric acid/sodium chloride buffer. To 100 μL of sample, 4.5 nmol of 1,7 diaminoheptane internal standard and 200 μL of 1 M sodium carbonate was added prior to dansylation with 400 μL of 5 mg/mL dansyl chloride (Sigma-Aldrich) for 60 min at 65 °C. One hundred μL of 1 M proline was then added to each reaction and incubated for 20 min at 65 °C. Polyamines were extracted from the mixture using methylene chloride. The methylene chloride was allowed to evaporate, the samples were resuspended in methanol and run through a C18 cleanup column. Samples were then analyzed using a Thermo Scientific/Dionex Ultimate 3000 HPLC, using a Syncronis C18 column (250 × 4.6 mm, 5 μM pore size), and containing a fluorescence detector. The dansylated polyamine peaks were visualized by excitation at 340 nM and emission at 515 nM. The buffers used for elution of the polyamines were as follows: buffer A: 10 mM sodium heptanesulfonate/10% acetontrile at pH 3.4. Buffer B: 100% acetonitrile. The flow rate was 0.7 mL per minute. The column was equilibrated with 55% buffer B/45% buffer A prior to injection of the sample. Upon injection of the sample, elution of polyamines occurs in an isocratic solution of 55% buffer B/45% buffer A for 28 min. The solution was then slowly ramped to 95% buffer B/5% buffer A for 20 min. This gradient was held for 27 min to elute the spermine peak. The solution was then quickly dropped back to 55% buffer B/45% buffer A to re-equilibrate the column prior to the next sample being injected. Using the relative molar response derived from *N*-dansylated PA and 1,7 diaminoheptane standards, the amount of *N*-dansylated polyamines was calculated and normalized to total sample protein.

### Cell Culture

Normal primary human neonatal fibroblast cells (ATCC) were maintained in DMEM medium (Gibco) supplemented with 10% heat-inactivated fetal bovine serum (Hyclone), penicillin (100 IU/mL), and streptomycin (100 μg/mL), nonessential amino acids (0.1 mM) and sodium pyruvate (1 mM). Kelly neuroblastoma cells (Sigma-Aldrich) were maintained in RPMI medium (Corning) supplemented with 10% heat-inactivated fetal bovine serum (Hyclone), penicillin (100 IU/mL), and streptomycin (100 μg/mL). Cell lines were routinely monitored for mycoplasma contamination using the MycoAlert PLUS Mycoplasma Detection Kit (Lonza).

### Sulforhodamine B Assay

The colorimetric sulforhodamine B (SRB) assay was used to measure cytotoxicity following treatment with ODC inhibitors. Briefly, Kelly neuroblastoma cells and normal primary human neonatal fibroblast cells were plated in transparent flat 96-well plates and allowed to attach overnight. Cells were treated with control or 500 μM ODC inhibitor for 72 h. Cells were then fixed with 10% TCA at 4 °C for 1 h, washed with deionized water, and dried at room temperature. Cells were stained with 100 μL of 0.4% SRB in 1% acetic acid for 20 min at room temperature, rinsed five times with 1% acetic acid and allowed to dry at room temperature. One hundred μL of 10 mM Tris–HCl pH 7.0 was added to each well, shaken for 10 min at room temperature and absorbance was read at 540 nM. Absorbance values were normalized relative to control readings and converted to percentage by multiplying by one hundred. The percent cytotoxicity was then determined by subtracting the relative percentage of the ODC inhibitor treated readings from the control which is 100%.

### Statistical Analyses

The statistical significance for changes in ODC activity and polyamine levels of Kelly cells treated with ODC inhibitors was determined using an unpaired Student’s *t*-test assuming the null hypothesis. For all tests, a value of *P* < 0.05 was considered statistically significant.

## Supporting Information Available

ODC protein expression and purification, ODC protein crystallization, crystal data collection and structure determination and analysis, analog syntheses, computational modeling validation (PDF)
Molecular formula strings (CSV)
7R_docking_pose.pdb (PDB)
7S_docking_pose.pdb (PDB)
8R_docking_pose.pdb (PDB)
8S_docking_pose.pdb (PDB)
8S_docking_pose.pdb (PDB)
10S_docking_pose.pdb (PDB)
11R_docking_pose.pdb (PDB)
11S_docking_pose.pdb (PDB)
APA_docking_pose.pdb (PDB)

## Supplementary Material

jm4c03120_si_002.csv

jm4c03120_si_003.pdb

jm4c03120_si_004.pdb

jm4c03120_si_005.pdb

jm4c03120_si_006.pdb

jm4c03120_si_007.pdb

jm4c03120_si_008.pdb

jm4c03120_si_009.pdb

jm4c03120_si_010.pdb

jm4c03120_si_011.pdb

## Abbreviations

APA: 1-amino-oxy-3-aminopropane
ATCC: American type culture collection
BID: bis in die (twice a day)
cLogP: calculated logarithm of its partition coefficient between *n*-octanol and water
DFMO: α-difluoromethylornithine
ESI: electrospray ionization
FDA: U.S. Food and Drug Administration
HPLC: high performance liquid chromatography
LC/MS: liquid chromatography/mass spectrometry
MYC: myelocytomatosis protein
ODC: ornithine decarboxylase
PLP: pyridoxal phosphate
SRB: sulforhodamine B
TLC: think layer chromatography
*T. brucei*: *Trypanosoma brucei*.

## References

[ref1] Wallace H. M.; Fraser A. V.; Hughes A. A perspective of polyamine metabolism. Biochem. J. 2003, 376 (1), 1–14. 10.1042/bj20031327. 13678416 PMC1223767

[ref2] Pegg A. E. Mammalian polyamine metabolism and function. IUBMB Life 2009, 61 (9), 880–894. 10.1002/iub.230. 19603518 PMC2753421

[ref3] Pegg A. E. Functions of Polyamines in Mammals. J. Biol. Chem. 2016, 291 (29), 14904–14912. 10.1074/jbc.R116.731661. 27268251 PMC4946908

[ref4] Pasini A.; Caldarera C. M.; Giordano E. Chromatin remodeling by polyamines and polyamine analogs. Amino Acids 2014, 46 (3), 595–603. 10.1007/s00726-013-1550-9. 23836422

[ref5] Matthews H. R. Polyamines, chromatin structure and transcription. Bioessays 1993, 15 (8), 561–566. 10.1002/bies.950150811. 8135771

[ref6] Casero R. A.; Marton L. J. Targeting polyamine metabolism and function in cancer and other hyperproliferative diseases. Nat. Rev. Drug Discovery 2007, 6 (5), 373–390. 10.1038/nrd2243. 17464296

[ref7] Casero R. A. Jr; Murray Stewart T.; Pegg A. E. Polyamine metabolism and cancer: treatments, challenges and opportunities. Nat. Rev. Cancer 2018, 18, 681–695. 10.1038/s41568-018-0050-3. 30181570 PMC6487480

[ref8] Holbert C. E.; Cullen M. T.; Casero R. A. Jr; Stewart T. M. Polyamines in cancer: integrating organismal metabolism and antitumour immunity. Nat. Rev. Cancer 2022, 22 (8), 467–480. 10.1038/s41568-022-00473-2. 35477776 PMC9339478

[ref9] Gerner E. W.; Meyskens F. L. Jr Polyamines and cancer: old molecules, new understanding. Nat. Rev. Cancer 2004, 4 (10), 781–792. 10.1038/nrc1454. 15510159

[ref10] Bello-Fernandez C.; Cleveland J. L. c-myc transactivates the ornithine decarboxylase gene. Curr. Top. Microbiol. Immunol. 1992, 182, 445–452. 10.1007/978-3-642-77633-5_56. 1490383

[ref11] Bello-Fernandez C.; Packham G.; Cleveland J. L. The ornithine decarboxylase gene is a transcriptional target of c-Myc. Proc. Natl. Acad. Sci. U.S.A. 1993, 90 (16), 7804–7808. 10.1073/pnas.90.16.7804. 8356088 PMC47231

[ref12] Wallick C. J.; Gamper I.; Thorne M.; Feith D. J.; Takasaki K. Y.; Wilson S. M.; Seki J. A.; Pegg A. E.; Byus C. V.; Bachmann A. S. Key role for p27Kip1, retinoblastoma protein Rb, and MYCN in polyamine inhibitor-induced G1 cell cycle arrest in MYCN-amplified human neuroblastoma cells. Oncogene 2005, 24 (36), 5606–5618. 10.1038/sj.onc.1208808. 16007177

[ref13] Bachmann A. S.; Geerts D. Polyamine synthesis as a target of MYC oncogenes. J. Biol. Chem. 2018, 293 (48), 18757–18769. 10.1074/jbc.TM118.003336. 30404920 PMC6290138

[ref14] Bassiri H.; Benavides A.; Haber M.; Gilmour S. K.; Norris M. D.; Hogarty M. D. Translational development of difluoromethylornithine (DFMO) for the treatment of neuroblastoma. Transl. Pediatr. 2015, 4 (3), 226–238. 10.3978/j.issn.2224-4336.2015.04.06. 26835380 PMC4729051

[ref15] Metcalf B. W.; Bey P.; Danzin C.; Jung M. J.; Casara P.; Vevert J. P. Catalytic irreversible inhibition of mammalian ornithine decarboxylase (E.C. 4.1.1.17) by substrate and product analogs. J. Am. Chem. Soc. 1978, 100, 2551–2553. 10.1021/ja00476a050.

[ref16] Bachmann A. S.; Geerts D.; Sholler G. Neuroblastoma: Ornithine decarboxylase and polyamines are novel targets for therapeutic intervention. In Pediatric Cancer, Neuroblastoma: Diagnosis, Therapy, and Prognosis, 1 ed.; Hayat M. A., Ed.; Springer, 2012; pp 91–103.

[ref17] Bachmann A. S.; Levin V. A. Clinical applications of polyamine-based therapeutics. In Polyamine Drug Discovery; Woster P. M., Casero R. A., Eds.; Royal Society of Chemistry, 2012; pp 257–276. .

[ref18] Franco J.; Simarro P. P.; Diarra A.; Ruiz-Postigo J. A.; Samo M.; Jannin J. G. Monitoring the use of nifurtimox-eflornithine combination therapy (NECT) in the treatment of second stage gambiense human African trypanosomiasis. Res. Rep. Trop Med. 2012, 3, 93–101. 10.2147/RRTM.S34399. 30100776 PMC6067772

[ref19] Alirol E.; Schrumpf D.; Amici Heradi J.; Riedel A.; de Patoul C.; Quere M.; Chappuis F. Nifurtimox-eflornithine combination therapy for second-stage gambiense human African trypanosomiasis: Medecins Sans Frontieres experience in the Democratic Republic of the Congo. Clin. Infect. Dis. 2013, 56 (2), 195–203. 10.1093/cid/cis886. 23074318

[ref20] Priotto G.; Fogg C.; Balasegaram M.; Erphas O.; Louga A.; Checchi F.; Ghabri S.; Piola P. Three drug combinations for late-stage Trypanosoma brucei gambiense sleeping sickness: a randomized clinical trial in Uganda. PLoS Clin. Trials 2006, 1 (8), e39 10.1371/journal.pctr.0010039. 17160135 PMC1687208

[ref21] Priotto G.; Kasparian S.; Mutombo W.; Ngouama D.; Ghorashian S.; Arnold U.; Ghabri S.; Baudin E.; Buard V.; Kazadi-Kyanza S.; et al. Nifurtimox-eflornithine combination therapy for second-stage African Trypanosoma brucei gambiense trypanosomiasis: a multicentre, randomised, phase III, non-inferiority trial. Lancet 2009, 374 (9683), 56–64. 10.1016/S0140-6736(09)61117-X. 19559476

[ref22] Alirol E.; Schrumpf D.; Amici Heradi J.; Riedel A.; de Patoul C.; Quere M.; Chappuis F. Nifurtimox-Eflornithine Combination Therapy for Second-Stage Gambiense Human African Trypanosomiasis: Médecins Sans Frontières Experience in the Democratic Republic of the Congo. Clin. Infect. Dis. 2013, 56, 195–203. 10.1093/cid/cis886. 23074318

[ref23] Malhotra B.; Noveck R.; Behr D.; Palmisano M. Percutaneous absorption and pharmacokinetics of eflornithine HCl 13.9% cream in women with unwanted facial hair. J. Clin. Pharmacol. 2001, 41 (9), 972–978. 10.1177/00912700122010951. 11549102

[ref24] Blume-Peytavi U.; Hahn S. Medical treatment of hirsutism. Dermatol. Ther 2008, 21 (5), 329–339. 10.1111/j.1529-8019.2008.00215.x. 18844711

[ref25] Meyskens F. L. Jr; McLaren Chemoprevention, risk reduction, therapeutic prevention, or preventive therapy?. J. Natl. Cancer Inst. 2010, 102 (24), 1815–1817. 10.1093/jnci/djq466. 21115881

[ref26] Meyskens F. L. Jr; McLaren C. E.; Pelot D.; Fujikawa-Brooks S.; Carpenter P. M.; Hawk E.; Kelloff G.; Lawson M. J.; Kidao J.; McCracken J.; et al. Difluoromethylornithine plus sulindac for the prevention of sporadic colorectal adenomas: a randomized placebo-controlled, double-blind trial. Cancer Prev. Res. 2008, 1 (1), 32–38. 10.1158/1940-6207.CAPR-08-0042. PMC2562024 18841250

[ref27] Oesterheld J.; Ferguson W.; Kraveka J. M.; Bergendahl G.; Clinch T.; Lorenzi E.; Berry D.; Wada R. K.; Isakoff M. S.; Eslin D. E.; et al. Eflornithine as Postimmunotherapy Maintenance in High-Risk Neuroblastoma: Externally Controlled, Propensity Score-Matched Survival Outcome Comparisons. J. Clin. Oncol. 2024, 42, 90–102. 10.1200/JCO.22.02875. 37883734 PMC10730038

[ref28] Saulnier Sholler G. L.; Gerner E. W.; Bergendahl G.; MacArthur R. B.; VanderWerff A.; Ashikaga T.; Bond J. P.; Ferguson W.; Roberts W.; Wada R. K.; et al. A Phase I Trial of DFMO Targeting Polyamine Addiction in Patients with Relapsed/Refractory Neuroblastoma. PLoS One 2015, 10 (5), e0127246 10.1371/journal.pone.0127246. 26018967 PMC4446210

[ref29] Simoneau A. R.; Gerner E. W.; Nagle R.; Ziogas A.; Fujikawa-Brooks S.; Yerushalmi H.; Ahlering T. E.; Lieberman R.; McLaren C. E.; Anton-Culver H.; et al. The effect of difluoromethylornithine on decreasing prostate size and polyamines in men: results of a year-long phase IIb randomized placebo-controlled chemoprevention trial. Cancer Epidemiol., Biomarkers Prev. 2008, 17 (2), 292–299. 10.1158/1055-9965.EPI-07-0658. 18268112 PMC2515594

[ref30] Meyskens F. L. Jr; Simoneau A. R.; Gerner E. W. Chemoprevention of prostate cancer with the polyamine synthesis inhibitor difluoromethylornithine. Recent Results Cancer Res. 2014, 202, 115–120. 10.1007/978-3-642-45195-9_14. 24531785

[ref31] Hogarty M. D.; Ziegler D. S.; Franson A.; Chi Y. Y.; Tsao-Wei D.; Liu K.; Vemu R.; Gerner E. W.; Bruckheimer E.; Shamirian A.; et al. Phase 1 study of high-dose DFMO, celecoxib, cyclophosphamide and topotecan for patients with relapsed neuroblastoma: a New Approaches to Neuroblastoma Therapy trial. Br. J. Cancer 2024, 130, 788 10.1038/s41416-023-02525-2. 38200233 PMC10912730

[ref32] Rajasekaran S.; Bupp C. P.; Leimanis-Laurens M.; Shukla A.; Russell C.; Junewick J.; Gleason E.; VanSickle E. A.; Edgerly Y.; Wittmann B. M.; et al. Repurposing eflornithine to treat a patient with a rare ODC1 gain-of-function variant disease. Elife 2021, 10, e67097 10.7554/eLife.67097. 34282722 PMC8291972

[ref33] Bachmann A. S.; VanSickle E. A.; Michael J.; Vipond M.; Bupp C. P. Bachmann-Bupp syndrome and treatment. Dev Med. Child Neurol 2024, 66 (4), 445–455. 10.1111/dmcn.15687. 37469105 PMC10796844

[ref34] Sims E. K.; Kulkarni A.; Hull A.; Woerner S. E.; Cabrera S.; Mastrandrea L. D.; Hammoud B.; Sarkar S.; Nakayasu E. S.; Mastracci T. L.; et al. Inhibition of polyamine biosynthesis preserves beta cell function in type 1 diabetes. Cell Rep. Med. 2023, 4 (11), 101261 10.1016/j.xcrm.2023.101261. 37918404 PMC10694631

[ref35] Chai X.; Zhan J.; Pan J.; He M.; Li B.; Wang J.; Ma H.; Wang Y.; Liu S. The rational discovery of multipurpose inhibitors of the ornithine decarboxylase. FASEB J. 2020, 34 (9), 10907–12921. 10.1096/fj.202001222R. 32767470

[ref36] Kim D. J.; Roh E.; Lee M. H.; Oi N.; Lim D. Y.; Kim M. O.; Cho Y. Y.; Pugliese A.; Shim J. H.; Chen H.; et al. Herbacetin Is a Novel Allosteric Inhibitor of Ornithine Decarboxylase with Antitumor Activity. Cancer Res. 2016, 76 (5), 1146–1157. 10.1158/0008-5472.CAN-15-0442. 26676750 PMC4775427

[ref37] Sahu P. N.; Sen A. Preventing Cancer by Inhibiting Ornithine Decarboxylase: A Comparative Perspective on Synthetic vs. Natural Drugs. Chem. Biodivers 2024, 21 (4), e202302067 10.1002/cbdv.202302067. 38404009

[ref38] Smithson D. C.; Lee J.; Shelat A. A.; Phillips M. A.; Guy R. K. Discovery of potent and selective inhibitors of Trypanosoma brucei ornithine decarboxylase. J. Biol. Chem. 2010, 285 (22), 16771–16781. 10.1074/jbc.M109.081588. 20220141 PMC2878083

[ref39] Wu F.; Grossenbacher D.; Gehring H. New transition state-based inhibitor for human ornithine decarboxylase inhibits growth of tumor cells. Mol. Cancer Ther. 2007, 6 (6), 1831–1839. 10.1158/1535-7163.MCT-07-0045. 17575112

[ref40] Fujita K.; Murakami Y.; Hayashi S. A macromolecular inhibitor of the antizyme to ornithine decarboxylase. Biochem. J. 1982, 204 (3), 647–652. 10.1042/bj2040647. 7126159 PMC1158403

[ref41] Almrud J. J.; Oliveira M. A.; Kern A. D.; Grishin N. V.; Phillips M. A.; Hackert M. L. Crystal structure of human ornithine decarboxylase at 2.1 A resolution: structural insights to antizyme binding. J. Mol. Biol. 2000, 295 (1), 7–16. 10.1006/jmbi.1999.3331. 10623504

[ref42] Bordwell F. G. Equilibrium Acidities in Dimethyl Sulfoxide Solution. Acc. Chem. Res. 1988, 21, 456–463. 10.1021/ar00156a004.

[ref43] Jackson L. K.; Brooks H. B.; Osterman A. L.; Goldsmith E. J.; Phillips M. A. Altering the reaction specificity of eukaryotic ornithine decarboxylase. Biochemistry 2000, 39 (37), 11247–11257. 10.1021/bi001209s. 10985770

[ref44] Dufe V. T.; Ingner D.; Heby O.; Khomutov A. R.; Persson L.; Al-Karadaghi S. A structural insight into the inhibition of human and Leishmania donovani ornithine decarboxylases by 1-amino-oxy-3-aminopropane. Biochem. J. 2007, 405 (2), 261–268. 10.1042/BJ20070188. 17407445 PMC1904517

[ref45] Grishin N. V.; Osterman A. L.; Brooks H. B.; Phillips M. A.; Goldsmith E. J. X-ray structure of ornithine decarboxylase from Trypanosoma brucei: the native structure and the structure in complex with alpha-difluoromethylornithine. Biochemistry 1999, 38 (46), 15174–15184. 10.1021/bi9915115. 10563800

[ref46] Khomutov R. M.; Hyvonen T.; Karvonen E.; Kauppinen L.; Paalanen T.; Paulin L.; Eloranta T.; Pajula R. L.; Andersson L. C.; Poso H. 1-Aminooxy-3-aminopropane, a new and potent inhibitor of polyamine biosynthesis that inhibits ornithine decarboxylase, adenosylmethionine decarboxylase and spermidine synthase. Biochem. Biophys. Res. Commun. 1985, 130 (2), 596–602. 10.1016/0006-291X(85)90458-9. 3861182

[ref47] Poulin R.; Secrist J. A.; Pegg A. E. Effect of 1-amino-oxy-3-aminopropane on polyamine metabolism and growth of L1210 cells. Biochem. J. 1989, 263 (1), 215–221. 10.1042/bj2630215. 2513802 PMC1133411

[ref48] Persson L.; Khomutov A. R.; Khomutov R. M. Feedback regulation of S-adenosylmethionine decarboxylase synthesis. Biochem. J. 1989, 257 (3), 929–931. 10.1042/bj2570929. 2930498 PMC1135678

[ref49] Khomutov R. M.; Denisova G. F.; Khomutov A. R.; Belostotskaia K. M.; Shlosman R. B. Aminooxypropylamine--an effective inhibitor of ornithine decarboxylase in vitro and in vivo. Bioorg. Khim. 1985, 11 (11), 1574–1576. 3841480

[ref50] Stanek J.; Frei J.; Mett H.; Schneider P.; Regenass U. 2-substituted 3-(aminooxy)propanamines as inhibitors of ornithine decarboxylase: synthesis and biological activity. J. Med. Chem. 1992, 35 (8), 1339–1344. 10.1021/jm00086a003. 1573631

[ref51] Mett H.; Stanek J.; Lopez-Ballester J. A.; Janne J.; Alhonen L.; Sinervirta R.; Frei J.; Regenass U. Pharmacological properties of the ornithine decarboxylase inhibitor 3-aminooxy-1-propanamine and several structural analogues. Cancer Chemother. Pharmacol. 1993, 32 (1), 39–45. 10.1007/BF00685874. 8462122

[ref52] Lide D. R. Handbook of Chemistry and Physics; CRC Press LLC: Boca Raton, FL, 2000.

[ref53] Zhou X. E.; Suino-Powell K.; Schultz C. R.; Aleiwi B.; Brunzelle J. S.; Lamp J.; Vega I. E.; Ellsworth E.; Bachmann A. S.; Melcher K. Structural basis of binding and inhibition of ornithine decarboxylase by 1-amino-oxy-3-aminopropane. Biochem. J. 2021, 478 (23), 4137–4149. 10.1042/BCJ20210647. 34796899 PMC9125680

[ref54] Leeson P. D.; Bento A. P.; Gaulton A.; Hersey A.; Manners E. J.; Radoux C. J.; Leach A. R. Target-Based Evaluation of ″Drug-Like″ Properties and Ligand Efficiencies. J. Med. Chem. 2021, 64 (11), 7210–7230. 10.1021/acs.jmedchem.1c00416. 33983732 PMC7610969

[ref55] Lipinski C. A.; Lombardo F.; Dominy B. W.; Feeney P. J. Experimental and computational approaches to estimate solubility and permeability in drug discovery and development settings. Adv. Drug Delivery Rev. 2001, 46 (1–3), 3–26. 10.1016/S0169-409X(00)00129-0. 11259830

[ref56] Das Gupta R.; Krause-Ihle T.; Bergmann B.; Müller I. B.; Khomutov A. R.; Müller S.; Walter R. D.; Lüersen K. 3-Aminooxy-1-aminopropane and derivatives have an antiproliferative effect on cultured Plasmodium falciparum by decreasing intracellular polyamine concentrations. Antimicrob. Agents Chemother. 2005, 49 (7), 2857–2864. 10.1128/AAC.49.7.2857-2864.2005. 15980361 PMC1168667

[ref57] Lipinski C. A. Lead- and drug-like compounds: the rule-of-five revolution. Drug Discov Today Technol. 2004, 1 (4), 337–341. 10.1016/j.ddtec.2004.11.007. 24981612

[ref58] Tinworth C. P.; Young R. J. Facts, Patterns, and Principles in Drug Discovery: Appraising the Rule of 5 with Measured Physicochemical Data. J. Med. Chem. 2020, 63 (18), 10091–10108. 10.1021/acs.jmedchem.9b01596. 32324397

[ref59] Porter R. K. Mammalian mitochondrial inner membrane cationic and neutral amino acid carriers. Biochim. Biophys. Acta 2000, 1459 (2–3), 356–362. 10.1016/S0005-2728(00)00172-9. 11004451

[ref60] Mtewa A. G.; Ngwira K.; Lampiao F.; Weisheit A.; Tolo C. A.; Ogwang P. E.; Sesaazi D. C. Fundamental Methods in Drug Permeability, pKa, LogP and LogDx Determination. J. Drug Res. Dev 2019, 5 (1), 1–6. 10.16966/2470-1009.146.

[ref61] Bennion B. J.; Be N. A.; McNerney M. W.; Lao V.; Carlson E. M.; Valdez C. A.; Malfatti M. A.; Enright H. A.; Nguyen T. H.; Lightstone F. C.; et al. Predicting a Drug’s Membrane Permeability: A Computational Model Validated With in Vitro Permeability Assay Data. J. Phys. Chem. B 2017, 121 (20), 5228–5237. 10.1021/acs.jpcb.7b02914. 28453293

[ref62] Refsgaard H. H.; Jensen B. F.; Brockhoff P. B.; Padkjaer S. B.; Guldbrandt M.; Christensen M. S. In silico prediction of membrane permeability from calculated molecular parameters. J. Med. Chem. 2005, 48 (3), 805–811. 10.1021/jm049661n. 15689164

[ref63] Lu D.; Chambers P.; Wipf P.; Xie X. Q.; Englert D.; Weber S. Lipophilicity screening of novel drug-like compounds and comparison to clog P. J. Chromatogr A 2012, 1258, 161–167. 10.1016/j.chroma.2012.07.078. 22939208 PMC3496770

[ref64] Benet L. Z.; Hosey C. M.; Ursu O.; Oprea T. I. BDDCS, the Rule of 5 and drugability. Adv. Drug Delivery Rev. 2016, 101, 89–98. 10.1016/j.addr.2016.05.007. PMC4910824 27182629

[ref65] Kuntz I. D.; Chen K.; Sharp K. A.; Kollman P. A. The maximal affinity of ligands. Proc. Natl. Acad. Sci. U.S.A. 1999, 96 (18), 9997–10002. 10.1073/pnas.96.18.9997. 10468550 PMC17830

[ref66] Keseru G. M.; Makara G. M. The influence of lead discovery strategies on the properties of drug candidates. Nat. Rev. Drug Discovery 2009, 8 (3), 203–212. 10.1038/nrd2796. 19247303

[ref67] Hopkins A. L.; Keseru G. M.; Leeson P. D.; Rees D. C.; Reynolds C. H. The role of ligand efficiency metrics in drug discovery. Nat. Rev. Drug Discovery 2014, 13 (2), 105–121. 10.1038/nrd4163. 24481311

[ref68] Hyvonen T.; Khomutov A. R.; Khomutov R. M.; Lapinjoki S.; Eloranta T. O. Uptake of 3H-labeled 1-aminooxy-3-aminopropane by baby hamster kidney cells. J. Biochem. 1990, 107 (6), 817–820. 10.1093/oxfordjournals.jbchem.a123131. 2118137

[ref69] Goodwin J. T.; Conradi R. A.; Ho N. F.; Burton P. S. Physicochemical determinants of passive membrane permeability: role of solute hydrogen-bonding potential and volume. J. Med. Chem. 2001, 44 (22), 3721–3729. 10.1021/jm010253i. 11606137

[ref70] Levin V. A.; Dolginow D.; Landahl H. D.; Yorke C.; Csejtey J. Relationship of octanol/water partition coefficient and molecular weight to cellular permeability and partitioning in s49 lymphoma cells. Pharm. Res. 1984, 1 (6), 259–266. 10.1023/A:1016393902123. 24277359

[ref71] Ghose A. K.; Viswanadhan V. N.; Wendoloski J. J. A knowledge-based approach in designing combinatorial or medicinal chemistry libraries for drug discovery. 1. A qualitative and quantitative characterization of known drug databases. J. Comb. Chem. 1999, 1 (1), 55–68. 10.1021/cc9800071. 10746014

[ref72] Li X.; Wilmanns M.; Thornton J.; Kohn M. Elucidating human phosphatase-substrate networks. Sci. Signal 2013, 6 (275), rs10 10.1126/scisignal.2003203. 23674824

[ref73] Fang Z.; Song Y.; Zhan P.; Zhang Q.; Liu X. Conformational restriction: an effective tactic in ’follow-on’-based drug discovery. Future Med. Chem. 2014, 6 (8), 885–901. 10.4155/fmc.14.50. 24962281

[ref74] Mann A.; Le Chatelier H.-L. In The Practice of Medicinal Chemistry, Wermuth C.-G., Ed.; Academic Press, 2003; pp 233–250.

[ref75] de Sena M Pinheiro P.; Rodrigues D. A.; do Couto Maia R.; Thota S.; Fraga C. A. M. The Use of Conformational Restriction in Medicinal Chemistry. Curr. Top. Med. Chem. 2019, 19 (19), 1712–1733. 10.2174/1568026619666190712205025. 31659944

[ref76] Corbeil C. R.; Williams C. I.; Labute P. Variability in docking success rates due to dataset preparation. J. Comput. Aided Mol. Des 2012, 26 (6), 775–786. 10.1007/s10822-012-9570-1. 22566074 PMC3397132

[ref77] Hornak V.; Abel R.; Okur A.; Strockbine B.; Roitberg A.; Simmerling C. Comparison of multiple Amber force fields and development of improved protein backbone parameters. Proteins 2006, 65 (3), 712–725. 10.1002/prot.21123. 16981200 PMC4805110

[ref78] Wang J.; Wolf R. M.; Caldwell J. W.; Kollman P. A.; Case D. A. Development and testing of a general amber force field. J. Comput. Chem. 2004, 25 (9), 1157–1174. 10.1002/jcc.20035. 15116359

[ref79] Onufriev A.; Bashford D.; Case D. A. Exploring protein native states and large-scale conformational changes with a modified generalized born model. Proteins 2004, 55 (2), 383–394. 10.1002/prot.20033. 15048829

[ref80] Pegg A. E.; Feith D. J. Polyamines and neoplastic growth. Biochem. Soc. Trans. 2007, 35 (2), 295–299. 10.1042/BST0350295. 17371264

[ref81] Hughes D. L.; Reamer R. A. The Effect of Acid Strength on the Mitsunobu Esterification Reaction: Carboxyl vs Hydroxyl Reactivity. J. Org. Chem. 1996, 61 (9), 2967–2971. 10.1021/jo952180e. 11667155

[ref82] Fjelbye K.; Marigo M.; Clausen R. P.; Juhl K. Enantioselective Fluorination of Spirocyclic β-Prolinals Using Enamine Catalysis. Synlett 2017, 28, 425–428. 10.1055/s-0036-1588100.

[ref83] Koomoa D. L.; Yco L. P.; Borsics T.; Wallick C. J.; Bachmann A. S. Ornithine Decarboxylase Inhibition by alpha-Difluoromethylornithine Activates Opposing Signaling Pathways via Phosphorylation of Both Akt/Protein Kinase B and p27Kip1 in Neuroblastoma. Cancer Res. 2008, 68 (23), 9825–9831. 10.1158/0008-5472.CAN-08-1865. 19047162 PMC2596629

[ref84] Evageliou N. F.; Haber M.; Vu A.; Laetsch T. W.; Murray J.; Gamble L. D.; Cheng N. C.; Liu K.; Reese M.; Corrigan K. A.; et al. Polyamine Antagonist Therapies Inhibit Neuroblastoma Initiation and Progression. Clin. Cancer Res. 2016, 22 (17), 4391–4404. 10.1158/1078-0432.CCR-15-2539. 27012811

[ref85] Hogarty M. D.; Norris M. D.; Davis K.; Liu X.; Evageliou N. F.; Hayes C. S.; Pawel B.; Guo R.; Zhao H.; Sekyere E.; et al. ODC1 Is a Critical Determinant of MYCN Oncogenesis and a Therapeutic Target in Neuroblastoma. Cancer Res. 2008, 68 (23), 9735–9745. 10.1158/0008-5472.CAN-07-6866. 19047152 PMC2596661

[ref86] Rounbehler R. J.; Li W.; Hall M. A.; Yang C.; Fallahi M.; Cleveland J. L. Targeting ornithine decarboxylase impairs development of MYCN-amplified neuroblastoma. Cancer Res. 2009, 69 (2), 547–553. 10.1158/0008-5472.CAN-08-2968. 19147568 PMC2749594

[ref87] Boike L.; Henning N. J.; Nomura D. K. Advances in covalent drug discovery. Nat. Rev. Drug Discovery 2022, 21 (12), 881–898. 10.1038/s41573-022-00542-z. 36008483 PMC9403961

[ref88] Moinard C.; Cynober L.; de Bandt J. P. Polyamines: metabolism and implications in human diseases. Clin Nutr 2005, 24 (2), 184–197. 10.1016/j.clnu.2004.11.001. 15784477

[ref89] Zahedi K.; Barone S.; Soleimani M. Polyamines and Their Metabolism: From the Maintenance of Physiological Homeostasis to the Mediation of Disease. Med. Sci. 2022, 10 (3), 38 10.3390/medsci10030038. PMC9326668 35893120

[ref90] Casero R. A.; Pegg A. E. Polyamine catabolism and disease. Biochem. J. 2009, 421 (3), 323–338. 10.1042/BJ20090598. 19589128 PMC2756025

[ref91] El-Sayed A. S.; Shindia A. A. PLP-dependent enzymes: A potent therapeutic approach for cancer and cardiovascular diseases. In Targets in Gene Therapy; You Y., Ed.,; IntechOpen, 2011.

[ref92] Afrin A.; Afshan T. S.; VanSickle E. A.; Michael J.; Laarman R. L.; Bupp C. P. Improvement of dermatological symptoms in patients with Bachmann-Bupp syndrome using difluoromethylornithine treatment. Pediatr. Dermatol. 2023, 40, 528 10.1111/pde.15187. 36443247

[ref93] Stewart T. M.; Foley J. R.; Holbert C. E.; Khomutov M.; Rastkari N.; Tao X.; Khomutov A. R.; Zhai R. G.; Casero R. A. Jr Difluoromethylornithine rebalances aberrant polyamine ratios in Snyder-Robinson syndrome. EMBO Mol. Med. 2023, 15 (11), e17833 10.15252/emmm.202317833. 37702369 PMC10630878

[ref94] Mounce B. C.; Cesaro T.; Moratorio G.; Hooikaas P. J.; Yakovleva A.; Werneke S. W.; Smith E. C.; Poirier E. Z.; Simon-Loriere E.; Prot M.; et al. Inhibition of Polyamine Biosynthesis Is a Broad-Spectrum Strategy against RNA Viruses. J. Virol. 2016, 90 (21), 9683–9692. 10.1128/JVI.01347-16. 27535047 PMC5068521

[ref95] Olsen M. E.; Cressey T. N.; Muhlberger E.; Connor J. H. Differential Mechanisms for the Involvement of Polyamines and Hypusinated eIF5A in Ebola Virus Gene Expression. J. Virol. 2018, 92 (20), e01260 10.1128/JVI.01260-18. 30045993 PMC6158423

[ref96] Huang M.; Zhang W.; Chen H.; Zeng J. Targeting Polyamine Metabolism for Control of Human Viral Diseases. Infect Drug Resist 2020, 13, 4335–4346. 10.2147/IDR.S262024. 33293837 PMC7718961

[ref97] Jimenez Gutierrez G. E.; Borbolla Jimenez F. V.; Munoz L. G.; Tapia Guerrero Y. S.; Murillo Melo N. M.; Cristobal-Luna J. M.; Leyva Garcia N.; Cordero-Martinez J.; Magana J. J. The Molecular Role of Polyamines in Age-Related Diseases: An Update. Int. J. Mol. Sci. 2023, 24 (22), 16469 10.3390/ijms242216469. 38003659 PMC10671757

[ref98] Klee N.; Wong P. E.; Baragana B.; Mazouni F. E.; Phillips M. A.; Barrett M. P.; Gilbert I. H. Selective delivery of 2-hydroxy APA to Trypanosoma brucei using the melamine motif. Bioorg. Med. Chem. Lett. 2010, 20 (15), 4364–4366. 10.1016/j.bmcl.2010.06.070. 20615694 PMC2935964

[ref99] Zhou X. E.; Schultz C. R.; Suino Powell K.; Henrickson A.; Lamp J.; Brunzelle J. S.; Demeler B.; Vega I. E.; Bachmann A. S.; Melcher K. Structure and Enzymatic Activity of an Intellectual Disability-Associated Ornithine Decarboxylase Variant, G84R. ACS Omega 2022, 7 (38), 34665–34675. 10.1021/acsomega.2c04702. 36188294 PMC9520691

