# Supplementary material for: Design, Synthesis, and Biological Activity of Novel Ornithine Decarboxylase (ODC) Inhibitors
Source: J Med Chem. 2025 Mar 4;68(5):5760–73. doi: 10.1021/acs.jmedchem.4c03120 (PMC11912471; doi:10.1021/acs.jmedchem.4c03120)

Supporting Information

Design, Synthesis, and Biological Activity of Novel Ornithine Decarboxylase (ODC) Inhibitors

Chad R. Schultz[1,2,+], Bilal Aleiwi[3,+], X. Edward Zhou[4], Kelly Suino-Powell[4], Karsten Melcher[4], Nuno M. S. Almeida[5], Angela K. Wilson[5], Edmund L. Ellsworth[3,*], André S. Bachmann[1,2,*]

[1]Department of Pediatrics and Human Development, College of Human Medicine, Michigan State University, Grand Rapids, MI 49503, USA; [2]International Center for Polyamine Disorders, Grand Rapids, MI 49503, USA; [3]Department of Pharmacology and Toxicology, College of Human Medicine, East Lansing, MI 48824, USA; [4]Department of Structural Biology, Van Andel Institute, Grand Rapids, MI 49503, USA; [5]Department of Chemistry, Michigan State University, East Lansing, MI 48824, USA.

[+]Equal Contribution
*Corresponding Authors: André S. Bachmann, Department of Pediatrics and Human Development, College of Human Medicine, Michigan State University, 400 Monroe Ave, NW, Grand Rapids, MI 49503, USA. Tel: +616-234-2841, E-mail: bachma26@msu.edu and Edmund L. Ellsworth, Department of Pharmacology and Toxicology, College of Human Medicine, Michigan State University, 1355 Bogue Street, East Lansing, MI 48824, USA. Tel: +517-884-8816, E-mail: ellswo59@msu.edu

Table of Contents

Item	Page
**ODC Protein Expression and Purification**	S2
**ODC Protein Crystallization**	S2
**Crystal Data Collection, Structure Determination and Analysis**	S2-S3
**Analog Syntheses**<br>a. 3-(aminooxy)propan-1-amine dihydrochloride (**APA**)<br>b. ({4-[(E)-[(3-Aminopropoxy)imino]methyl]-5-hydroxy-6-methylpyridin-3-yl}methoxy)phosphonic acid (**APA-PLP**)<br>c. 3-[(aminooxy)methyl]azetidine dihydrochloride (**5**)<br>d. 2-[2-(aminooxy)ethyl]piperidine dihydrochloride (**6**)<br>e. 3-R-[(aminooxy)methyl]piperidine dihydrochloride (**7-*R***)<br>f. 3-S-[(aminooxy)methyl]piperidine dihydrochloride (**7-*S***)<br>g. 2-[(aminooxy)methyl] morpholine dihydrochloride (**8**)<br>h. 4-(aminooxy)piperidine dihydrochloride (**9**)<br>i. 3-[(aminooxy)methyl]pyrrolidine dihydrochloride (**10**)<br>j. (3R)-3-[(aminooxy)methyl]pyrrolidine dihydrochloride (**10-*R***)<br>k. (3S)-3-[(aminooxy)methyl]pyrrolidine dihydrochloride (**10-*S***)<br>l. ({5-hydroxy-6-methyl-4-[(E)-({[(3R)-pyrrolidin-3-yl]methoxy}imino)methyl]pyridin-3-yl}methoxy)phosphonic acid (**10-*R*-PLP**)	S4-S14

m. ({5-hydroxy-6-methyl-4-[(E)-({[(3S)-pyrrolidin-3-yl]methoxy}imino)methyl]pyridin-3-yl}methoxy)phosphonic acid (**10-*S*-PLP**)<br>n. 3-[(aminooxy)methyl]-3-fluoropyrrolidine di-formic acid. (**11**)<br>o. ({4-[(E)-{[(3-fluoropyrrolidin-3-yl)methoxy]imino}methyl]-5-hydroxy-6-methylpyridin-3-yl}methoxy)phosphonic acid (**11-PLP**)	
**Computational Modeling Validation**	S14
**References**	S15
**Appendix 1.** ^{1}H and ^{13}C NMR spectra of compounds **APA-PLP**, **5**, **6**, 7, **7-*R***, **7-*S***, **8**, **9**, **10**, **10-*R***, **10-*S***, **10-*S*-PLP**, ***10-R-PLP***, **11, 11-PLP.**	S16-S47
**Appendix 2.** HPLC chromatograms for compounds **10-*R*-PLP**, ***10-S-PLP***, **11-PLP, APA-PLP.**	S48-S57
**Appendix 3.** HRMS spectra of compounds **APA-PLP**, **5**, **6**, 7, **7-*R***, **7-*S***, **8**, **9**, **10**, **10-*R***, **10-*S***, **10-*S*-PLP**, ***10-R-PLP***, **11, 11-PLP.**	S58-S72

1) ODC Protein Expression and Purification

The full-length human ornithine decarboxylase 1 (*ODC1*) gene and ODC1(1-423) were expressed in pET28 (Novagen) with a C-terminal His6 tag. *E. coli* BL21(DE3) cells transformed with this expression plasmid were grown in LB broth at 16°C to an OD600 of 1 and induced with 0.1 mM IPTG. The next morning, cells were harvested, resuspended in 150 mL buffer A (20 mM Tris pH8, 200 mM NaCl, 10% glycerol) per 2 liters of cells, and passed three times through a French Press with pressure set at 1000 Pa. The lysate was centrifuged at 34,571 x g for 1 hour, and the supernatant was loaded on a 5 mL HisTrap FF column (GE Healthcare). The column was washed with 100 mL buffer A + 25 mM imidazole and eluted with 50 mL of Buffer B (20 mM Tris pH8, 500 mM imidazole, 10% glycerol). The peak fractions were further purified by passing through at HiLoad 26/200 Superdex 200pg (GE Healthcare) in 20 mM Tris pH 8.0, 200 mM NaCl, 2 mM DTT, 1 mM EDTA, 10% glycerol. The eluted protein was complexed with pyridoxal 5′-phosphate monohydrate (PLP) (Sigma-Aldrich) at a 5-fold molar excess, and an ODC1 inhibitor at a 10-fold molar excess for 1 h at 4 °C. The complexes were filter-concentrated to 16 mg/mL.

2) ODC Protein Crystallization

The ODC-PLP-10R complex crystals were grown in sitting drop wells with 0.2 µL of protein and 0.2 µL of well solution containing 25% w/v polyethylene glycol 3350, 0.2 M magnesium chloride hexahydrate, 0.1 M BIS-TRIS pH 5.5.
The ODC1-PLP-10S complex crystals were grown in sitting drop wells with 0.2 µL of protein and 0.2 µL of well solution containing 0.1 M sodium citrate tribasic dihydrate pH 5.5, 22% w/v polyethylene glycol 3350, 0.10% w/v n-octyl-b-D-glucoside.

3) Crystal Data Collection, Structure Determination and Analysis

The crystals of ODC1/PLP/10-R and ODC1/PLP/10-S formed in the P212121 space group. The datasets were collected with an EIGER 16M pixel array detector at the ID line of sector 21 of the Advanced Photon Source at Argonne National Laboratory (Argonne, IL).
The data of ODC1/PLP/10-R were indexed to 2.9 Å and the data of ODC1/PLP/10-S were indexed to 2.0 Å with MOSFLM [1] and scaled with AIMLESS in the ccp4 package (http://www.ccp4.ac.uk). The CCP4 program PHASER was used for molecular replacement, with the crystal structure of 2OO0 [2] as a search model. The initial model was manually built in COOT [3] and refined with the PHENIX program package [4]. Figures were prepared using PyMOL (DeLano Scientific, San Carlos, CA, http://www.pymol.org) (Table 1).

Table 1. Crystallography data statistics and structure refinement

	ODC1/PLP/10-R	ODC1/PLP/10-S
**Data collection**		
Space group	$P2_12_12_1$	$P2_12_12_1$
Cell dimensions		
a, *b*, *c* (Å)	74.6, 86.4, 153.2	74.6, 86.9, 154.0
α, β, γ (°)	90, 90, 90	90, 90, 90
Resolution (Å)	31.7-2.90 (2.98-2.90) *	44.4-1.99 (2.04-1.99)
R_{sym} or R_{merge}	0.31 (1.80)	0.09 (0.73)
$I / \sigma I$	7.9 (1.7)	10.6 (1.7)
CC1/2	0.994 (0.58)	0.997 (0.60)
Completeness (%)	99.8 (100)	99.3 (99.3)
Redundancy	12.8 (13.5)	5.2 (3.7)
**Refinement**		
Resolution (Å)	31.7-2.90 (3.03-2.90)	35-1.99 (2.02-1.99)
No. reflections	22546 (2647)	70204 (3418)
R_{work} / R_{free}	19.3/22.4 (31.3/36.5)	19.0/21.2 (32.5/35.1)
No. residues/atoms		
Protein	814/6384	821/6425
Ligand	2/39	2/39
Water	13	489
B-factors		
Protein	72.1	33.3
Ligand	71.0	33.3
Water	66.2	41.1
R.m.s. deviations		
Bond lengths (Å)	0.005	0.007
Bond angles (°)	0.976	1.137
Ramachandran plot (%)		
Favored	98.62	98.15
Outliers	0.0	0.0
Clash score	3.94	3.36
Molprobity score	1.18	1.13

*Values in parentheses are for highest-resolution shell.

4) ODC Inhibitor Syntheses

Materials and Methods: All commercial reagents and solvents were used as received. Anhydrous solvents were dried over 4 A° molecular sieves. TLC was performed on silica gel 60G F_{254} glass plates. ^{1}H NMR spectra were recorded on an Agilent VXR (500 MHz). Purity for final compounds is greater than 95% by NMR. Purity of PLP analogues was measured using Agilent 1200 series high performance liquid chromatography (HPLC) systems with UV detection at 214 nm and using a Delta-Pak 300 x 3.9 mm C18 15 µm, 300A° column with isocratic elution at 15% methanol in water. High resolution mass spectra were recorded with a Waters G2-XS-QTof using an electrospray ionization mode (ESI). Optical rotations were measured on a Perkin Elmer polarimeter (concentration (c) is given as g/ mL).

3-(aminooxy)propan-1-amine dihydrochloride APA.

***tert*-Butyl {3-[(1,3-dioxo-1,3-dihydro-2*H*-isoindol-2-yl)oxy]propyl}carbamate (4).** A mixture of 3-(tert-butoxycarbonylamino)-1-propanol (**12a**) (1.0 g, 5.71 mmol), *N*-hydroxyphthalimide (1.03 g, 6.28 mmol) and triphenylphosphine (1.60 g, 6.28 mmol) in dry tetrahydrofuran (20 mL), under an argon atmosphere, was stirred at 0 ^{0}C for 20 minutes. A solution of diisopropyl azodicarboxylate (1.30 g, 6.28 mmol) in tetrahydrofuran (10 mL) was then added dropwise and allowed to slowly warm to room temperature (2 hours). Upon completion, the reaction was concentrated *in vacuo*. The resulting residue was purified *via* medium pressure liquid chromatography (SiO_2, 100% hexanes to 60% ethyl acetate / hexanes (1.54 g, 84% yield). ^{1}H NMR (500 MHz, Chloroform-*d*) δ 7.84 (dd, *J* = 5.4, 3.1 Hz, 2H), 7.76 (dd, *J* = 5.5, 3.1 Hz, 2H), 4.27 (t, *J* = 5.9 Hz, 2H), 3.42 (q, *J* = 6.4 Hz, 2H), 1.95 (q, *J* = 6.1 Hz, 2H), 1.44 (s, 9H).

3-(aminooxy)propan-1-amine dihydrochloride (APA). To a solution of *tert*-butyl {3-[(1,3-dioxo-1,3-dihydro-2*H*-isoindol-2-yl)oxy]propyl}carbamate (**4**) (1.0 g, 3.13 mmol) in dry dichloromethane (15 mL), under an argon atmosphere was added hydrazine monohydrate (0.30 mL, 3.13 mmol) dropwise during 5 minutes. The solution turned into a suspension in 30 minutes. After 2 hours, the white precipitate was filtered off and washed with cold dichloromethane (10 mL). The filtrate was concentrated in vacuo and the resulting residue was used without further purification. The residue was taken up in methanol (5 mL) and treated dropwise with hydrochloric acid (4.0 N in dioxane, 4.0 mL, 16.0 mmol). The mixture was stirred at rt, under an argon atmosphere, for 2 hours and concentrated. The resulting residue was taken up in water (10 mL), washed with ethyl acetate (3 x 5 mL) and lyophilized to give the di-HCl salt of 3-(aminooxy)propan-1-amine (**APA**) as a fine white powder (0.40 g, 78% yield for two steps). ^{1}H NMR (500 MHz, D_2O) δ 4.03 (t, *J* = 5.9 Hz, 2H), 2.96 (t, *J* = 7.5 Hz, 2H), 1.91 (p, *J* = 6.4 Hz, 2H). ^{13}C NMR (126 MHz, D_2O) δ 72.32, 36.54, 25.14. HRMS (ESI) m/z calculated for $C_3H_{11}N_2O$ [M+H], 91.0871; found 91.0879.

({4-[(E)-[(3-Aminopropoxy)imino]methyl]-5-hydroxy-6-methylpyridin-3-yl}methoxy)phosphonic acid (APA-PLP). To a solution of 3-(aminooxy)propan-1-amine dihydrochloride **APA** (0.105 g, 0.65 mmol) in PBS buffer (4.0 mL, pH 7.40) was added pyridoxal 5′-phosphate **2** (0.16 g, 0.65 mmol). The resulting mixture was stirred at rt for 18 h. Subsequently, the reaction mixture was passed through a sintered glass filter. The filtrate was lyophilized to give

the title compound a semi-solid hygroscopic material (0.16 g, 78% yield) contaminated with ~3% ({4-[(Z)-[(3-aminopropoxy)imino]methyl]-5-hydroxy-6-methylpyridin-3-yl}methoxy)phosphonic acid. ^{1}H NMR (500 MHz, D_2O) δ 8.60 – 8.56 (m, 1H), 8.05 (d, *J* = 0.8 Hz, 1H), 4.96 (d, *J* = 7.7 Hz, 2H), 4.35 (t, *J* = 5.8 Hz, 2H), 3.02 (t, *J* = 7.4 Hz, 2H), 2.52 (s, 3H), 2.05 – 1.96 (m, 2H). ^{13}C NMR (126 MHz, D_2O) δ 152.78, 146.74, 144.72, 133.31, 133.25, 129.75, 127.31, 73.13, 61.68, 61.65, 36.86, 26.11, 14.24. HRMS (ESI) m/z calculated for $C_{11}H_{19}N_3O_6P$ [M+H], 320.1011; found 320.1030. Purity: 98.3% by HPLC. Melting point: 160 ^{0}C (Decomposition). HPLC purity >96.7%.

3-[(Aminooxy)methyl]azetidine dihydrochloride (5).

***tert*-butyl-3-{[(1,3-dioxo-1,3-dihydro-2*H*-isoindol-2-yl)oxy]methyl}azetidine-1-carboxylate (13b).** A mixture of *tert*-butyl 3-(hydroxymethyl)azetidine-1-carboxylate (**12b**) (2.0 g, 10.69 mmol), *N*-hydroxyphthalimide (1.84 g, 11.22 mmol) and triphenylphosphine (2.95 g, 11.22 mmol) in dry tetrahydrofuran (40 mL), under an argon atmosphere, was stirred at 0 ^{0}C for 20 minutes. A solution of diisopropyl azodicarboxylate (2.30 g, 6.28 mmol) in tetrahydrofuran (20 mL) was then added dropwise and allowed to slowly warm to room temperature (2 hours). Upon completion, the reaction was concentrated *in vacuo*. The resulting residue was purified *via* medium pressure liquid chromatography (SiO_2, 100% hexanes to 60% ethyl acetate / hexanes (1.54 g, 84% yield) to provide the title compound. ^{1}H NMR (500 MHz, Chloroform-d) *δ* 7.84 (dd, J = 5.4, 3.1 Hz, 2H), 7.83 – 7.73 (m, 2H), 4.35 (d, J = 7.2 Hz, 2H), 4.13 – 4.03 (m, 2H), 3.84 (m, 2H), 2.99 (m, 1H), 1.43 (s, 9H).

3-[(Aminooxy)methyl]azetidine dihydrochloride (5). To a solution of *tert*-butyl 3-{[(1,3-dioxo-1,3-dihydro-2*H*-isoindol-2-yl)oxy]methyl}azetidine-1-carboxylate **13b** (0.50 g, 1.50 mmol) in dry dichloromethane (5 mL), under an argon atmosphere was added hydrazine monohydrate (0.15 mL, 1.57 mmol) dropwise during 5 minutes. The solution turned into a suspension in 30 minutes. After 2 hours, the white precipitate was filtered off and washed with cold dichloromethane (5 mL). The filtrate was concentrated in vacuo and the resulting residue was used without further purification. The residue was taken up in methanol (5 mL) and treated dropwise with hydrochloric acid (4.0 N in dioxane, 2.0 mL, 8.0 mmol). The mixture was stirred at rt, under an argon atmosphere, for 2 hours and concentrated. The resulting residue was taken up in water (10 mL), washed with ethyl acetate (3 x 5 mL) and lyophilized to give 3-[(aminooxy)methyl]azetidine di-hydrochloride (**5**) as a semi-solid hygroscopic material (0.19 g, 70% yield for two steps). ^{1}H NMR (500 MHz, D_2O) *δ* 4.27 – 4.19 (m, 1H), 4.03 – 3.97 (m, 1H), 3.77 – 3.70 (m, 1H), 3.37 – 3.28 (m, 1H), 3.19 – 3.05 (m, 3H). ^{13}C NMR (126 MHz, D_2O) δ 73.22, 49.39, 39.50, 39.29. HRMS (ESI) m/z calculated for $C_4H_{11}N_2O$ [M+H], 103.0871; found 103.0891.

2-[2-(Aminooxy)ethyl]piperidine dihydrochloride (6).

***tert*-Butyl 2-(2-[1,3-dixoxo-isoindol-2-yl)oxy]ethyl)piperidine-1-carboxylate (13c).** A mixture of *tert*-butyl 2-(2-hydroxyethyl)piperidine-1-carboxylate **12c** (2.0 g, 8.73 mmol), N-hydroxyphthalimide (1.57 g, 9.60 mmol) and triphenylphosphine (2.52 g, 9.60 mmol) in dry tetrahydrofuran (50 mL), under an argon atmosphere, was stirred at 0 ^{0}C for 20 minutes. A solution of diisopropyl azodicarboxylate (1.95 g, 9.60 mmol) in tetrahydrofuran (20 mL) was then added dropwise and allowed to slowly warm to room temperature (2 hours). Upon completion, the reaction was concentrated *in vacuo*. The resulting residue was purified *via* medium pressure liquid

chromatography (SiO_2, 100% hexanes to 25% ethyl acetate / hexanes to provide the title compound (2.90 g, 88% yield). ^{1}H NMR (500 MHz, Chloroform-*d*) δ 7.85 (dd, J = 5.4, 3.1 Hz, 2H), 7.76 (dd, J = 5.4, 3.1 Hz, 2H), 4.46 (dq, J = 7.9, 4.3, 3.3 Hz, 1H), 4.27 (ddd, J = 9.5, 8.4, 6.5 Hz, 1H), 4.18 – 4.10 (m, 1H), 4.00 (m, 1H), 2.82 (t, J = 13.2 Hz, 1H), 2.30 – 2.16 (m, 1H), 1.93 (dt, J = 13.7, 7.3 Hz, 1H), 1.74 – 1.54 (m, 6H), 1.45 (s, 9H).

2-[2-(Aminooxy)ethyl]piperidine dihydrochloride (6). To a solution of *tert*-Butyl 2-(2-[1,3-dixoxo-isoindol-2-yl)oxy]ethyl)piperidine-1-carboxylate **13c** (2.50 g, 6.70 mmol) in dry dichloromethane (34 mL), under an argon atmosphere was added hydrazine monohydrate (0.60 mL, 6.98 mmol) dropwise during 5 minutes. The solution turned into a suspension in 30 minutes. After 2 hours, the white precipitate was filtered off and washed with cold dichloromethane (10 mL). The filtrate was concentrated in vacuo and the resulting residue was used without further purification. The residue was taken up in methanol (5 mL) and treated dropwise with hydrochloric acid (4.0 N in dioxane, 9.0 mL, 36.0 mmol). The mixture was stirred at rt, under an argon atmosphere, for 2 hours and concentrated. The resulting residue was taken up in water (10 mL), washed with ethyl acetate (3 x 5 mL) and lyophilized to give the di-HCl salt of 2-[2-(aminooxy)ethyl]piperidine (**6**) as a tan pasty solid (1.11 g, 76% yield for two steps). ^{1}H NMR (500 MHz, D_2O) δ 3.99 (td, J = 5.3, 1.8 Hz, 2H), 3.23 (dp, J = 12.8, 2.2 Hz, 1H), 3.14 – 3.05 (m, 1H), 2.81 (td, J = 12.8, 2.9 Hz, 1H), 1.91 – 1.75 (m, 2H), 1.71 (d, J = 16.6 Hz, 2H), 1.53 – 1.43 (m, 1H), 1.46 – 1.33 (m, 1H), 1.36 – 1.26 (m, 1H), 1.22 (s, 1H). ^{13}C NMR (126 MHz, D_2O) δ 71.20, 54.28, 44.86, 31.30, 27.91, 21.60, 21.28. HRMS (ESI) m/z calculated for $C_7H_{17}N_2O$ [M+H], 145.1341; found 145.1360. Melting point: 169 ^{0}C (Decomposition).

***tert*-Butyl 3-{[(1,3-dioxo-1,3-dihydro-2*H*-isoindol-2-yl)oxy]methyl}piperidine-1-carboxylate.** A mixture of tert-butyl 3-(hydroxymethyl)piperidine-1-carboxylate (2.0 g, 9.29 mmol), N-hydroxyphthalimide (1.70 g, 10.22 mmol) and triphenylphosphine (2.70 g, 10.22 mmol) in dry tetrahydrofuran (40 mL), under an argon atmosphere, was stirred at 0 0C for 20 minutes. A solution of diisopropyl azodicarboxylate (2.10 g, 10.22 mmol) in tetrahydrofuran (20 mL) was then added dropwise and allowed to slowly warm to room temperature (2 hours). Upon completion, the reaction was concentrated in vacuo. The resulting residue was purified via medium pressure liquid chromatography (SiO2, 100% hexanes to 60% ethyl acetate / hexanes (3.0 g, 89% yield) to provide the title compound. ^{1}H NMR (500 MHz, Chloroform-d) δ 7.83 (dd, J = 5.4, 3.1 Hz, 2H), 7.74 (dd, J = 5.5, 3.1 Hz, 2H), 4.06 (m, 2H), 3.87 (m, 1H), 2.93 – 2.78 (m, 2H), 2.08 – 1.93 (m, 1H), 1.70 (m, 1H), 1.53-1.34 (m, 4H), 1.46 (s, 9H).

3-[(Aminooxy)methyl]piperidine dihydrochloride (7). To a solution of tert-butyl 3-{[(1,3-dioxo-1,3-dihydro-2H-isoindol-2-yl)oxy]methyl}piperidine-1-carboxylate (3.0 g, 8.34 mmol) in dry dichloromethane (42 mL), under an argon atmosphere was added hydrazine monohydrate (0.70 mL, 8.75 mmol) dropwise during 5 minutes. The solution turned into a suspension in 30 minutes. After 2 hours, the white precipitate was filtered off and washed with cold dichloromethane (20 mL). The filtrate was concentrated in vacuo and the resulting residue was used without further purification. The residue was taken up in methanol (10 mL) and treated dropwise with hydrochloric acid (4.0 N in dioxane, 10.50 mL, 41.70 mmol). The mixture was stirred at rt, under an argon atmosphere, for 2 hours and concentrated. The resulting residue was taken up in water (20 mL), washed with ethyl acetate (3 x 10 mL) and lyophilized to give the di-HCl salt of 3-[(aminooxy)methyl]piperidine (7) as a fine white powder (1.29 g, 76% yield for two steps). ^{1}H

NMR (500 MHz, D_2O) δ 3.91 (dd, J = 8.5, 5.0 Hz, 1H), 3.83 (dd, J = 8.6, 7.1 Hz, 1H), 3.35 – 3.28 (m, 1H), 3.27 – 3.20 (m, 1H), 2.81 – 2.65 (m, 2H), 2.14 – 2.01 (m, 1H), 1.81 (dq, J = 13.8, 3.3 Hz, 1H), 1.74 (ddt, J = 15.3, 4.0, 2.0 Hz, 1H), 1.57 (dtt, J = 14.5, 12.9, 4.0 Hz, 1H), 1.23 (qd, J = 12.7, 3.9 Hz, 1H). ^{13}C NMR (126 MHz, D_2O) δ 76.03, 45.42, 43.98, 32.38, 23.93, 21.17. HRMS (ESI) m/z calculated for $C_6H_{15}N_2O$ [M+H], 131.1184; found 131.1189.

3-*R*-[(Aminooxy)methyl]piperidine dihydrochloride (7-*R*)

***tert*-Butyl 3-*R*-{[(1,3-dioxo-1,3-dihydro-2H-isoindol-2-yl)oxy]methyl}piperidine-1-carboxylate (13j).** A mixture of *tert*-butyl 3-*R*-(hydroxymethyl)piperidine-1-carboxylate **12j** (2.0 g, 9.29 mmol), N-hydroxyphthalimide (1.70 g, 10.22 mmol) and triphenylphosphine (2.70 g, 10.22 mmol) in dry tetrahydrofuran (40 mL), under an argon atmosphere, was stirred at 0 0C for 20 minutes. A solution of diisopropyl azodicarboxylate (2.10 g, 10.22 mmol) in tetrahydrofuran (20 mL) was then added dropwise and allowed to slowly warm to room temperature (2 hours). Upon completion, the reaction was concentrated in vacuo. The resulting residue was purified via medium pressure liquid chromatography (SiO_2, 100% hexanes to 60% ethyl acetate / hexanes to provide the title compound (2.9 g, 87% yield). ^{1}H NMR (500 MHz, Chloroform-d) δ 7.83 (dd, J = 5.4, 3.1 Hz, 2H), 7.74 (dd, J = 5.5, 3.1 Hz, 2H), 4.06 (m, 2H), 3.87 (m, 1H), 2.93 – 2.78 (m, 2H), 2.08 – 1.93 (m, 1H), 1.70 (m, 1H), 1.53-1.34 (m, 4H), 1.46 (s, 9H).

(3*R*)-3-[(Aminooxy)methyl]piperidine dihydrochloride (7-*R*). To a solution of *tert*-butyl 3-*R*-{[(1,3-dioxo-1,3-dihydro-2*H*-isoindol-2-yl)oxy]methyl}piperidine-1-carboxylate **13j** (3.0 g, 8.34 mmol) in dry dichloromethane (42 mL), under an argon atmosphere was added hydrazine monohydrate (0.70 mL, 8.75 mmol) dropwise during 5 minutes. The solution turned into a suspension in 30 minutes. After 2 hours, the white precipitate was filtered off and washed with cold dichloromethane (20 mL). The filtrate was concentrated *in vacuo* and the resulting residue was used without further purification. The residue was taken up in methanol (10 mL) and treated dropwise with hydrochloric acid (4.0 N in dioxane, 10.50 mL, 41.70 mmol). The mixture was stirred at rt, under an argon atmosphere, for 2 hours and concentrated. The resulting residue was taken up in water (20 mL), washed with ethyl acetate (3 x 10 mL) and lyophilized to give the di-HCl salt of 3-*R*-[(aminooxy)methyl]piperidine (**7-*R***) as a fine tan powder (1.29 g, 76% yield for two steps). ^{1}H NMR (500 MHz, D_2O) δ 3.91 (dd, J = 8.5, 5.0 Hz, 1H), 3.83 (dd, J = 8.6, 7.1 Hz, 1H), 3.35 – 3.28 (m, 1H), 3.27 – 3.20 (m, 1H), 2.81 – 2.65 (m, 2H), 2.14 – 2.01 (m, 1H), 1.81 (dq, J = 13.8, 3.3 Hz, 1H), 1.74 (ddt, J = 15.3, 4.0, 2.0 Hz, 1H), 1.57 (dtt, J = 14.5, 12.9, 4.0 Hz, 1H), 1.23 (qd, J = 12.7, 3.9 Hz, 1H). ^{13}C NMR (126 MHz, D_2O) δ 76.03, 45.42, 43.98, 32.38, 23.93, 21.17. HRMS (ESI) m/z calculated for $C_6H_{15}N_2O$ [M+H], 131.1184; found 131.1208. Melting point: 342 ^{0}C (Decomposition). $[\alpha]_D^{20}$ = -5.2 (*c* 0.01, H_2O).

(3S)-3-[(Aminooxy)methyl]piperidine dihydrochloride (7-*S*).

***tert*-Butyl 3-*S*-{[(1,3-dioxo-1,3-dihydro-2*H*-isoindol-2-yl)oxy]methyl}piperidine-1-carboxylate (13f).** A mixture of *tert*-butyl 3-*S*-(hydroxymethyl)piperidine-1-carboxylate **12f** (2.0 g, 9.29 mmol), N-hydroxyphthalimide (1.70 g, 10.22 mmol) and triphenylphosphine (2.70 g, 10.22 mmol) in dry tetrahydrofuran (40 mL), under an argon atmosphere, was stirred at 0 ^{0}C for 20 minutes. A solution of diisopropyl azodicarboxylate (2.10 g, 10.22 mmol) in tetrahydrofuran (20 mL) was then added dropwise and allowed to slowly warm to room temperature (2 hours). Upon completion, the reaction was concentrated *in vacuo*. The resulting residue was purified *via* medium

pressure liquid chromatography (SiO_2, 100% hexanes to 60% ethyl acetate / hexanes to provide the title compound (3.0 g, 89% yield). ^1H NMR (500 MHz, Chloroform-*d*) δ 7.83 (dd, J = 5.4, 3.1 Hz, 2H), 7.74 (dd, J = 5.5, 3.1 Hz, 2H), 4.06 (m, 2H), 3.87 (m, 1H), 2.93 – 2.78 (m, 2H), 2.08 – 1.93 (m, 1H), 1.70 (m, 1H), 1.53-1.34 (m, 4H), 1.46 (s, 9H).

(3*S*)-3-[(Aminooxy)methyl]piperidine dihydrochloride (7-*S*). To a solution of *tert*-butyl 3-*S*-{[(1,3-dioxo-1,3-dihydro-2*H*-isoindol-2-yl)oxy]methyl}piperidine-1-carboxylate **13f** (3.0 g, 8.34 mmol) in dry dichloromethane (42 mL), under an argon atmosphere was added hydrazine monohydrate (0.70 mL, 8.75 mmol) dropwise during 5 minutes. The solution turned into a suspension in 30 minutes. After 2 hours, the white precipitate was filtered off and washed with cold dichloromethane (20 mL). The filtrate was concentrated *in vacuo* and the resulting residue was used without further purification. The residue was taken up in methanol (10 mL) and treated dropwise with hydrochloric acid (4.0 N in dioxane, 10.50 mL, 41.70 mmol). The mixture was stirred at rt, under an argon atmosphere, for 2 hours and concentrated. The resulting residue was taken up in water (20 mL), washed with ethyl acetate (3 x 10 mL) and lyophilized to give the di-HCl salt of 3-*S*-[(aminooxy)methyl]piperidine (**7-*S***) as a semi-solid hygroscopic material (1.25 g, 74% yield for two steps). ^1H NMR (500 MHz, D_2O) δ 3.91 (dd, J = 8.5, 5.0 Hz, 1H), 3.83 (dd, J = 8.6, 7.1 Hz, 1H), 3.35 – 3.28 (m, 1H), 3.27 – 3.20 (m, 1H), 2.81 – 2.65 (m, 2H), 2.14 – 2.01 (m, 1H), 1.81 (dq, J = 13.8, 3.3 Hz, 1H), 1.74 (ddt, J = 15.3, 4.0, 2.0 Hz, 1H), 1.57 (dtt, J = 14.5, 12.9, 4.0 Hz, 1H), 1.23 (qd, J = 12.7, 3.9 Hz, 1H). ^{13}C NMR (126 MHz, D_2O) δ 76.02, 45.40, 43.97, 32.36, 23.92, 21.16. HRMS (ESI) m/z calculated for $C_6H_{15}N_2O$ [M+H], 131.1184; found 131.1208. $[\alpha]_D^{20}$ = +4.2 (*c* 0.01, H_2O).

2-[(Aminooxy)methyl] morpholine dihydrochloride (8).

***tert*-Butyl 2-{[(1,3-dioxo-1,3-dihydro-2*H*-isoindol-2-yl)oxy]methyl}morpholine-4-carboxylate (13g).** A mixture of *tert*-butyl 2-(hydroxymethyl) morpholine-4-carboxylate **12g** (2.0 g, 9.21 mmol), N-hydroxyphthalimide (1.66 g, 10.1 mmol) and triphenylphosphine (2.70 g, 10.1 mmol) in dry tetrahydrofuran (26 mL), under an argon atmosphere, was stirred at 0 ^0C for 20 minutes. A solution of diisopropyl azodicarboxylate (2.05 g, 10.13 mmol) in tetrahydrofuran (20 mL) was then added dropwise and allowed to slowly warm to room temperature (2 hours). Upon completion, the reaction was concentrated *in vacuo*. The resulting residue was purified *via* medium pressure liquid chromatography (SiO_2, 100% hexanes to 50% ethyl acetate / hexanes (2.70 g, 81% yield). ^1H NMR (500 MHz, Chloroform-d) δ 7.86 (dd, J = 5.4, 3.1 Hz, 2H), 7.77 (dd, J = 5.5, 3.1 Hz, 2H), 4.27 (dd, J = 10.9, 6.3 Hz, 1H), 4.20 (dd, J = 10.9, 3.7 Hz, 1H), 4.03 (d, J = 13.2 Hz, 1H), 3.91 – 3.85 (m, 2H), 3.55 (m, 2H), 2.92 (m, 2H), 1.48 (s, 9H). Melting point: 168 ^oC (Decomposition).

2-[(Aminooxy)methyl] morpholine dihydrochloride (8). To a solution of *tert*-butyl 2-{[(1,3-dioxo-1,3-dihydro-2*H*-isoindol-2-yl)oxy]methyl}morpholine-4-carboxylate **12g** (0.45 g, 1.25 mmol) in dry dichloromethane (3 mL), under an argon atmosphere was added hydrazine monohydrate (0.10 mL) dropwise during 5 minutes. The solution turned into a suspension in 30 minutes. After 2 hours, the white precipitate was filtered off and washed with cold dichloromethane (2 mL). The filtrate was concentrated in vacuo and the resulting residue was used without further purification. The residue was taken up in methanol (1 mL) and treated dropwise with hydrochloric acid (4.0 N in dioxane, 1.60 mL, 6.25 mmol). The mixture was stirred at rt, under an argon atmosphere, for 2 hours and concentrated. The resulting residue was taken up in

water (2 mL), washed with ethyl acetate (3 x 5 mL) and lyophilized to give the di-HCl salt of 3-[(aminooxy)methyl] morpholine (**8**) as a semi-solid hygroscopic material (0.20 g, 78% yield for two steps). ^{1}H NMR (500 MHz, D_2O) δ 4.12 – 4.05 (m, 1H), 3.99 (m, 3H), 3.70 (m, 1H), 3.26 – 3.13 (m, 2H), 3.04 (m, 1H), 2.96 (m, 1H). ^{13}C NMR (126 MHz, D_2O) δ 73.23, 45.00, 43.31, 33.60, 24.29. HRMS (ESI) m/z calculated for $C_5H_{13}N_2O_2$ [M+H], 133.0977; found 133.1000.

4-(Aminooxy)piperidine dihydrochloride (9).

***tert*-Butyl 4-[(1,3-dioxo-1,3-dihydro-2*H*-isoindol-2-yl)oxy]piperidine-1-carboxylate (13h).** A mixture of *tert*-butyl 4-hydroxypiperidine-1-carboxylate **12h** (1.14 g, 5.68 mmol), N-hydroxyphthalimide (1.02 g, 6.24 mmol) and triphenylphosphine (1.70 g, 6.24 mmol) in dry tetrahydrofuran (30 mL), under an argon atmosphere, was stirred at 0 ºC for 20 minutes. A solution of diisopropyl azodicarboxylate (1.30 g, 6.24 mmol) in tetrahydrofuran (10 mL) was then added dropwise and allowed to slowly warm to room temperature (2 hours). Upon completion, the reaction was concentrated *in vacuo*. The resulting residue was purified *via* medium pressure liquid chromatography (SiO_2, 100% hexanes to 40% ethyl acetate / hexanes to provide the title compound (1.54 g, 75% yield). ^{1}H NMR (500 MHz, Chloroform-*d*) δ 7.78 (dd, J = 5.4, 3.1 Hz, 2H), 7.70 (dd, J = 5.5, 3.1 Hz, 2H), 4.99 (p, J = 6.3 Hz, 1H), 3.85 (m, 4H), 3.03 (m, 4H), 1.47 (s, 9H).

4-(Aminooxy)piperidine dihydrochloride (9). To a solution of *tert*-butyl 4-[(1,3-dioxo-1,3-dihydro-2*H*-isoindol-2-yl)oxy]piperidine-1-carboxylate **13h** (0.76 g, 2.20 mmol) in dry dichloromethane (22 mL), under an argon atmosphere was added hydrazine monohydrate (0.15 mL, 2.42 mmol) dropwise during 5 minutes. The solution turned into a suspension in 30 minutes. After 2 hours, the white precipitate was filtered off and washed with cold dichloromethane (5 mL). The filtrate was concentrated in vacuo and the resulting residue was used without further purification. The residue was taken up in methanol (5 mL) and treated dropwise with hydrochloric acid (4.0 N in dioxane, 3.0 mL, 12.0 mmol). The mixture was stirred at rt, under an argon atmosphere, for 2 hours and concentrated. The resulting residue was taken up in water (5 mL), washed with ethyl acetate (3 x 5 mL) and lyophilized to give the di-HCl salt of 4-(aminooxy)piperidine (**9**) as a semi-solid hygroscopic material (0.32 g, 77% yield for two steps). ^{1}H NMR (500 MHz, D_2O) δ 4.31 (tt, J = 6.8, 3.5 Hz, 1H), 3.30 (ddd, J = 12.9, 9.0, 3.6 Hz, 2H), 3.06 (ddd, J = 13.1, 6.9, 4.0 Hz, 2H), 2.20 – 2.10 (m, 2H), 1.97 – 1.87 (m, 2H). ^{13}C NMR (126 MHz, D_2O) δ 73.49, 71.25, 63.25, 42.60, 42.22. HRMS (ESI) m/z calculated for $C_5H_{13}N_2O$ [M+H], 117.1028; found 117.1025.

3-[(Aminooxy)methyl]pyrrolidine dihydrochloride (10).

***tert*-Butyl-3-{[(1,3-dioxo-1,3-dihydro-2*H*-isoindol-2-yl)oxy]methyl}pyrrolidine-1-carboxylate (13i).** A mixture of *tert*-butyl-3-(hydroxymethyl)pyrrolidine-1-carboxylate **12i** (2.00 g, 9.90 mmol), N-hydroxyphthalimide (1.80 g, 10.94 mmol) and triphenylphosphine (2.87 g, 10.9 mmol) in dry tetrahydrofuran (50 mL), under an argon atmosphere, was stirred at 0 ^{0}C for 20 minutes. A solution of diisopropyl azodicarboxylate (2.21 g, 10.9 mmol) in tetrahydrofuran (20 mL) was then added dropwise and allowed to slowly warm to room temperature (2 hours). Upon completion, the reaction was concentrated *in vacuo*. The resulting residue was purified *via* medium pressure liquid chromatography (SiO_2, 100% hexanes to 60% ethyl acetate / hexanes to provide the title compound (2.93 g, 85% yield) . ^{1}H NMR (500 MHz, Chloroform-*d*) δ 7.84 (ddd, J = 5.5, 3.1, 1.0 Hz, 2H), 7.79 – 7.71 (m, 2H), 4.16 (dd, J = 7.3, 3.9 Hz, 2H), 3.62 (dd, J = 11.1, 7.6 Hz,

1H), 3.49 (ddd, J = 10.6, 8.1, 4.8 Hz, 1H), 3.36 (dt, J = 10.9, 7.6 Hz, 1H), 3.24 (dd, J = 11.1, 6.9 Hz, 1H), 2.70 (hept, J = 7.2 Hz, 1H), 2.19 – 2.06 (m, 2H), 1.46 (s, 9H).

3-[(Aminooxy)methyl]pyrrolidine dihydrochloride (10). To a solution of *tert*-butyl {3-[(1,3-dioxo-1,3-dihydro-2*H*-isoindol-2-yl)oxy]propyl}carbamate **13i** (0.500 g, 1.45 mmol) in dry dichloromethane (5 mL), under an argon atmosphere was added hydrazine monohydrate (0.14 mL, 1.45 mmol) dropwise during 5 minutes. The solution turned into a suspension in 30 minutes. After 2 hours, the white precipitate was filtered off and washed with cold dichloromethane (5 mL). The filtrate was concentrated in vacuo and the resulting residue was used without further purification. The residue was taken up in methanol (2 mL) and treated dropwise with hydrochloric acid (4.0 N in dioxane, 2.0 mL, 8.0 mmol). The mixture was stirred at rt, under an argon atmosphere, for 2 hours and concentrated. The resulting residue was taken up in water (5 mL), washed with ethyl acetate (3 x 5 mL) and lyophilized to give 3-[(aminooxy)methyl]pyrrolidine dihydrochloride (**10**) as a fine tan powder (0.23 g, 75% yield for two steps). ^{1}H NMR (500 MHz, D_2O) δ 3.98 (dd, J = 8.5, 5.7 Hz, 1H), 3.91 (dd, J = 8.5, 6.8 Hz, 1H), 3.35 (dd, J = 12.2, 8.3 Hz, 1H), 3.24 (ddd, J = 11.9, 8.3, 5.4 Hz, 1H), 3.15 (dt, J = 11.7, 7.9 Hz, 1H), 2.98 (dd, J = 12.2, 7.7 Hz, 1H), 2.70 – 2.57 (m, 1H), 2.07 (dtd, J = 13.3, 7.9, 5.4 Hz, 1H), 1.69 (dq, J = 13.5, 8.1 Hz, 1H). ^{13}C NMR (126 MHz, D_2O) δ 75.11, 46.88, 45.19, 35.48, 26.17. HRMS (ESI) m/z calculated for $C_5H_{13}N_2O$ [M+H], 117.1028; found 117.1049. Melting point: 340 ^{0}C (Decomposition).

(3*R*)-3-[(Aminooxy)methyl]pyrrolidine dihydrochloride (10-*R*).

***tert*-Butyl (3*R*)-3-{[(1,3-dioxo-1,3-dihydro-2*H*-isoindol-2 yl)oxy]methyl}pyrrolidine-1-carboxylate (13j).** A mixture of *tert*-butyl (3*R*)-3-(hydroxymethyl)pyrrolidine-1-carboxylate **12j** (2.00 g, 9.94 mmol), N-hydroxyphthalimide (1.80 g, 10.94 mmol) and triphenylphosphine (2.87 g, 10.9 mmol) in dry tetrahydrofuran (50 mL), under an argon atmosphere, was stirred at 0 ^{0}C for 20 minutes. A solution of diisopropyl azodicarboxylate (2.21 g, 10.94 mmol) in tetrahydrofuran (20 mL) was then added dropwise and allowed to slowly warm to room temperature (2 hours). Upon completion, the reaction was concentrated *in vacuo*. The resulting residue was purified *via* medium pressure liquid chromatography (SiO_2, 100% hexanes to 60% ethyl acetate / hexanes (2.93 g, 85% yield) to provide the title compound. ^{1}H NMR (500 MHz, Chloroform-*d*) δ 7.88 – 7.81 (m, 2H), 7.80 – 7.73 (m, 2H), 4.16 (dt, J = 8.7, 4.5 Hz, 2H), 3.66 – 3.59 (m, 1H), 3.49 (ddd, J = 10.7, 8.1, 4.8 Hz, 1H), 3.37 (dt, J = 10.8, 7.7 Hz, 1H), 3.24 (dd, J = 11.1, 6.8 Hz, 1H), 2.70 (m, 1H), 2.20 – 2.07 (m, 2H), 1.47 (s, 9H).

(3*R*)-3-[(Aminooxy)methyl]pyrrolidine dihydrochloride (10-*R*). To a solution of *tert*-butyl (3*R*)-3-{[(1,3-dioxo-1,3-dihydro-2*H*-isoindol-2 yl)oxy]methyl}pyrrolidine-1-carboxylate **13j** (0.50 g, 1.45 mmol) in dry dichloromethane (5 mL), under an argon atmosphere was added hydrazine monohydrate (0.14 mL, 1.45 mmol) dropwise during 5 minutes. The solution turned into a suspension in 30 minutes. After 2 hours, the white precipitate was filtered off and washed with cold dichloromethane (5 mL). The filtrate was concentrated *in vacuo* and the resulting residue was used without further purification. The residue was taken up in methanol (2 mL) and treated dropwise with hydrochloric acid (4.0 N in dioxane, 2.0 mL, 8.0 mmol). The mixture was stirred at rt, under an argon atmosphere, for 2 hours and concentrated. The resulting residue was taken up in water (5 mL), washed with ethyl acetate (3 x 5 mL) and lyophilized to give the di-HCl salt of (3*R*)-3-[(aminooxy)methyl]pyrrolidine (**10-*R***) as a fine tan powder (0.23 g, 75% yield for two steps). ^{1}H NMR (500 MHz, D_2O) δ 4.02 (dd, J = 8.3, 5.7 Hz, 1H), 3.95 (dd, J = 8.4, 6.7 Hz, 1H), 3.40 –

3.32 (m, 1H), 3.26 – 3.20 (m, 1H), 3.20 – 3.11 (m, 1H), 2.98 (dd, J = 12.2, 7.7 Hz, 1H), 2.64 (dt, J = 14.7, 7.4 Hz, 1H), 2.13 – 2.03 (m, 1H), 1.70 (dd, J = 13.6, 8.2 Hz, 1H). ^{13}C NMR (126 MHz, D_2O) δ 75.24, 47.01, 45.31, 35.60, 26.29. HRMS (ESI) m/z calculated for $C_5H_{13}N_2O$ [M+H], 117.1028; found 117.1034. Melting point: 348 ^{0}C (Decomposition). $[\alpha]_D^{20}$ = +6.4 (c 0.01, H_2O).

({5-Hydroxy-6-methyl-4-[(E)-({[(3*R*)-pyrrolidin-3-yl]methoxy}imino)methyl]pyridin-3-yl}methoxy)phosphonic acid (10-*R*-PLP)

To a solution of (3*R*)-3-[(aminooxy)methyl]pyrrolidine dihydrochloride ***10-R*** (0.12 g, 0.48 mmol) in PBS buffer (2.0 mL, pH 7.40) was added pyridoxal 5′-phosphate **2** (0.09 g, 0.48 mmol). The resulting mixture was stirred at rt for 18 h. Subsequently, the reaction mixture was passed through a sintered glass filter. The filtrate was lyophilized to give the title compound a semi-solid hygroscopic material (0.13 g, 80% yield). ^{1}H NMR (500 MHz, D_2O) δ 8.60 (s, 1H), 8.07 (s, 1H), 4.97 (d, J = 7.8 Hz, 2H), 4.36 (dd, J = 11.1, 5.8 Hz, 1H), 4.28 (dd, J = 11.0, 7.0 Hz, 1H), 3.39 (dd, J = 12.1, 8.2 Hz, 1H), 3.30 (ddd, J = 13.1, 8.3, 5.2 Hz, 1H), 3.18 (dt, J = 11.9, 8.1 Hz, 1H), 3.05 (dd, J = 12.1, 8.0 Hz, 1H), 2.76 (dt, J = 13.5, 7.5 Hz, 1H), 2.53 (s, 3H), 2.11 (dtd, J = 13.1, 7.8, 5.2 Hz, 1H), 1.75 (dq, J = 13.4, 8.3 Hz, 1H). ^{13}C NMR (126 MHz, D_2O) δ 152.76, 146.69, 144.65, 133.46, 133.40, 129.72, 127.29, 76.24, 61.61, 47.34, 45.29, 36.83, 26.50, 14.24. ^{31}P NMR (202 MHz, D_2O) δ -2.39. HRMS (ESI) m/z calculated for $C_{13}H_{21}N_3O_6P$ [M+H], 346.1168; found 346.1170. HRMS (ESI) m/z calculated for $C_{13}H_{19}N_3O_6P$ [M-H], 344.1011; found 344.1014. Purity: 99.6% by HPLC. $[\alpha]_D^{20}$ = +4.2 (c 0.01, H_2O).

(3*S*)-*S*-[(Aminooxy)methyl]pyrrolidine dihydrochloride (10-*S*).

***tert*-Butyl (3*S*)-3-{[(1,3-dioxo-1,3-dihydro-2*H*-isoindol-2 yl)oxy]methyl}pyrrolidine-1-carboxylate (13k).** A mixture of *tert*-butyl (3*S*)-3-(hydroxymethyl)pyrrolidine-1-carboxylate **12k** (2.00 g, 9.94 mmol), N-hydroxyphthalimide (1.80 g, 10.9 mmol) and triphenylphosphine (2.87 g, 10.9 mmol) in dry tetrahydrofuran (50 mL), under an argon atmosphere, was stirred at 0 °C for 20 minutes. A solution of diisopropyl azodicarboxylate (2.21 g, 10.94 mmol) in tetrahydrofuran (20 mL) was then added dropwise and allowed to slowly warm to room temperature (2 hours). Upon completion, the reaction was concentrated *in vacuo*. The resulting residue was purified *via* medium pressure liquid chromatography (SiO_2, 100% hexanes to 60% ethyl acetate / hexanes (2.93 g, 85% yield) to provide the title compound. ^{1}H NMR (500 MHz, Chloroform-*d*) δ 7.84 (ddd, J = 5.5, 3.1, 1.0 Hz, 2H), 7.79 – 7.71 (m, 2H), 4.16 (dd, J = 7.3, 3.9 Hz, 2H), 3.62 (dd, J = 11.1, 7.6 Hz, 1H), 3.49 (ddd, J = 10.6, 8.1, 4.8 Hz, 1H), 3.36 (dt, J = 10.9, 7.6 Hz, 1H), 3.24 (dd, J = 11.1, 6.9 Hz, 1H), 2.70 (hept, J = 7.2 Hz, 1H), 2.19 – 2.06 (m, 2H), 1.46 (s, 9H).

(3*S*)-3-[(Aminooxy)methyl]pyrrolidine dihydrochloride (10-*S*). To a solution of *tert*-butyl (3*S*)-3-{[(1,3-dioxo-1,3-dihydro-2*H*-isoindol-2-yl)oxy]methyl}pyrrolidine-1-carboxylate **13k** (2.20 g, 6.36 mmol) in dry dichloromethane (32 mL), under an argon atmosphere was added hydrazine monohydrate (0.6 mL, 6.68 mmol) dropwise during 5 minutes. The solution turned into a suspension in 30 minutes. After 2 hours, the white precipitate was filtered off and washed with cold dichloromethane (10 mL). The filtrate was concentrated *in vacuo* and the resulting residue was used without further purification. The residue was taken up in methanol (10 mL) and treated dropwise with hydrochloric acid (4.0 N in dioxane, 8.0 mL, 32.0 mmol). The mixture was stirred at rt, under an argon atmosphere, for 2 hours and concentrated. The resulting residue was taken up in water (20 mL), washed with ethyl acetate (3 x 10 mL) and lyophilized to give the di-HCl salt of (3*S*)-3-[(aminooxy)methyl]pyrrolidine (**6-*S***) as a fine tan powder (0.960 g, 80% yield for two

steps). ^{1}H NMR (500 MHz, D_2O) δ 4.05 – 3.98 (m, 1H), 3.98 – 3.91 (m, 1H), 3.36 (dd, J = 12.2, 8.4 Hz, 1H), 3.25 (ddd, J = 11.8, 8.2, 5.4 Hz, 1H), 3.16 (dt, J = 11.8, 8.0 Hz, 1H), 2.99 (dd, J = 12.2, 7.7 Hz, 1H), 2.64 (tt, J = 14.0, 7.8 Hz, 1H), 2.08 (dtd, J = 13.3, 7.8, 5.4 Hz, 1H), 1.70 (dq, J = 13.4, 8.1 Hz, 1H). ^{13}C NMR (126 MHz, D_2O) δ 75.23, 47.00, 45.31, 35.60, 26.29. HRMS (ESI) m/z calculated for $C_5H_{13}N_2O$ [M+H], 117.1028; found 117.1033. Melting point: 345 ^{0}C (Decomposition). $[\alpha]_D^{20}$ = -6.9 (*c* 0.01, H_2O).

({5-Hydroxy-6-methyl-4-[(E)-({[(3*S*)-pyrrolidin-3-yl]methoxy}imino)methyl]pyridin-3-yl}methoxy)phosphonic acid (10-*S*-PLP)

To a solution of (3*S*)-3-[(aminooxy)methyl]pyrrolidine dihydrochloride **10-*S*** (0.12 g, 0.48 mmol) in PBS buffer (2.0 mL, pH 7.40) was added pyridoxal 5′-phosphate (0.09 g, 0.48 mmol). The resulting mixture was stirred at rt for 18 h. Subsequently, the reaction mixture was passed through a sintered glass filter. The filtrate was lyophilized to give a white fine powder, which was purified by reverse phase chromatography (C18, 100% ammonium formate to 80% methanol / ammonium formate) to give the title compound as a tan solid (0.14 g, 70% yield). ^{1}H NMR (500 MHz, D_2O) δ 8.47 (s, 1H), 8.28 (s, 1H), 7.67 (s, 1H), 4.88 (d, J = 6.7 Hz, 2H), 4.24 (dd, J = 11.5, 5.4 Hz, 1H), 4.14 (dd, J = 11.0, 7.3 Hz, 1H), 3.38 (t, J = 10.2 Hz, 1H), 3.30 – 3.24 (m, 1H), 3.17 (q, J = 9.1 Hz, 1H), 3.05 (dd, J = 11.9, 8.1 Hz, 1H), 2.73 (p, J = 7.4 Hz, 1H), 2.32 (s, 3H), 2.08 (dt, J = 13.1, 6.4 Hz, 1H), 1.79 – 1.69 (m, 1H). ^{13}C NMR (126 MHz, D_2O) δ 147.40, 124.22, 122.61, 110.37, 109.89, 75.16, 62.67, 47.61, 45.21, 37.16, 26.53, 15.78. ^{31}P NMR (202 MHz, D_2O) δ -0.26. HRMS (ESI) m/z calculated for $C_{13}H_{21}N_3O_6P$ [M+H], 346.1168; found 346.1169. Melting point: 225 ^{0}C (Decomposition). Purity: 98.6% by HPLC. $[\alpha]_D^{20}$ = -3.5 (*c* 0.01, H_2O).

3-[(Aminooxy)methyl]-3-fluoropyrrolidine di-formic acid. (11)

***tert*-Butyl 3-formylpyrrolidine-1-carboxylate (14).** To a solution of *tert*-butyl 3-(hydroxymethyl)pyrrolidine-1-carboxylate **12i** (2.0 g, 9.94 mmol) in dichloromethane (50.0 mL, 0.2M) was slowly added Dess-Martin periodinane (DMP) (6.40 g, 14.91 mmol) and the mixture was stirred for 1 h at rt after which water (0.20 mL) was added dropwise and the resulting mixture was stirred at rt for 1 h. Subsequently, the reaction mixture was diluted with diethyl ether (500 mL) and sodium bicarbonate: sodium thiosulfate (1:1, 500 mL) was added. A suspension was observed which was filtered through a sintered glass funnel and the filtrate was transferred to a separatory funnel and the two layers were separated. The aqueous layer was back extracted with diethyl ether. The combined organic layers were dried over anhydrous sodium sulfate and concentrated in vacuo to give *tert*-butyl 3-formylpyrrolidine-1-carboxylate (**14**) in quantitative yield which was used without further purification. ^{1}H NMR (500 MHz, Chloroform-*d*) δ 9.69 (d, J = 1.6 Hz, 1H), 3.74 - 3.69 (m, 1H), 3.68 - 3.52 (m, 2H), 3.47 - 3.26 (m, 1H), 3.01 (m, 1H), 2.33 - 1.95 (m, 2H), 1.48 (s, 9H).

***tert*-Butyl 3-fluoro-3-formylpyrrolidine-1-carboxylate (15).** To a solution of *tert*-butyl 3-formylpyrrolidine-1-carboxylate **14** (1.98 g, 9.94 mmol) in methyl *tert*-butyl ether (MTBE) (20 mL) was added pyrrolidine (10 mol %, 0.15 mL) and the mixture stirred for 5 min at room temperature, after which N-fluorobenzenesulfonimide (NFSI) (3.80 g, 11.93 mmol) was added and the resulting mixture stirred at 40 °C for 18 h. The reaction mixture was diluted with tert-butyl methyl ether (100 mL) and passed through a sintered glass filter. The volatiles were concentrated *in vacuo* and the resulting residue was purified *via* medium pressure liquid chromatography (SiO_2,

100% hexanes to 60% ethyl acetate / hexanes) to provide the title compound (1.80 g, 82% yield). ^{1}H NMR (500 MHz, Chloroform-*d*) δ 9.88 (s, 1H), 3.86 – 3.46 (m, 4H), 2.20 – 2.11 (m, 2H), 1.46 (s, 9H).

***tert*-Butyl 3-fluoro-3-(hydroxymethyl)pyrrolidine-1-carboxylate (16).** To a solution of *tert*-butyl 3-fluoro-3-formylpyrrolidine-1-carboxylate **15** (1.80 g, 8.30 mmol) in methanol (80.0 mL) was added sodium borohydride (0.95 g, 24.90 mmol) and the reaction mixture stirred for 30 min at room temperature. Water was then added and the aqueous phase extracted twice with ethyl acetate after which the combined organic phases were washed with brine, dried over anhydrous sodium sulfate, filtered and concentrated in *vacuo*. The resulting residue was purified *via* medium pressure liquid chromatography (SiO_2, 100% hexanes to 60% ethyl acetate / hexanes) to provide the title compound (1.70 g, 90% yield). ^{1}H NMR (500 MHz, Chloroform-*d*) δ 3.80 (m, 2H), 3.62 (m, 2H), 3.55 – 3.41 (m, 2H), 2.17 (m, 1H), 1.99 (m, 1H), 1.46 (s, 9H).

***tert*-Butyl 3-{[(1,3-dioxo-1,3-dihydro-2*H*-isoindol-2-yl)oxy]methyl}-3-fluoropyrrolidine-1-carboxylate (17).** A mixture of_*tert*-butyl 3-fluoro-3-(hydroxymethyl)pyrrolidine-1-carboxylate (1.20 g, 5.48 mmol), N-hydroxyphthalimide (0.99 g, 6.03 mmol) and triphenylphosphine (1.60 g, 6.03 mmol) in dry tetrahydrofuran (20 mL), under an argon atmosphere, was stirred at 0 ^{0}C for 20 minutes. A solution of diisopropyl azodicarboxylate (1.22 g, 6.03 mmol) in tetrahydrofuran (10 mL) was then added dropwise and allowed to slowly warm to room temperature (2 hours). Upon completion, the mixture was concentrated *in vacuo*. The resulting residue was then purified *via* medium pressure liquid chromatography (SiO_2, 100% hexanes to 60% ethyl acetate / hexanes to provide the title compound (1.62 g, 81% yield). ^{1}H NMR (500 MHz, Chloroform-*d*) δ 7.80 (m, 2H), 7.74 (m, 2H), 4.55 – 4.21 (m, 2H), 3.76 (m, 1H), 3.63 (m, 2H), 3.50 (m, 1H), 2.43 – 2.06 (m, 2H), 1.43 (s, 9H).

3-[(Aminooxy)methyl]-3-fluoropyrrolidine di-formic acid (11). To a solution *tert*-butyl 3-{[(1,3-dioxo-1,3-dihydro-2*H*-isoindol-2-yl)oxy]methyl}-3-fluoropyrrolidine-1-carboxylate **17** (1.00 g, 2.75 mmol) in dry dichloromethane (15 mL), under an argon atmosphere was added hydrazine monohydrate (0.28 mL, 2.90 mmol) dropwise during 5 minutes. The solution turned into a suspension in 30 minutes. After 2 hours, the white precipitate was filtered off and washed with cold dichloromethane (5 mL). The filtrate was concentrated *in vacuo* and the resulting residue used without further purification. The residue was taken up in methanol (10 mL) and treated dropwise with hydrochloric acid (4.0 N in dioxane, 3.50 mL, 14.0 mmol). The mixture was stirred at rt, under an argon atmosphere, for 2 hours and concentrated. The resulting residue was treated with water (10 mL), washed with ethyl acetate (3 x 10 mL) and lyophilized to give a fine white powder. The resulting salt was purified by reverse phase chromatography (C18, 100% ammonium formate to 80% methanol / ammonium formate) to give the title compound a semi-solid hygroscopic material (0.45 g, 72% yield for two steps). ^{1}H NMR (500 MHz, D_2O) δ 4.45 – 4.26 (m, 2H), 3.61 (m, 1H), 3.47 – 3.35 (m, 3H), 2.34 (m, 1H), 2.14 (m, 1H). ^{13}C NMR (126 MHz, D_2O) δ 102.02 (d, *J* = 179.3 Hz), 75.81 (d, *J* = 22.0 Hz), 52.13 (d, *J* = 26.2 Hz), 44.07, 32.39 (d, *J* = 23.0 Hz). HRMS (ESI) m/z calculated for $C_5H_{12}FN_2O$ [M+H], 135.0934; found 135.0947.

({4-[(E)-{[(3-Fluoropyrrolidin-3-yl)methoxy]imino}methyl]-5-hydroxy-6-methylpyridin-3-yl}methoxy)phosphonic acid (11-PLP)

To a solution of 3-[(aminooxy)methyl]-3-fluoropyrrolidine di-formic acid **11** (0.093 g, 0.42 mmol) in PBS buffer (2.0 mL, pH 7.40) was added pyridoxal 5′-phosphate (0.11 g, 0.42 mmol). The resulting mixture was stirred at rt for 18 h. Subsequently, the reaction mixture was passed through a sintered glass filter. The filtrate was lyophilized to give a semi-solid hygroscopic material (0.12 g, 73% yield). ^{1}H NMR (500 MHz, D_2O) δ 8.63 (s, 1H), 8.07 (s, 1H), 4.97 (d, J = 7.8 Hz, 2H), 3.60 (m, 1H), 3.50 – 3.37 (m, 3H), 2.51 (s, 3H), 2.40 – 2.09 (m, 2H). ^{13}C NMR (126 MHz, D_2O) δ 152.77, 147.20, 144.75, 133.80, 133.74, 129.80, 127.39, 102.94, 101.50, 75.91, 75.73, 61.72, 61.68, 52.18, 51.97, 44.19, 32.55, 32.37, 14.34. ^{13}C NMR (126 MHz, D_2O) δ 152.77, 147.20, 144.75, 133.77 (d, J = 7.3 Hz), 129.80, 127.39, 102.94, 101.50, 75.82 (d, J = 23.0 Hz), 61.70 (d, J = 4.3 Hz), 52.07 (d, J = 26.1 Hz), 44.19, 32.46 (d, J = 22.8 Hz), 14.34. HRMS (ESI) m/z calculated for $C_{13}H_{20}FN_3O_6P$ [M+H], 364.1074; found 364.1078. Purity: 99.1% by HPLC.

5) <u>Computational Modeling Validation</u>

The parameters and topologies for the selected minimized structures (protein+ligands) were created using the Leap module of Amber Tools 2020 (https://ambermd.org/doc12/Amber20.pdf). For this step, the amber force field (GAFF) and AMBERff14sb forcefields were utilized [5]. The AM1-BCC charge scheme was considered for the different ligands and PLP to calculate the different partial charges, and fitted to GAFF utilizing antechamber [6]. For the molecular dynamics simulations, each system was neutralized in accordance with Joung and Cheatham parameters, i.e, 0.1 M NaCl, replicating the ionic biological environment[4]. Regarding solvation models, the TIP4P-Ew water model was utilized [7, 8]. The minimization step was performed with a series of harmonic potentials, with different restrictions, and heated from 0 to 300 K in several steps. After the heating step, 30 ns simulations were performed in triplicate, at 300K and 1 atm pressure, with a 2 fs timestep. The SHAKE algorithm was also implemented to constrain the hydrogen bonds [9]. The convergence of the simulations was assured by converged RMSDs for all systems.

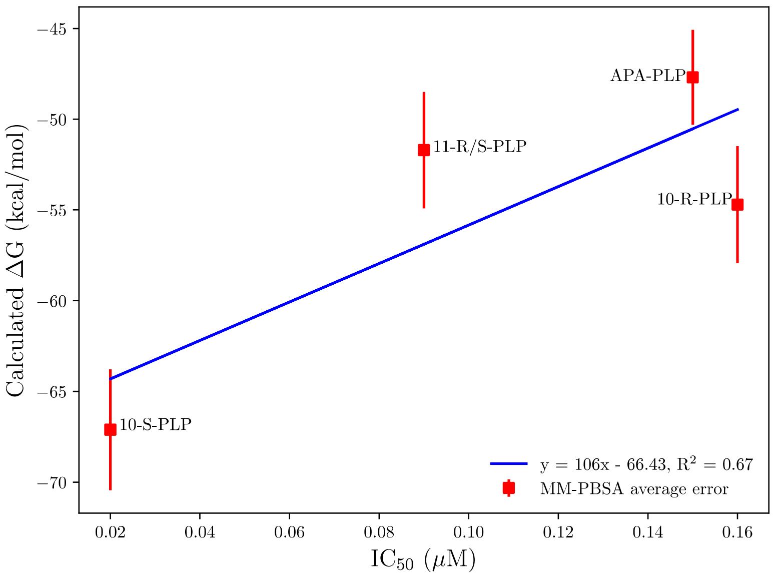

Figure S1 - Averaged calculated binding energies of selected analogs covalently bound to PLP calculated with MM-PBSA (y-axis), correlated to IC_{50} values (x-axis). Error bars are depicted in red (MM-PBSA).

For MM-PBSA free energy calculations, the initial 50 frames of the simulation were considered, and their binding energy averaged among the triplicate simulations.

To probe and validate the computational affinities, relative to those measured experimentally (**Table 1**), selected compounds (**Figure S1**) were compared to measured ODC IC_{50} values with moderately good correlation.

6) References to Supplementary Materials

(1) Battye, T. G.; Kontogiannis, L.; Johnson, O.; Powell, H. R.; Leslie, A. G. iMOSFLM: a new graphical interface for diffraction-image processing with MOSFLM. *Acta Crystallogr D Biol Crystallogr* **2011**, *67* (Pt 4), 271-281. DOI: 10.1107/S0907444910048675.
(2) Dufe, V. T.; Ingner, D.; Heby, O.; Khomutov, A. R.; Persson, L.; Al-Karadaghi, S. A structural insight into the inhibition of human and Leishmania donovani ornithine decarboxylases by 1-amino-oxy-3-aminopropane. *Biochem J* **2007**, *405* (2), 261-268. DOI: BJ20070188 [pii]10.1042/BJ20070188.
(3) Emsley, P.; Lohkamp, B.; Scott, W. G.; Cowtan, K. Features and development of Coot. *Acta Crystallogr D Biol Crystallogr* **2010**, *66* (Pt 4), 486-501. DOI: 10.1107/S0907444910007493.
(4) Adams, P. D.; Afonine, P. V.; Bunkoczi, G.; Chen, V. B.; Davis, I. W.; Echols, N.; Headd, J. J.; Hung, L. W.; Kapral, G. J.; Grosse-Kunstleve, R. W.; et al. PHENIX: a comprehensive Python-based system for macromolecular structure solution. *Acta Crystallogr D Biol Crystallogr* **2010**, *66* (Pt 2), 213-221. DOI: 10.1107/S0907444909052925.
(5) Maier, J. A.; Martinez, C.; Kasavajhala, K.; Wickstrom, L.; Hauser, K. E.; Simmerling, C. ff14SB: Improving the Accuracy of Protein Side Chain and Backbone Parameters from ff99SB. *J Chem Theory Comput* **2015**, *11* (8), 3696-3713. DOI: 10.1021/acs.jctc.5b00255.
(6) Jakalian, A.; Jack, D. B.; Bayly, C. I. Fast, efficient generation of high-quality atomic charges. AM1-BCC model: II. Parameterization and validation. *J Comput Chem* **2002**, *23* (16), 1623-1641. DOI: 10.1002/jcc.10128.
(7) Dopke, M. F.; Moultos, O. A.; Hartkamp, R. On the transferability of ion parameters to the TIP4P/2005 water model using molecular dynamics simulations. *J Chem Phys* **2020**, *152* (2), 024501. DOI: 10.1063/1.5124448.
(8) Horn, H. W.; Swope, W. C.; Pitera, J. W.; Madura, J. D.; Dick, T. J.; Hura, G. L.; Head-Gordon, T. Development of an improved four-site water model for biomolecular simulations: TIP4P-Ew. *J Chem Phys* **2004**, *120* (20), 9665-9678. DOI: 10.1063/1.1683075.
(9) Ryckaert, J.-P.; Ciccotti, G.; Berendsen, H. J. C. Numerical Integration of the Cartesian Equations of Motion of a System with Constraints: Molecular Dynamics of n-Alkanes. *J Comput Phys* **1977**, *23*, 327-341.

7) Appendix 1. NMR Spectra

Compound APA- ^1H NMR in D_2O

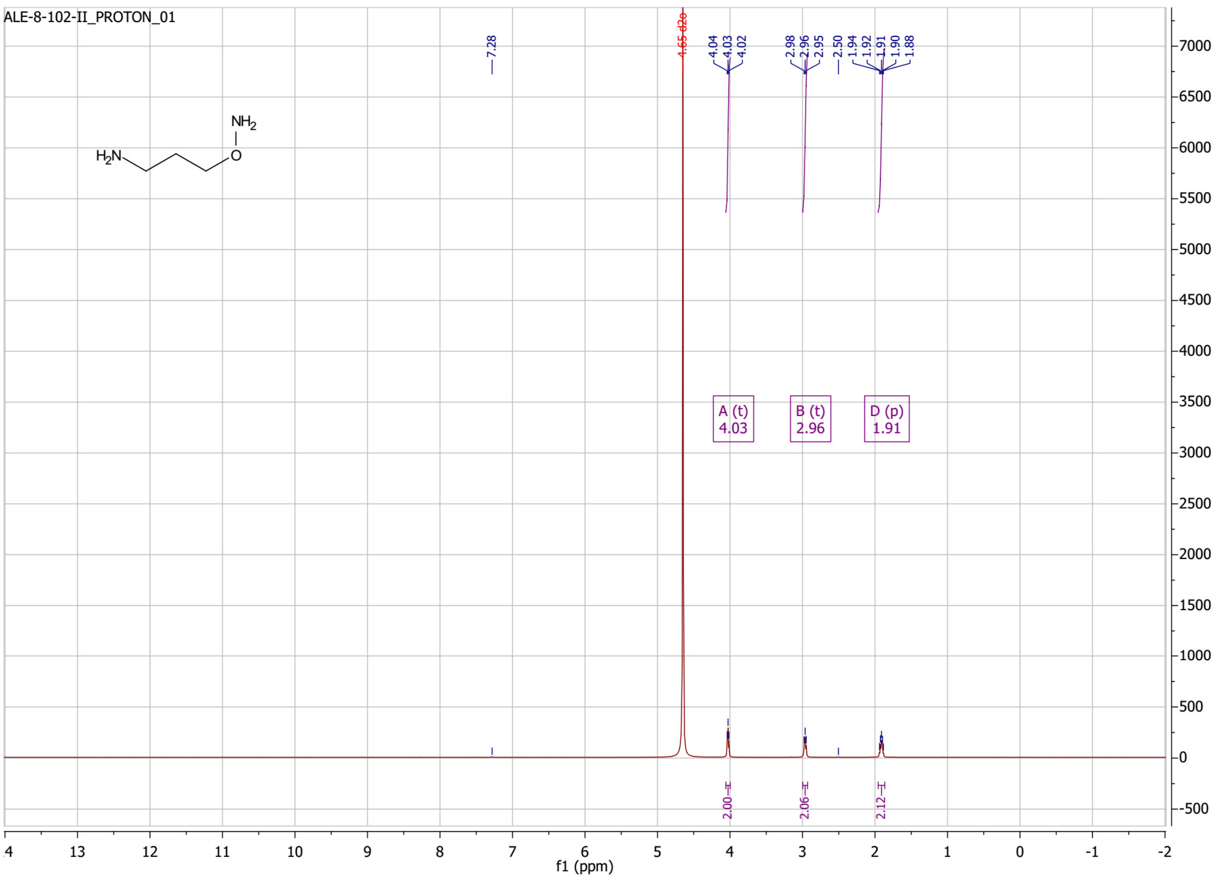

Compound APA- ^{13}C NMR in D_2O

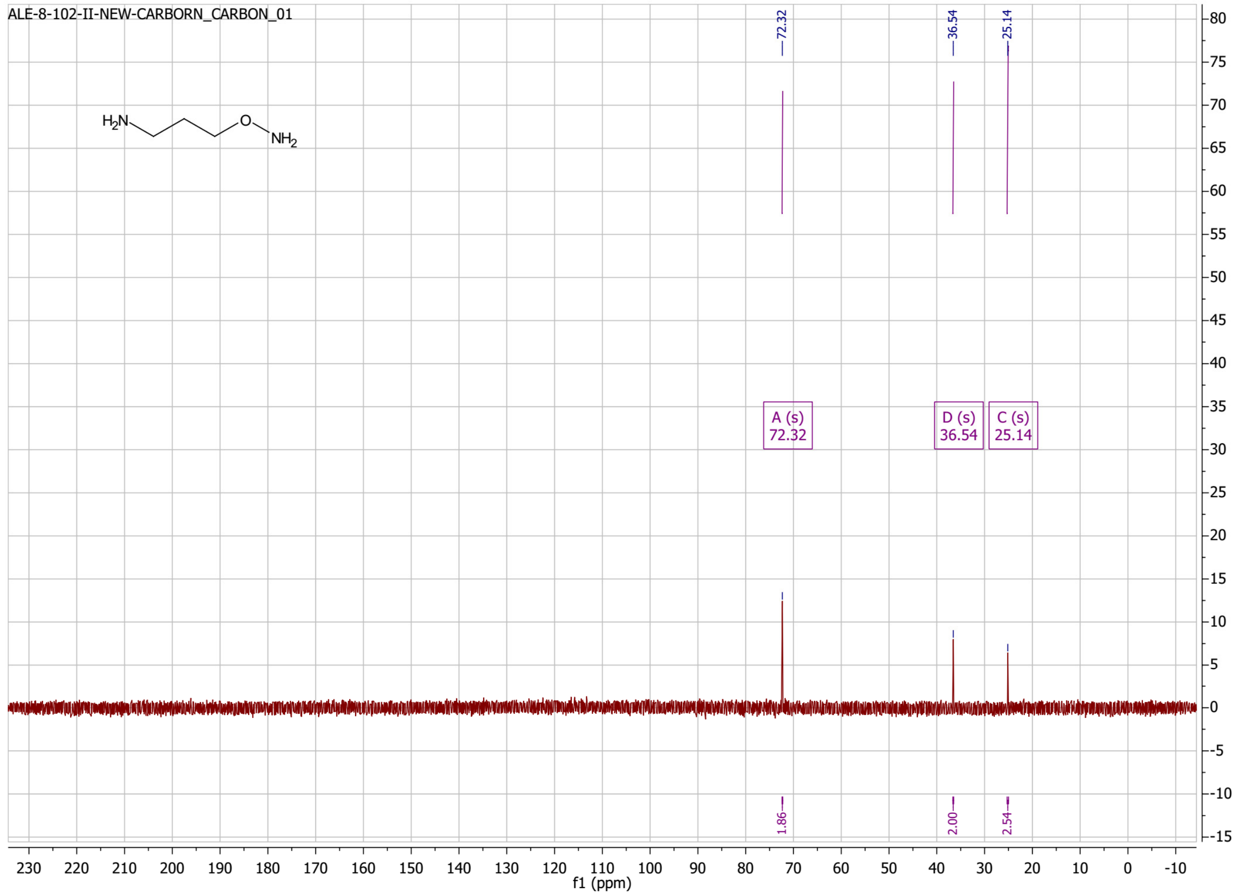

Compound APA-PLP- ^{1}H NMR in D_2O (E/Z ratio 96:4)

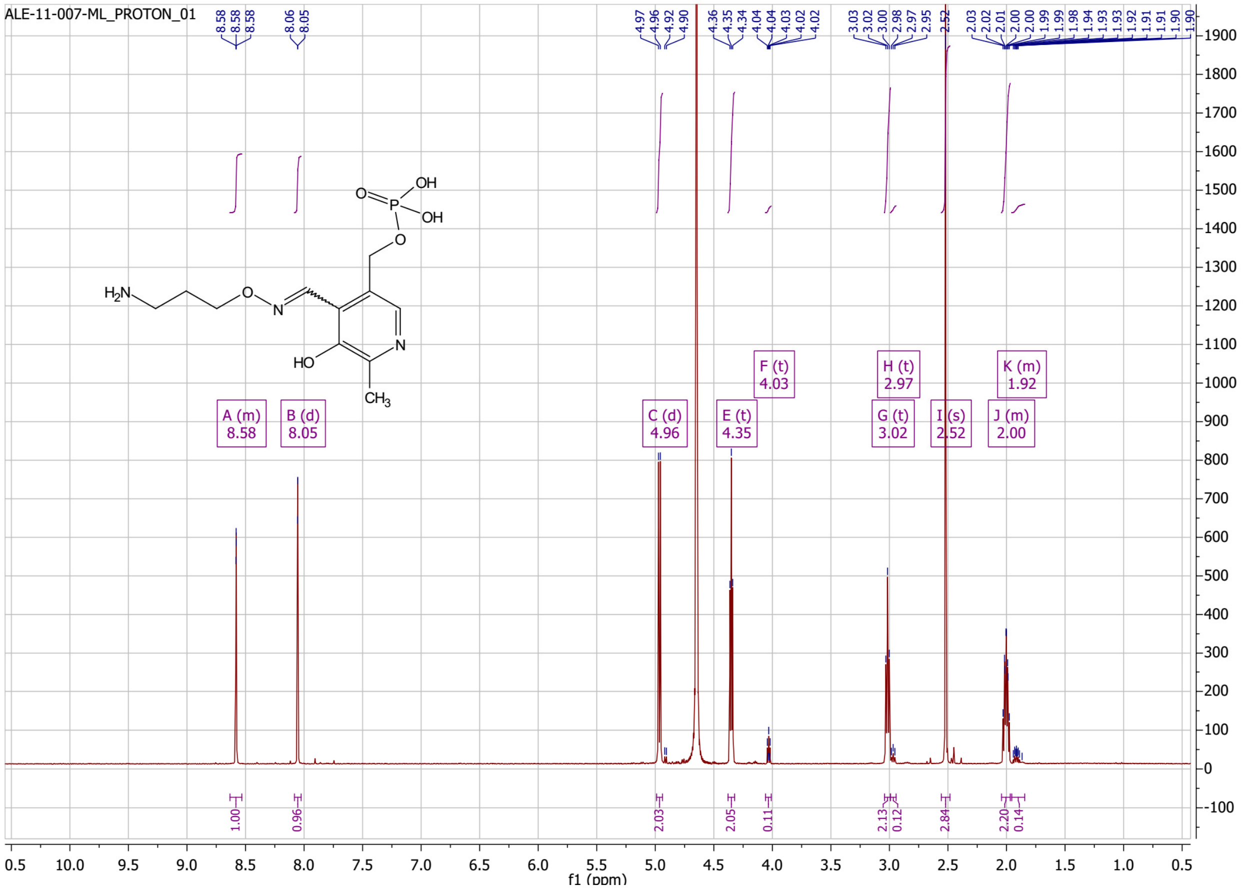

Compound APA-PLP- ^{13}C NMR in D_2O

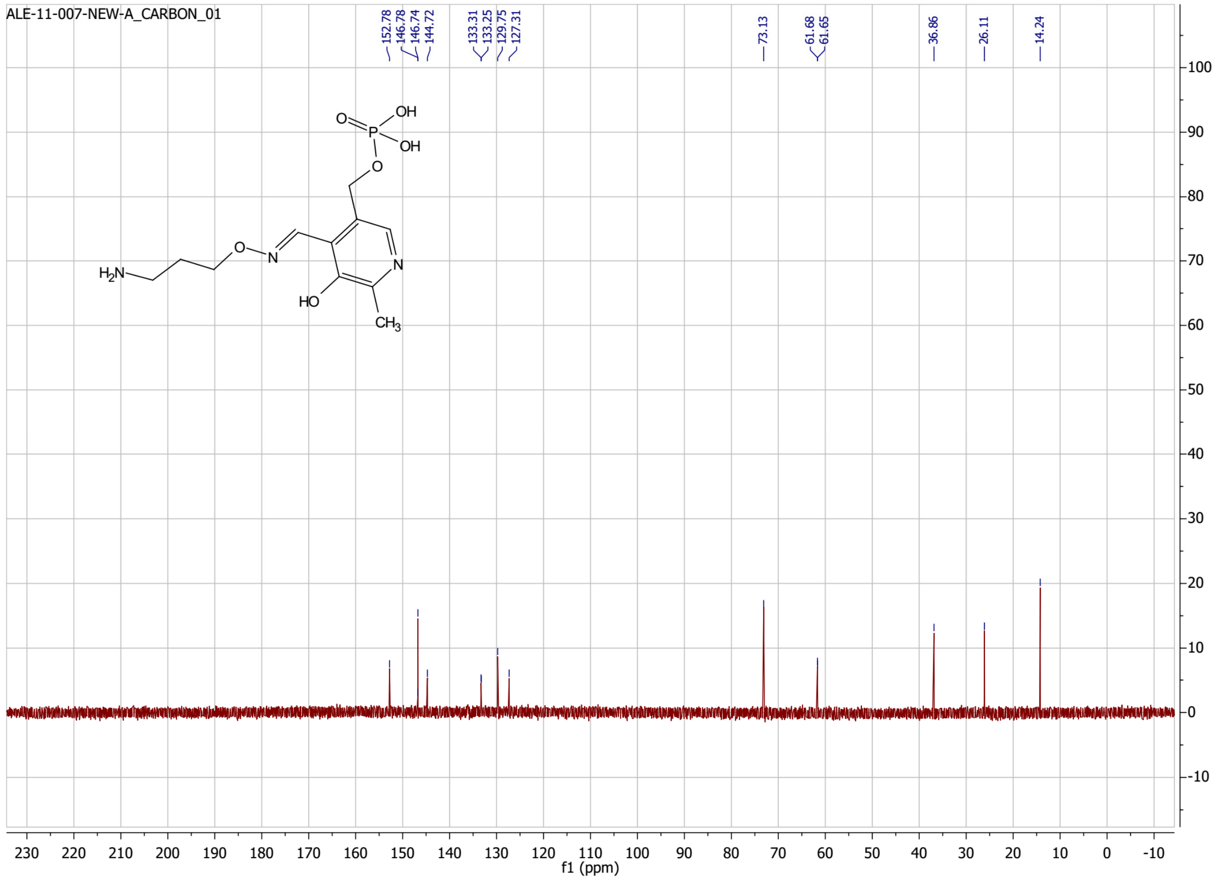

Compound 5- ^{1}H NMR in D_2O

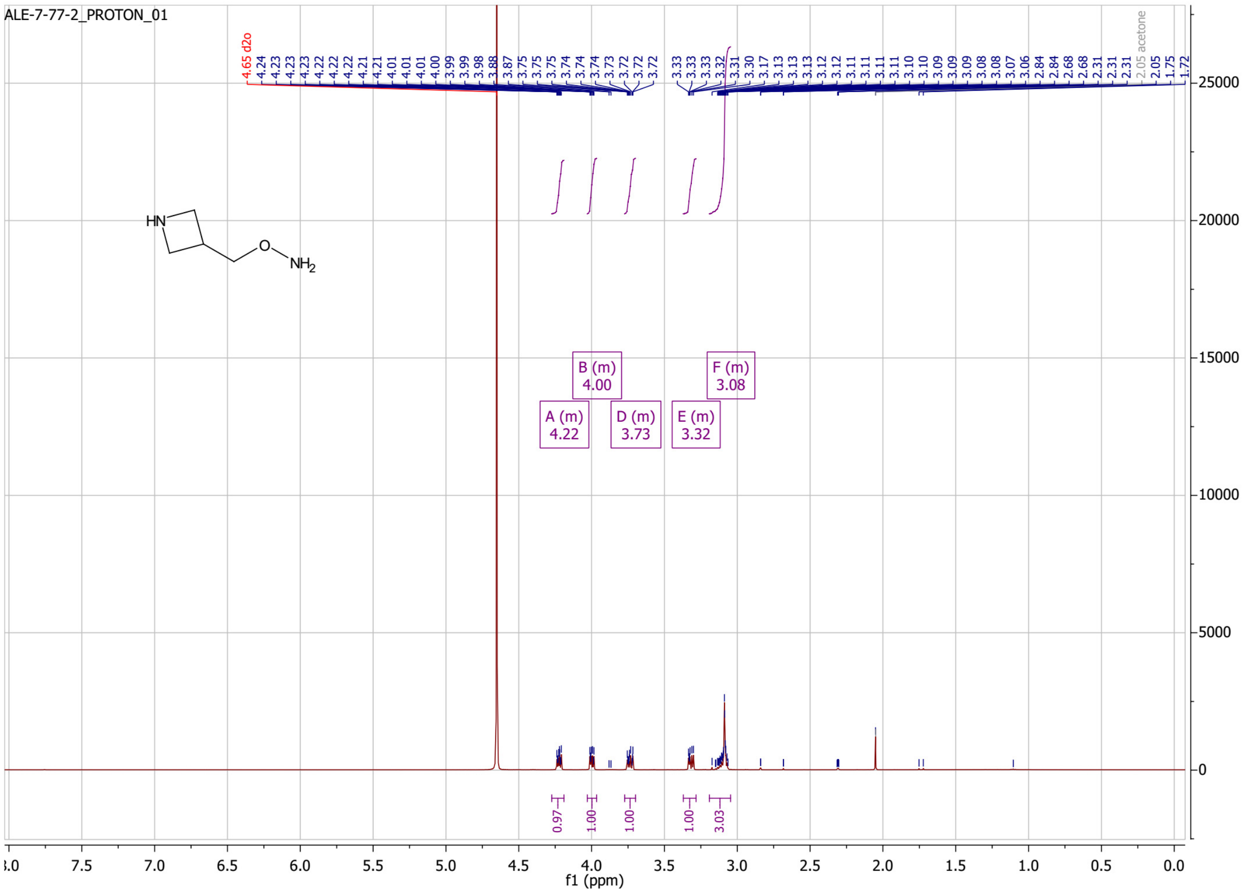

Compound 5- ^{13}C NMR in D_2O

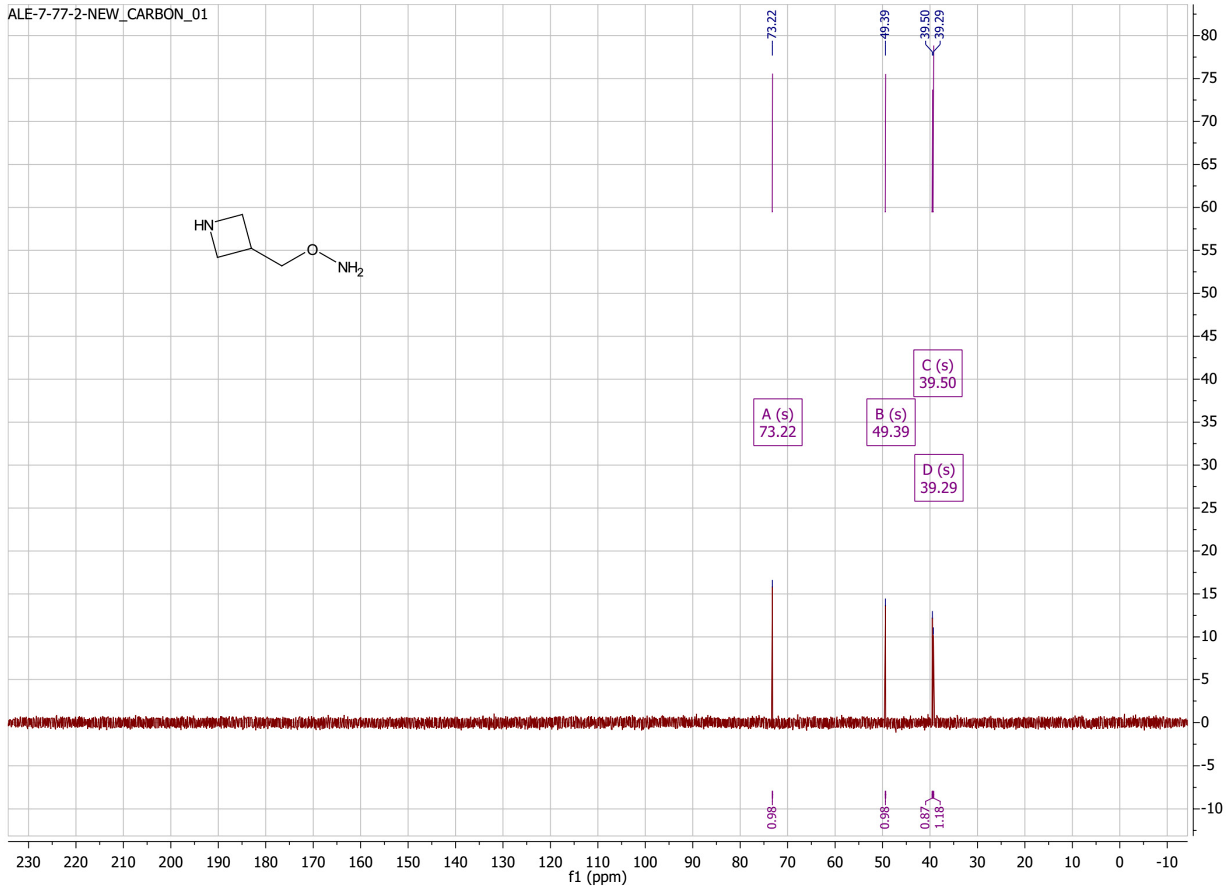

Compound 6- ^{1}H NMR in D_2O

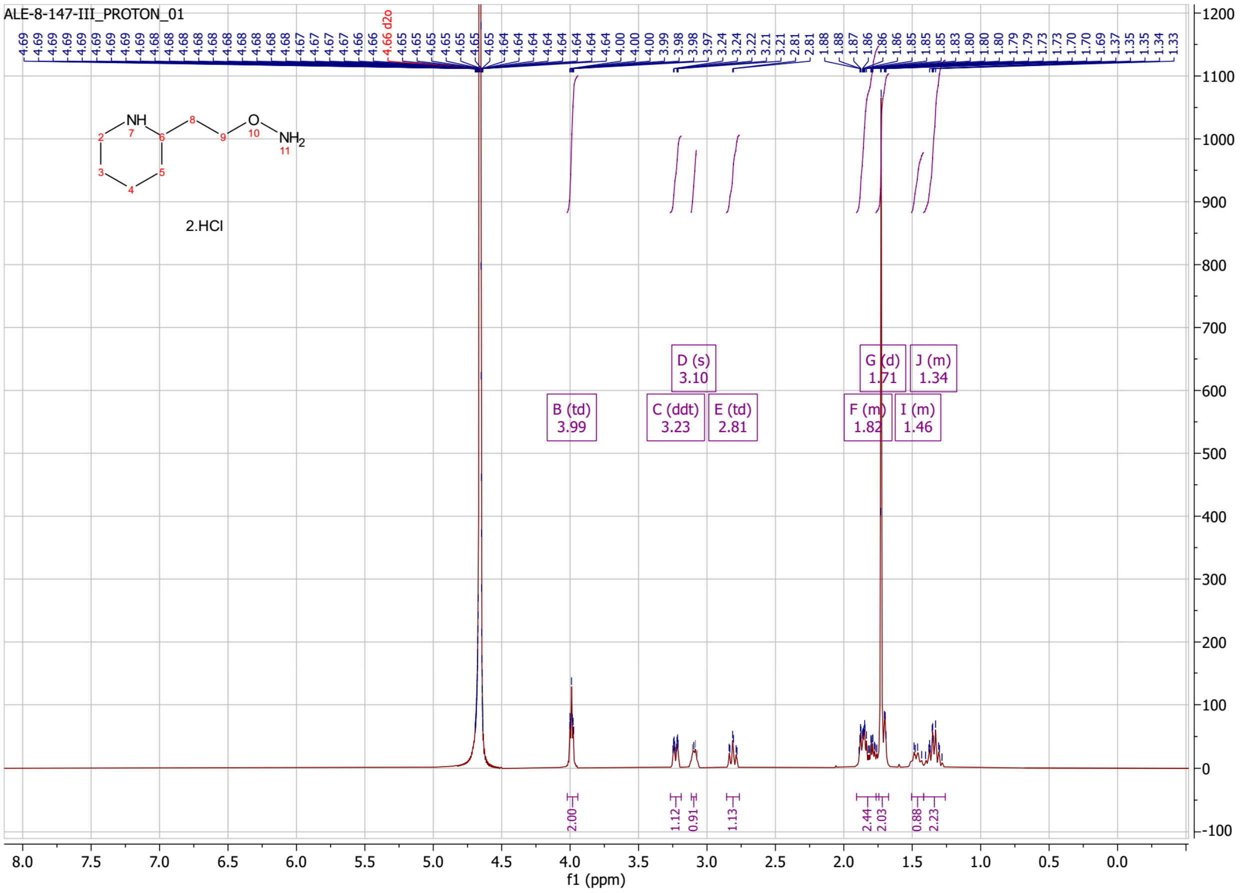

Compound 6- ^{13}C NMR in D_2O

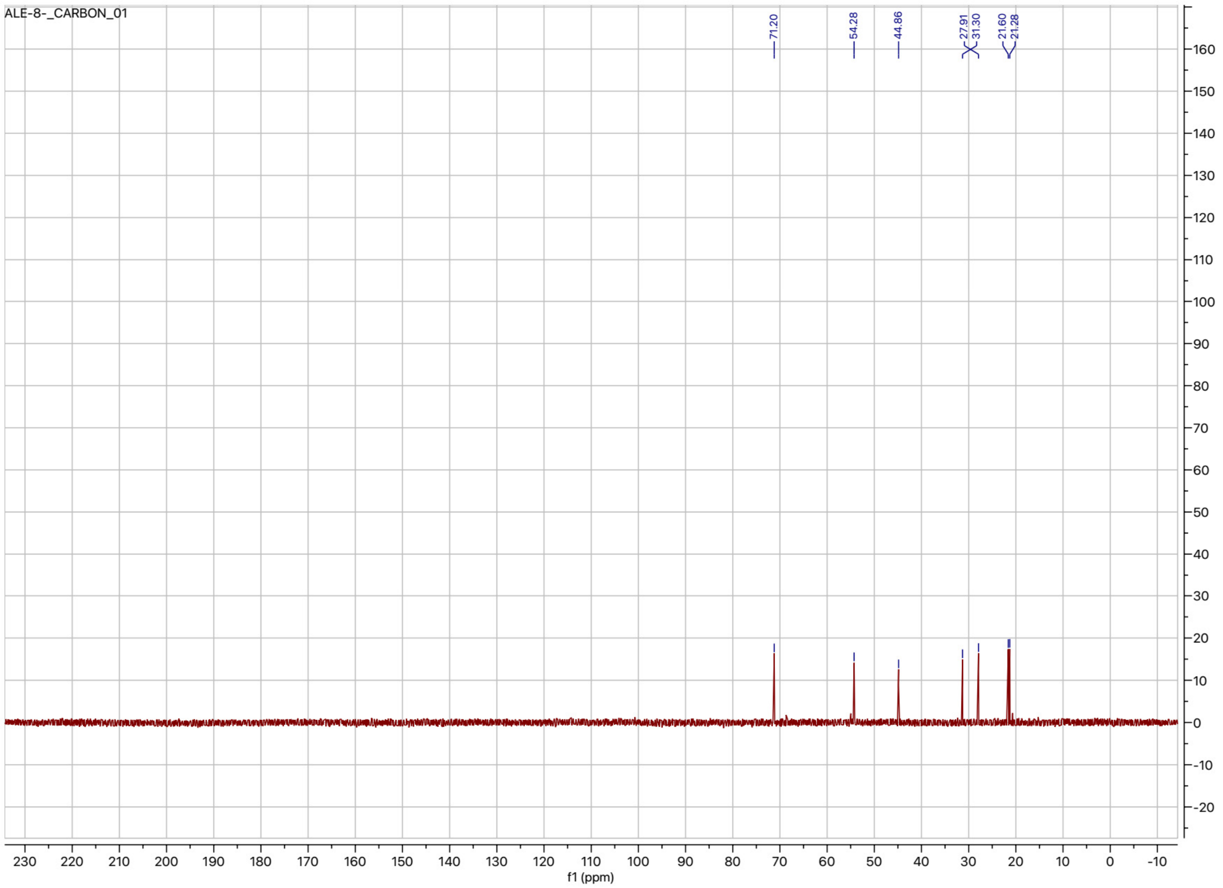

Compound 7- ^{1}H NMR in D_2O

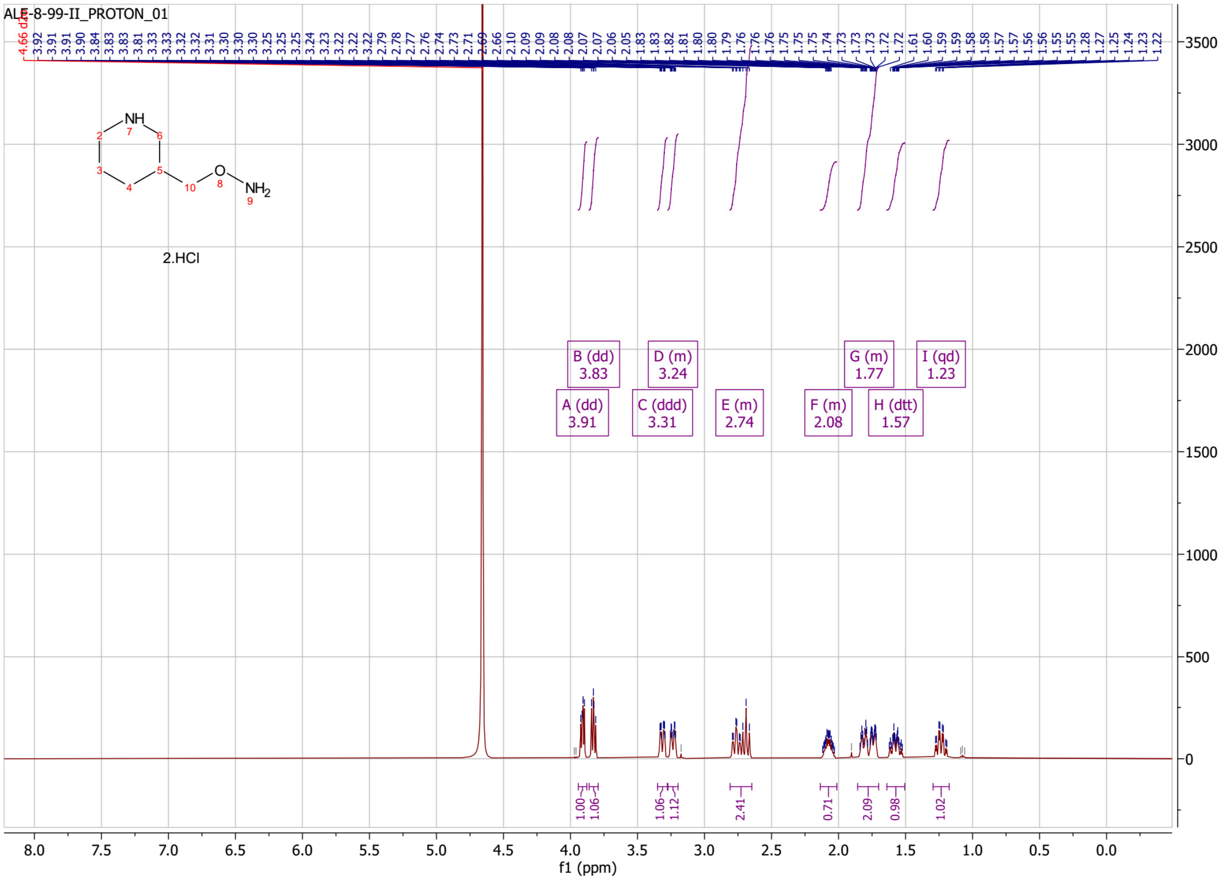

Compound 7- ^{13}C NMR in D_2O

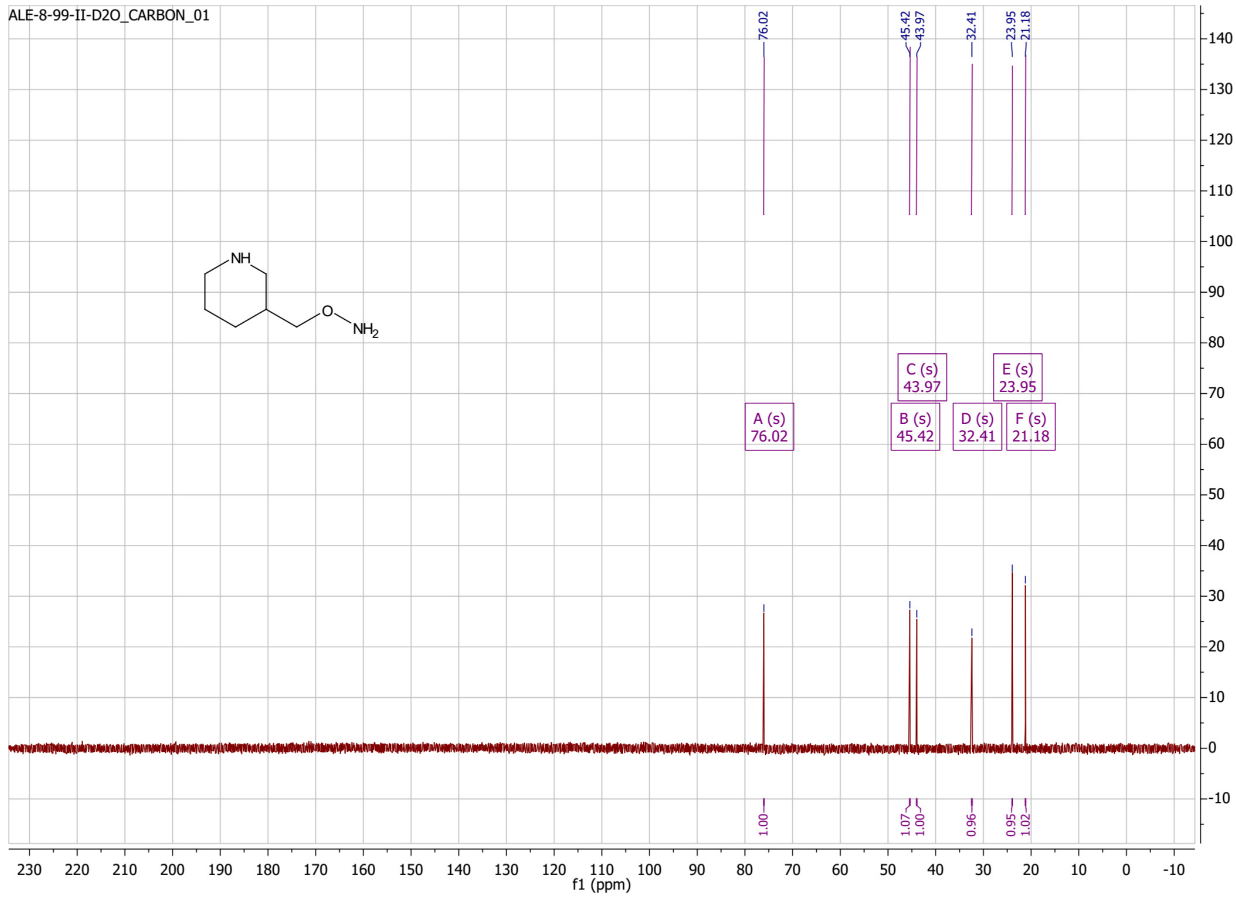

Compound 7R- ^{1}H NMR in D_2O

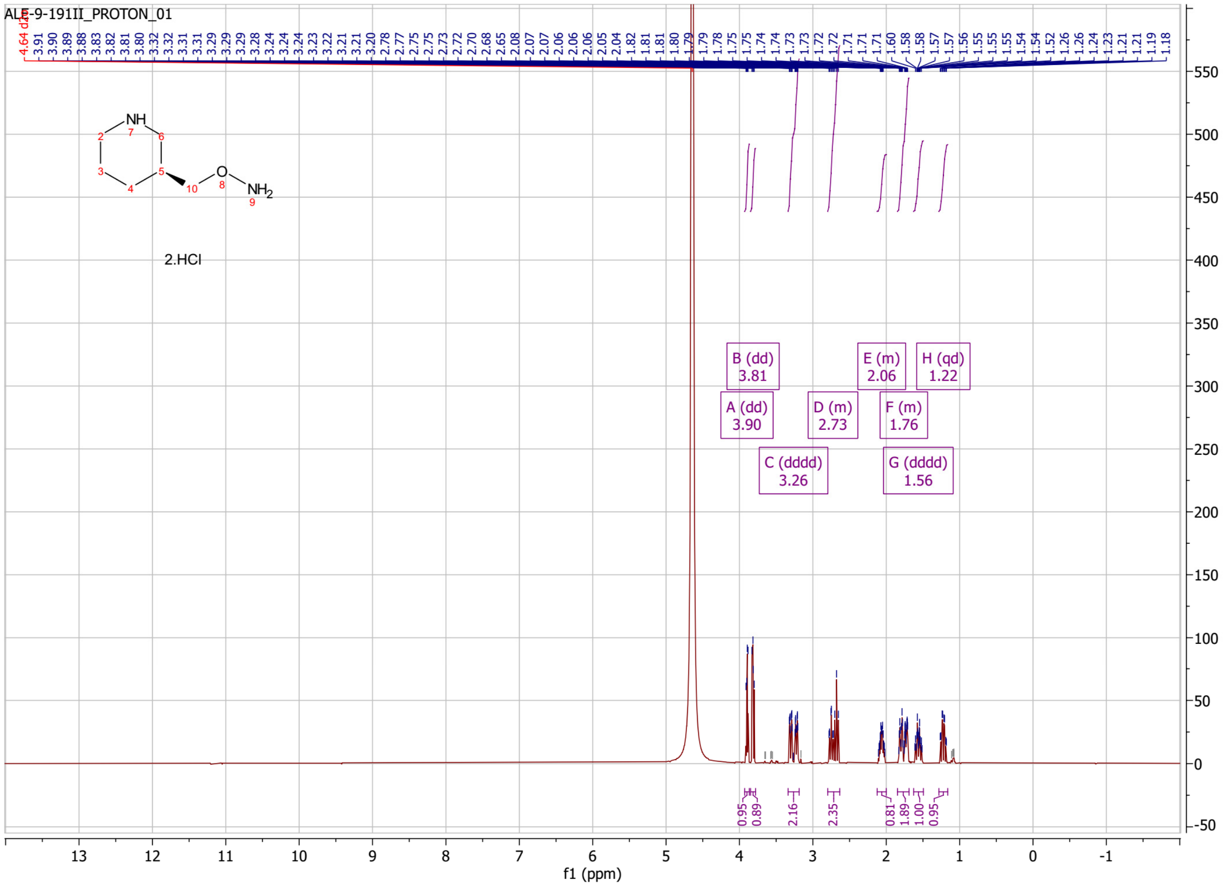

Compound 7R- ^{13}C NMR in D_2O

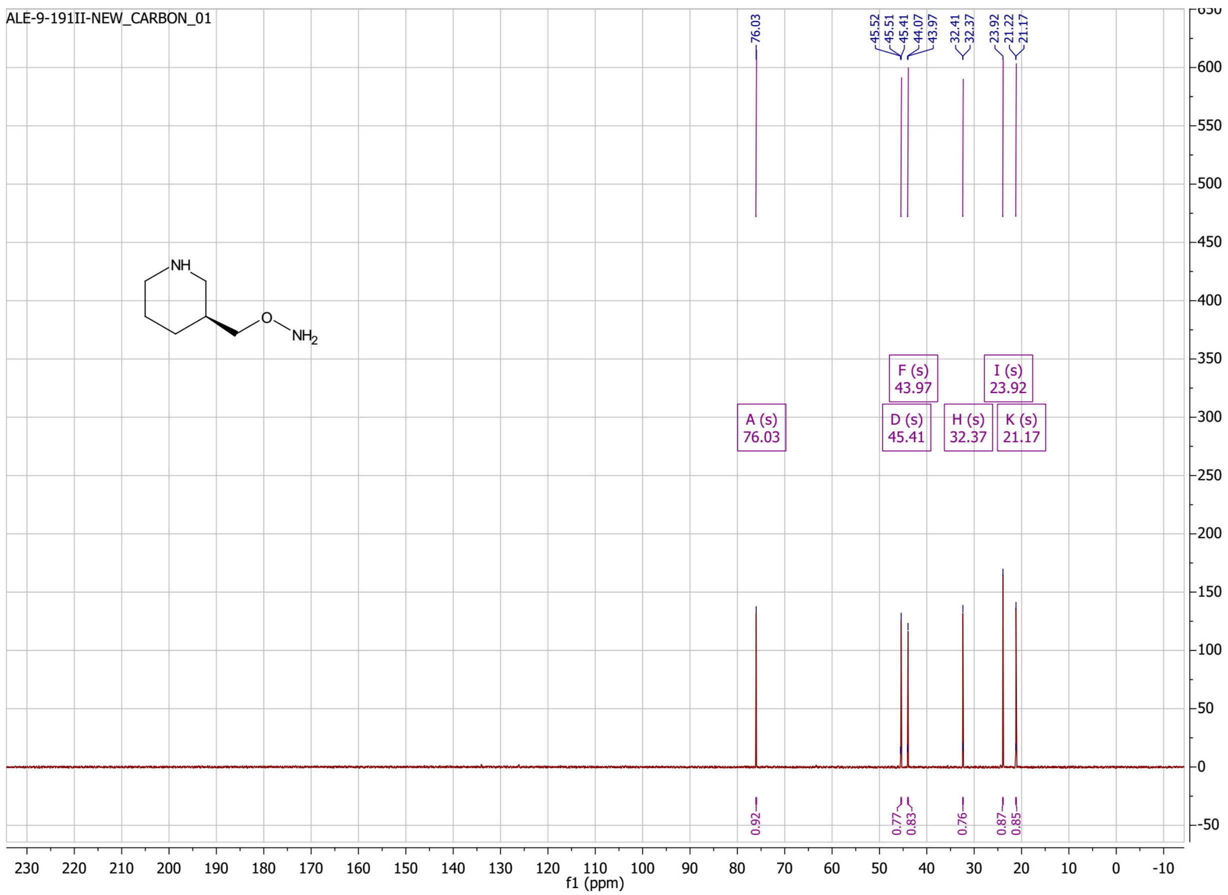

Compound 7S- ^{1}H NMR in D_2O

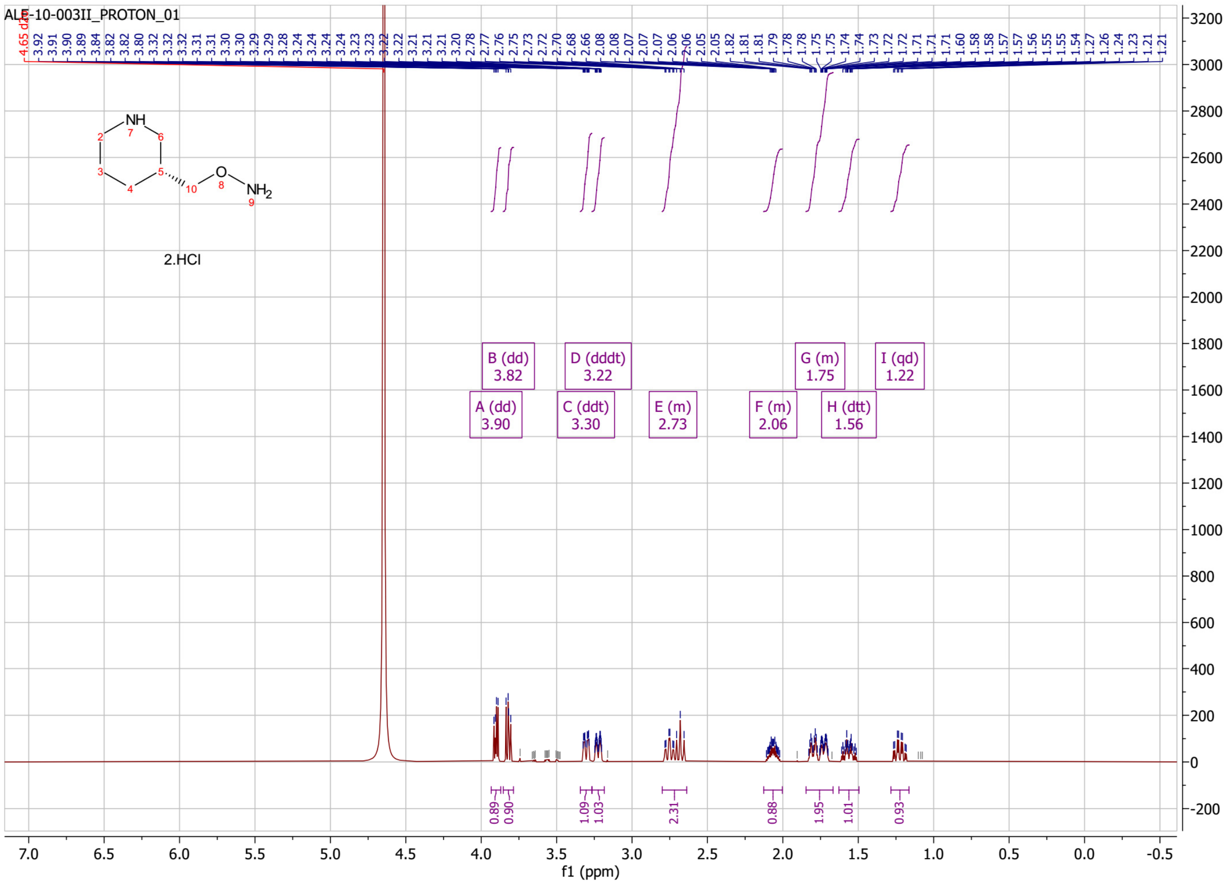

Compound 7S- ^{13}C NMR in D_2O

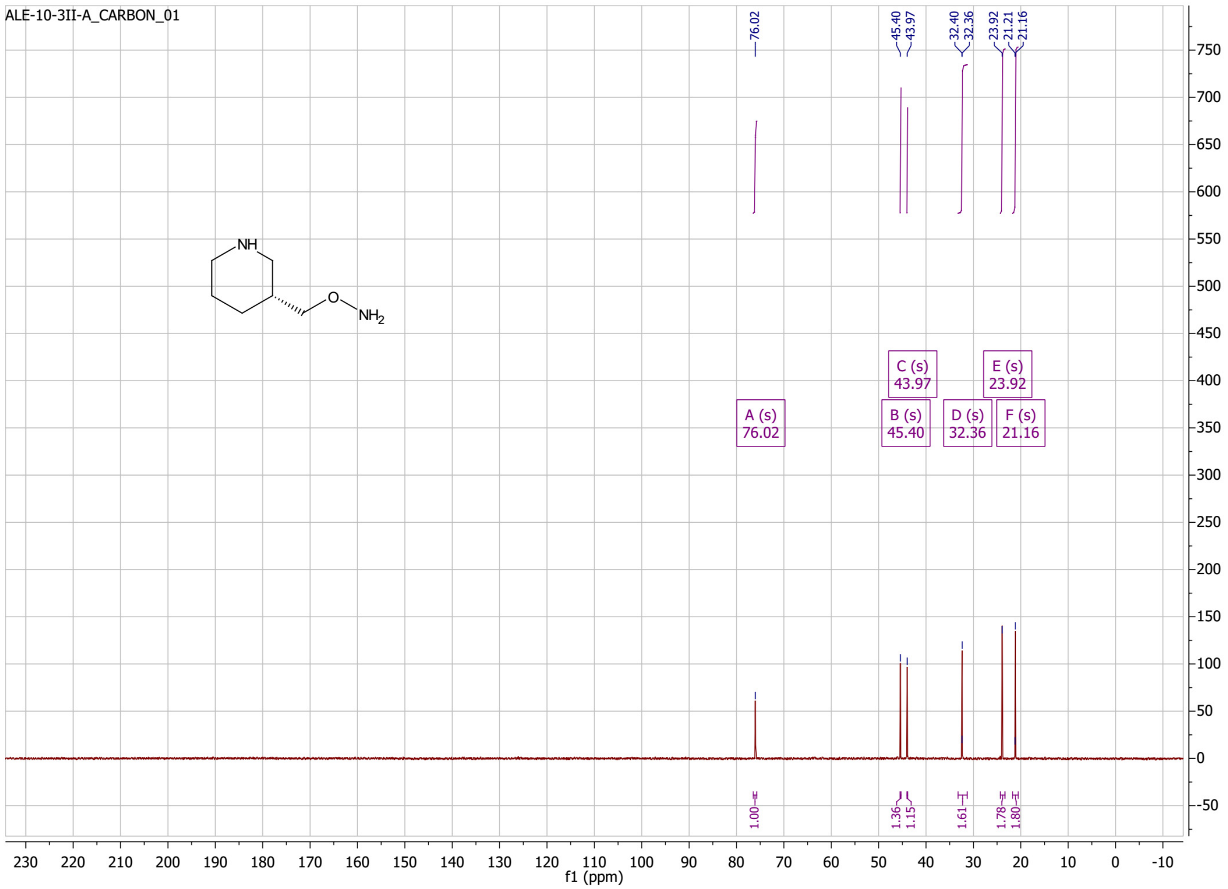

Compound 8- ^{1}H NMR in D_2O

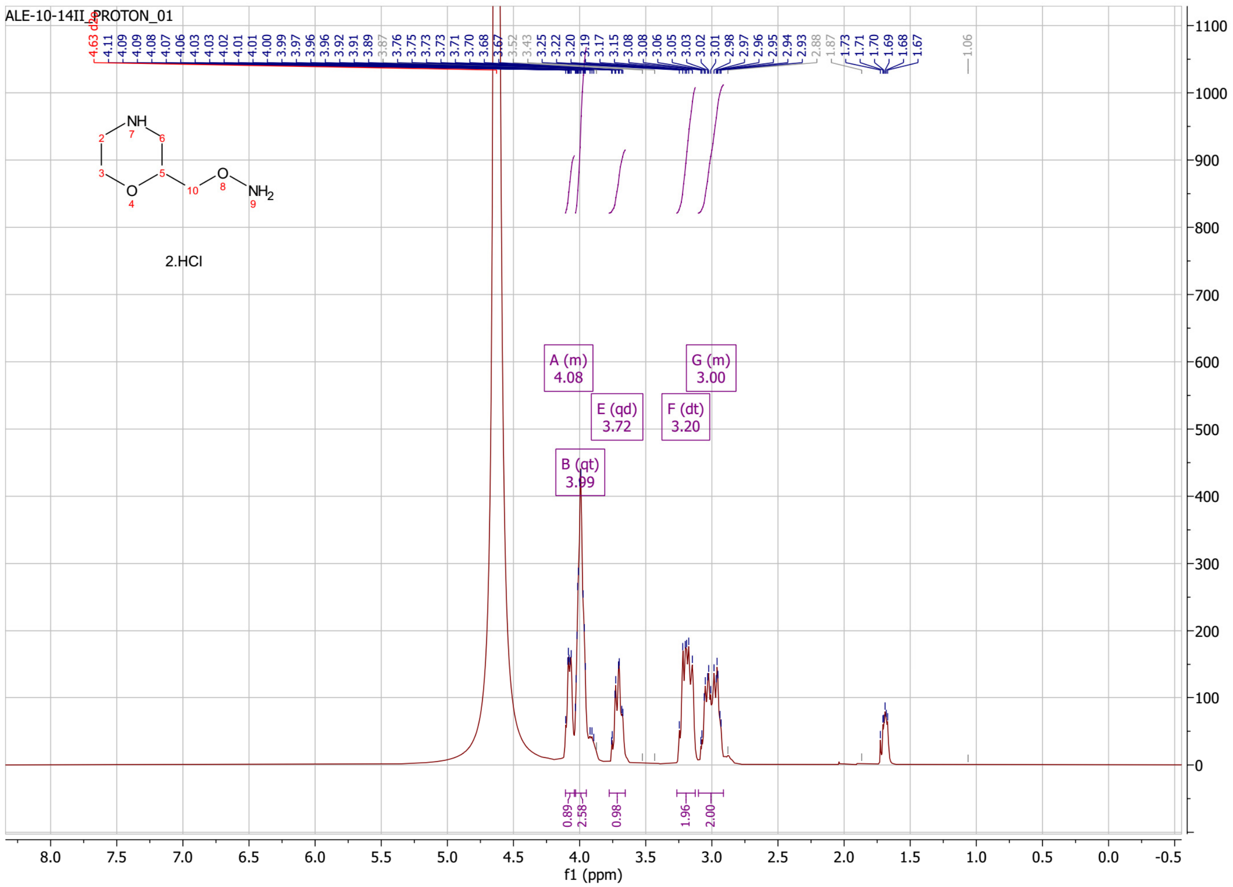

Compound 8- ^{13}C NMR in D_2O

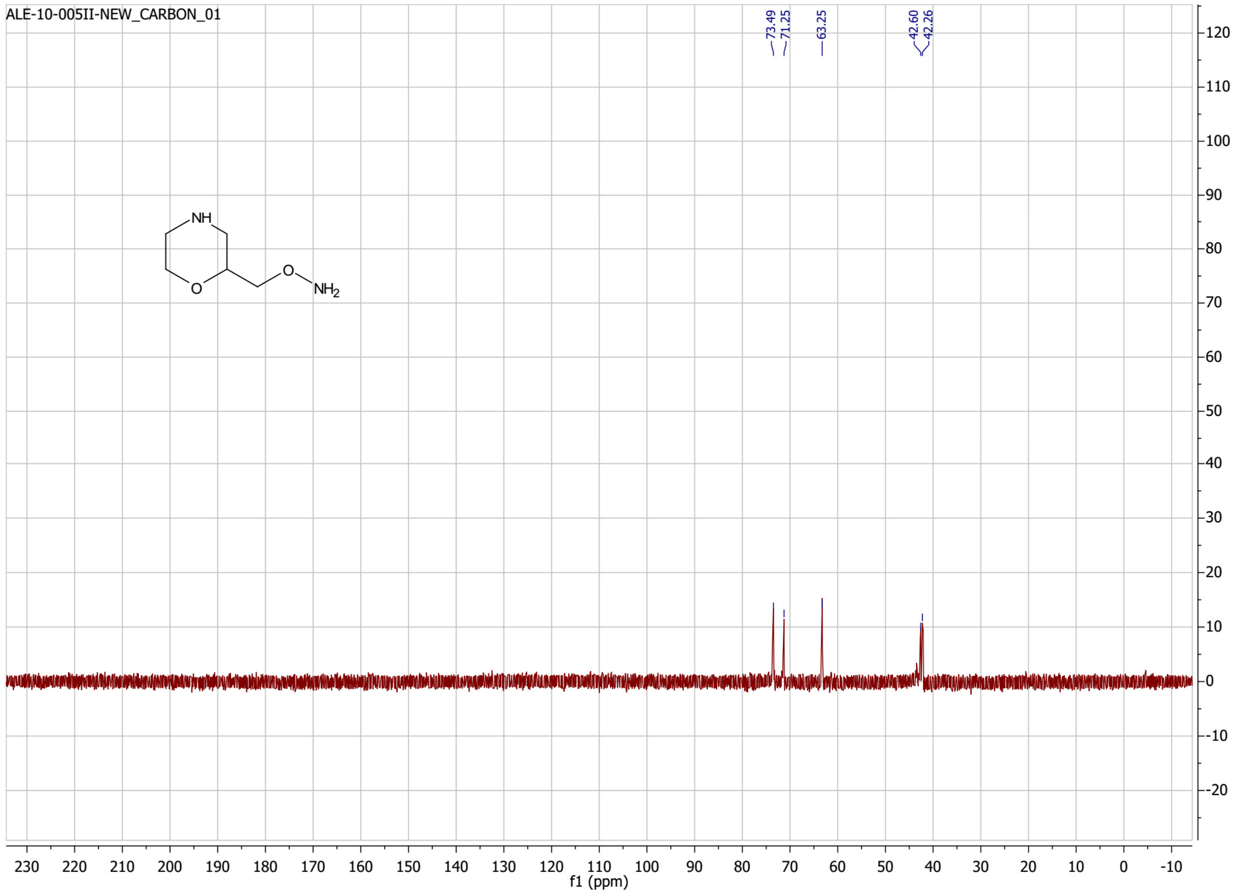

Compound 9- ^{1}H NMR in D_2O

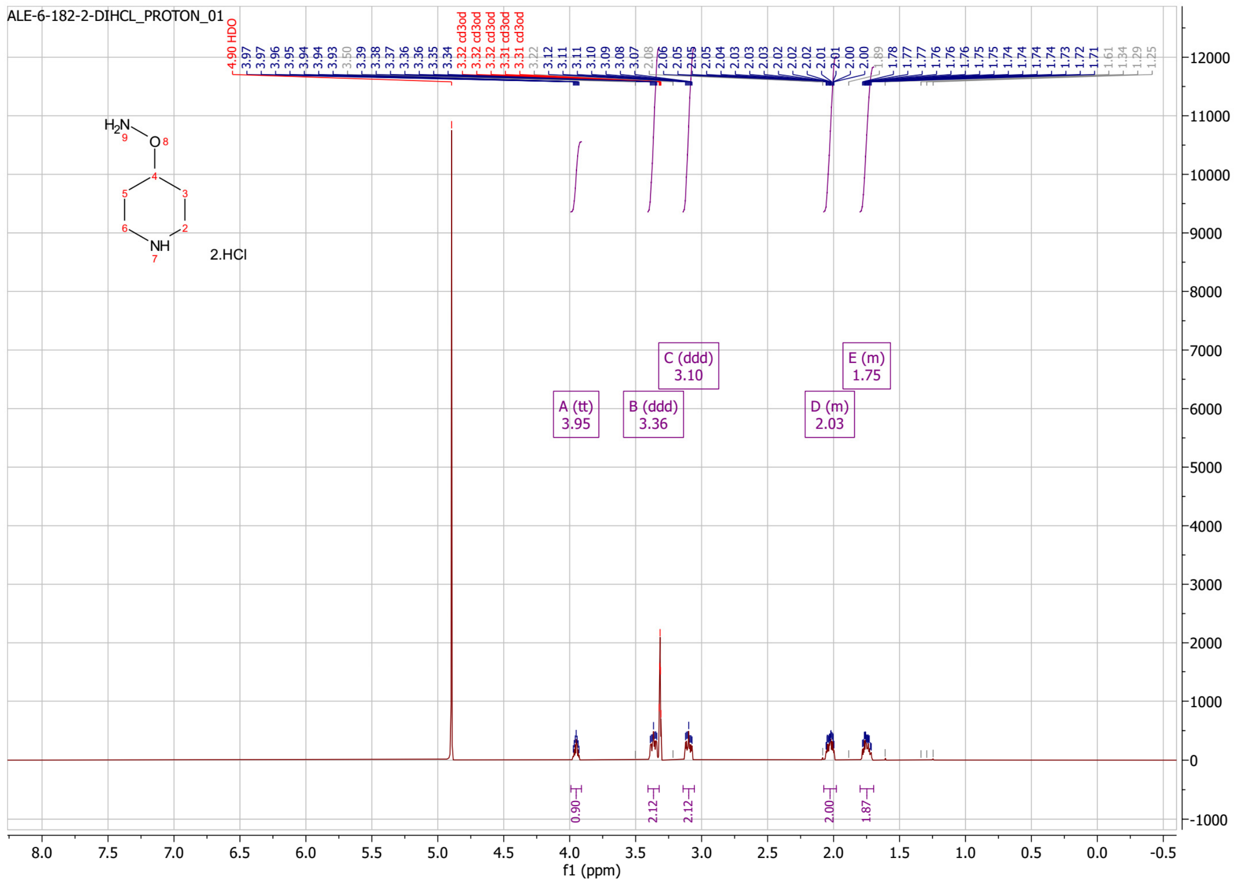

Compound 9- ^{13}C NMR in D_2O

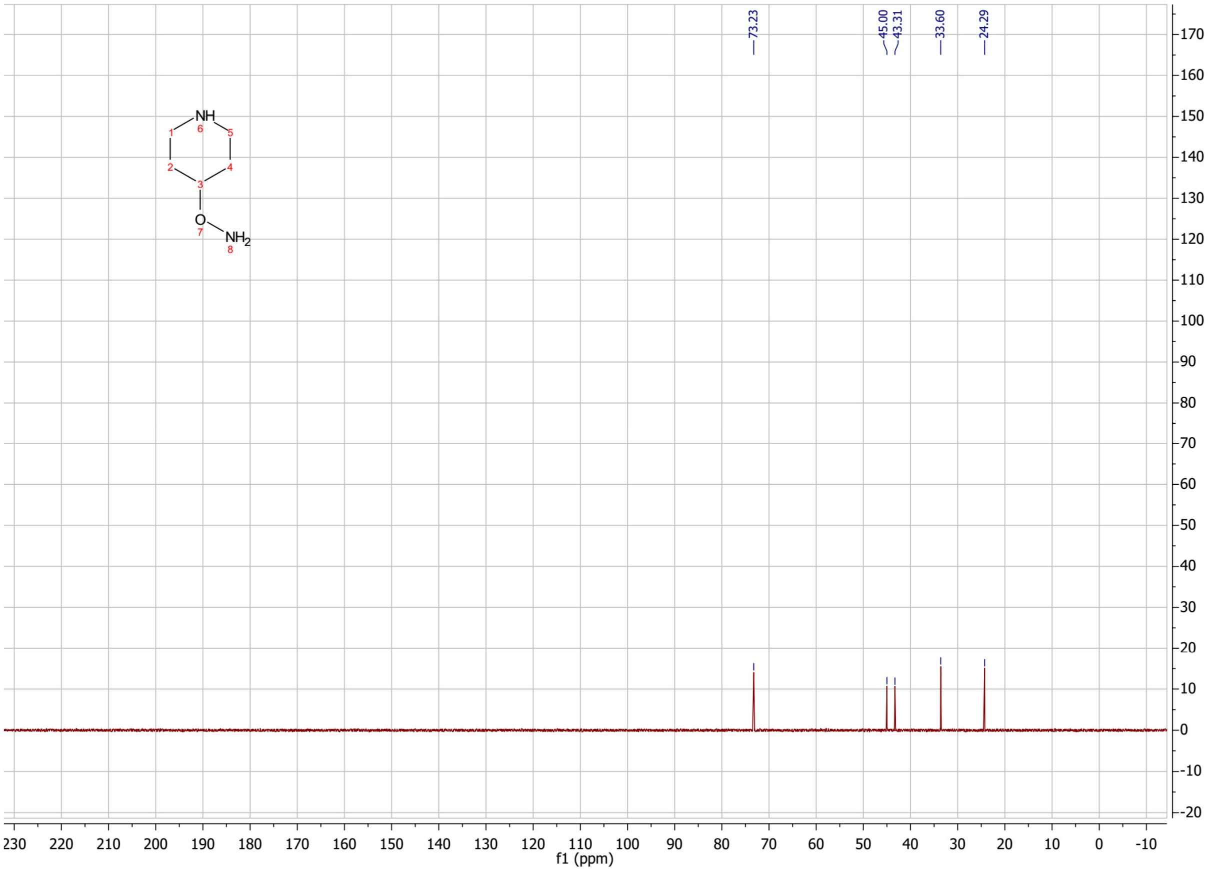

Compound 10- ^{1}H NMR in D_2O

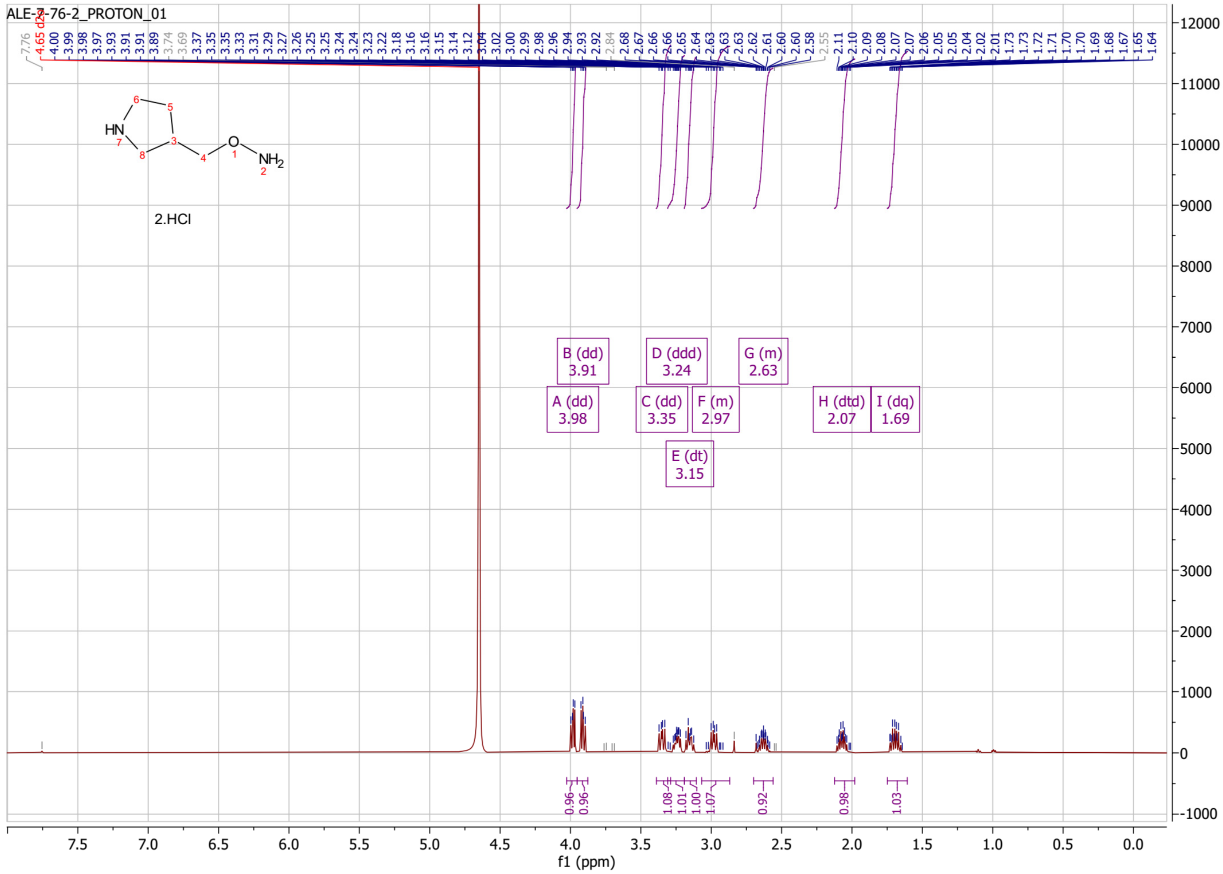

Compound 10- ^{13}C NMR in D_2O

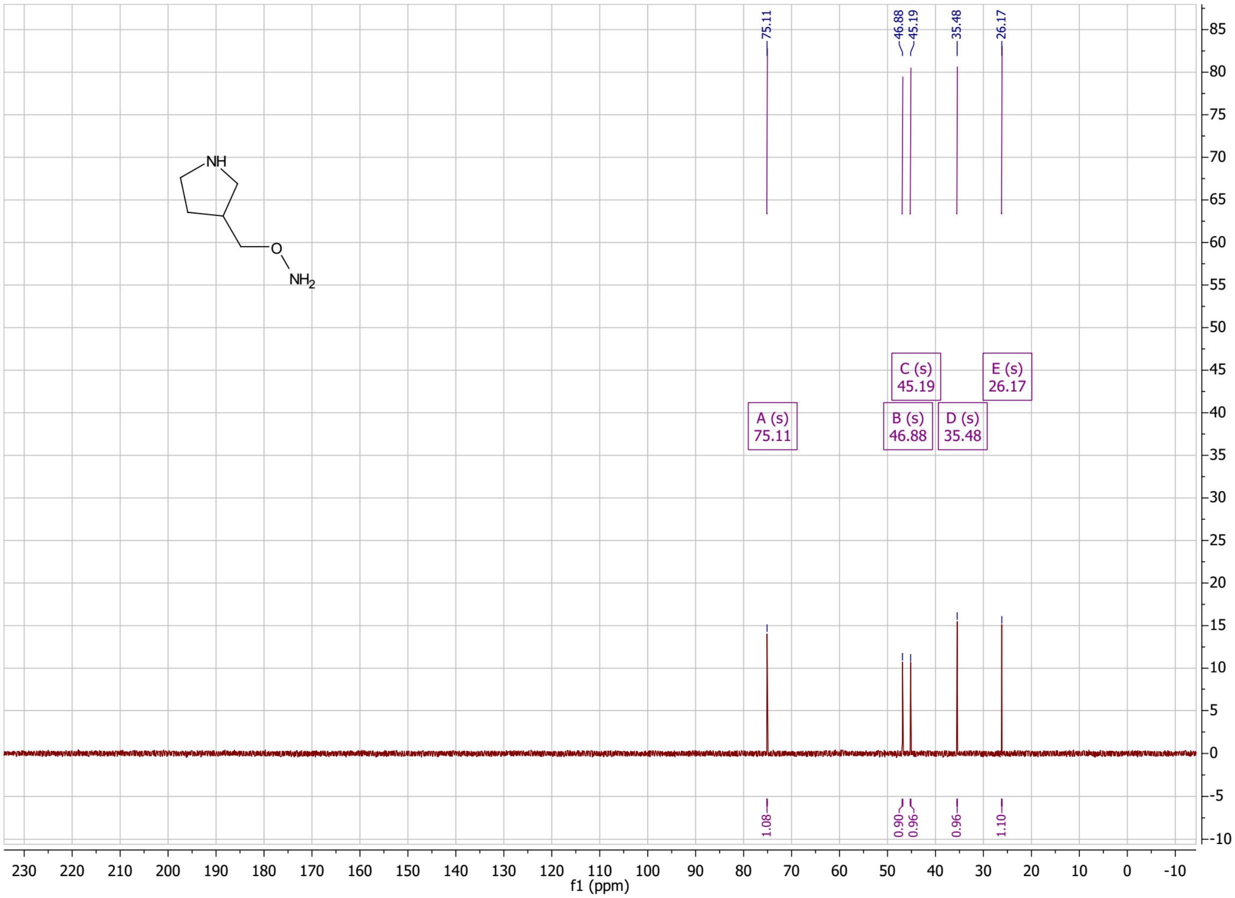

Compound 10*R*- ^{1}H NMR in D_2O

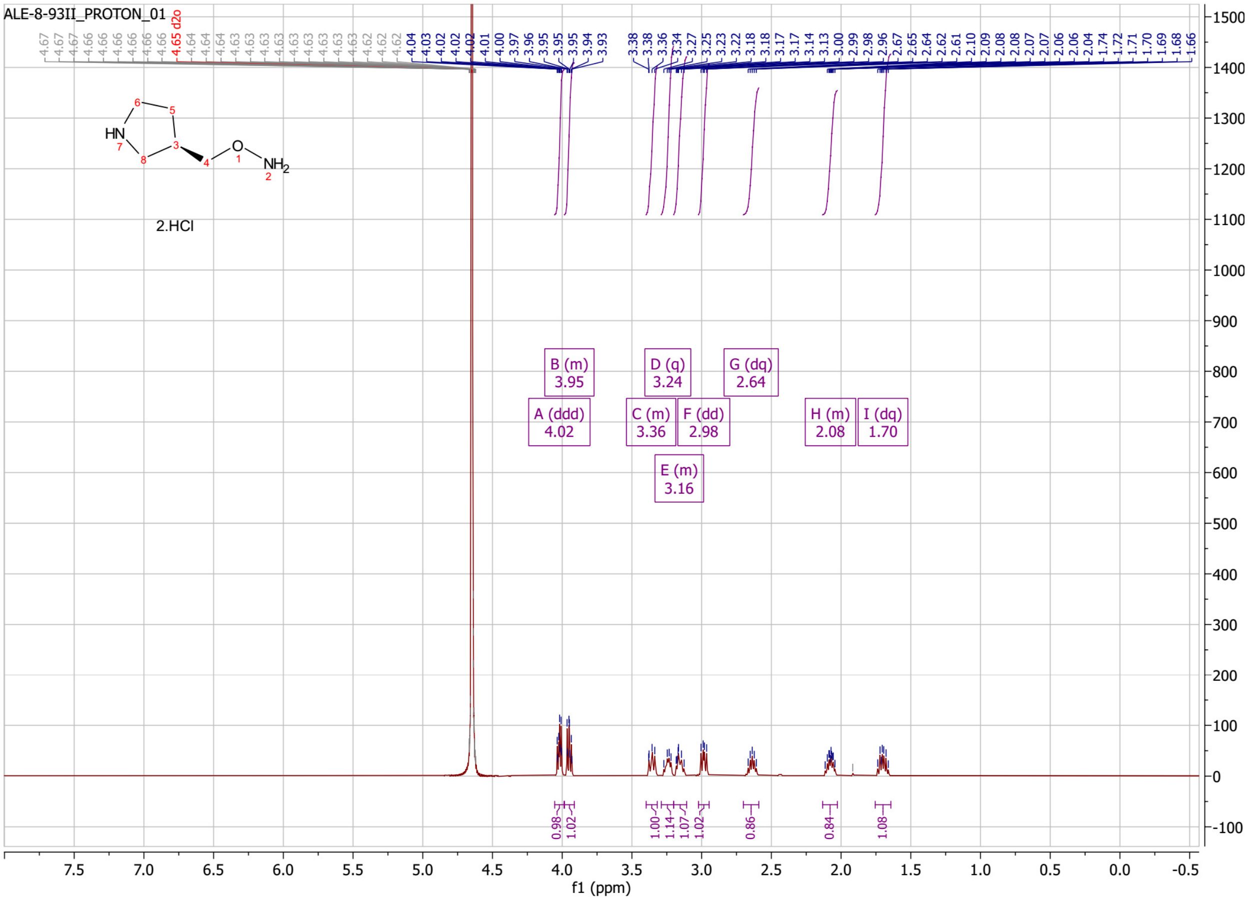

Compound 10*R*- ^{13}C NMR in D_2O

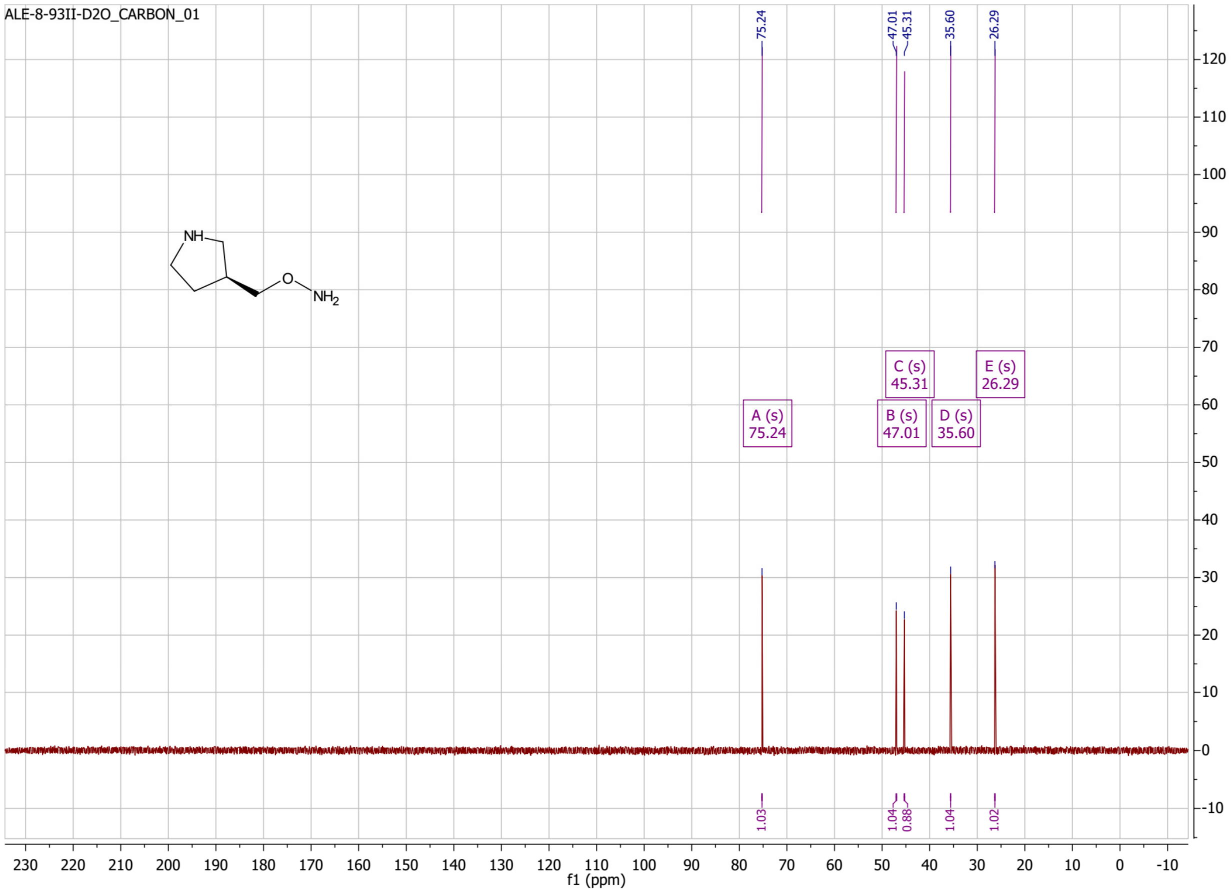

Compound 10*R*-PLP- ^{1}H NMR in D_2O

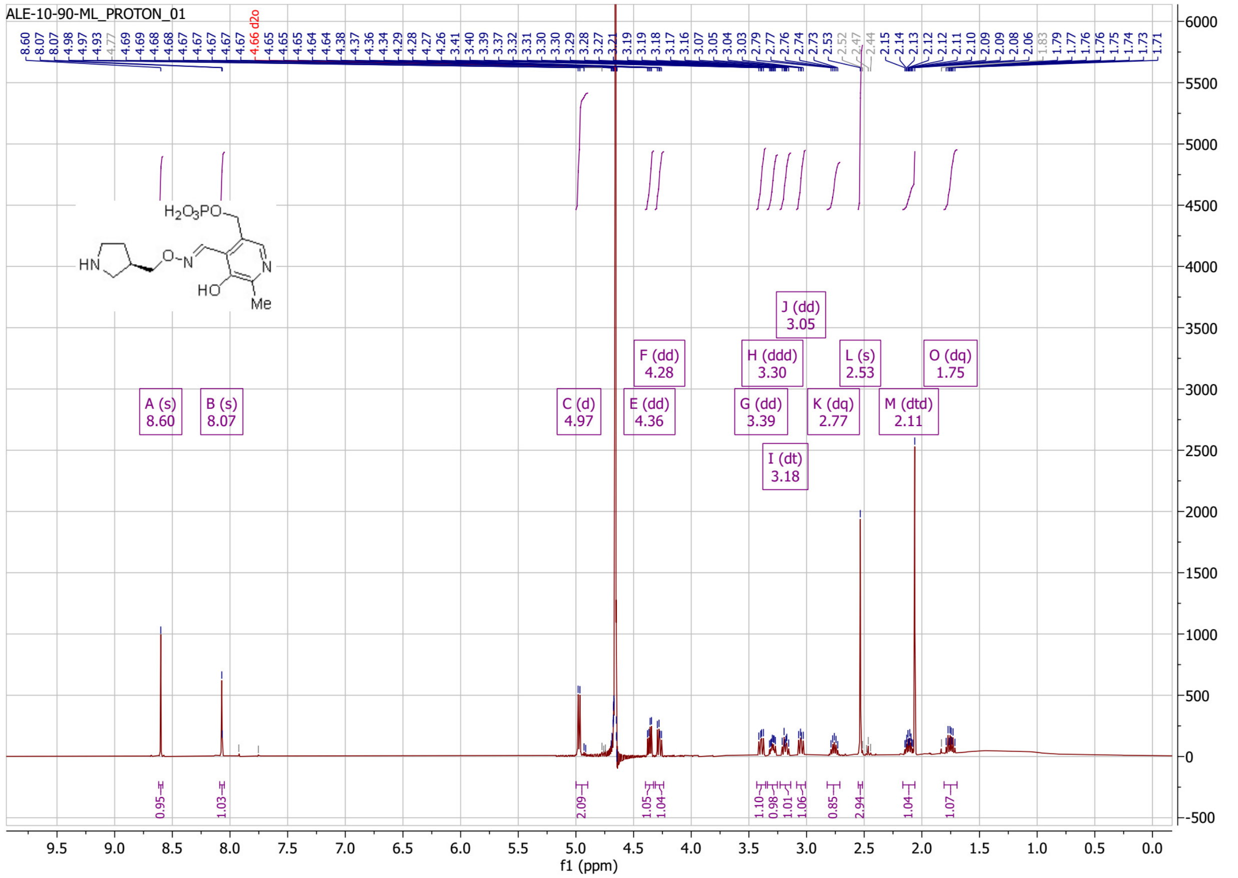

Compound 10*R*-PLP- ^{13}C NMR in D_2O

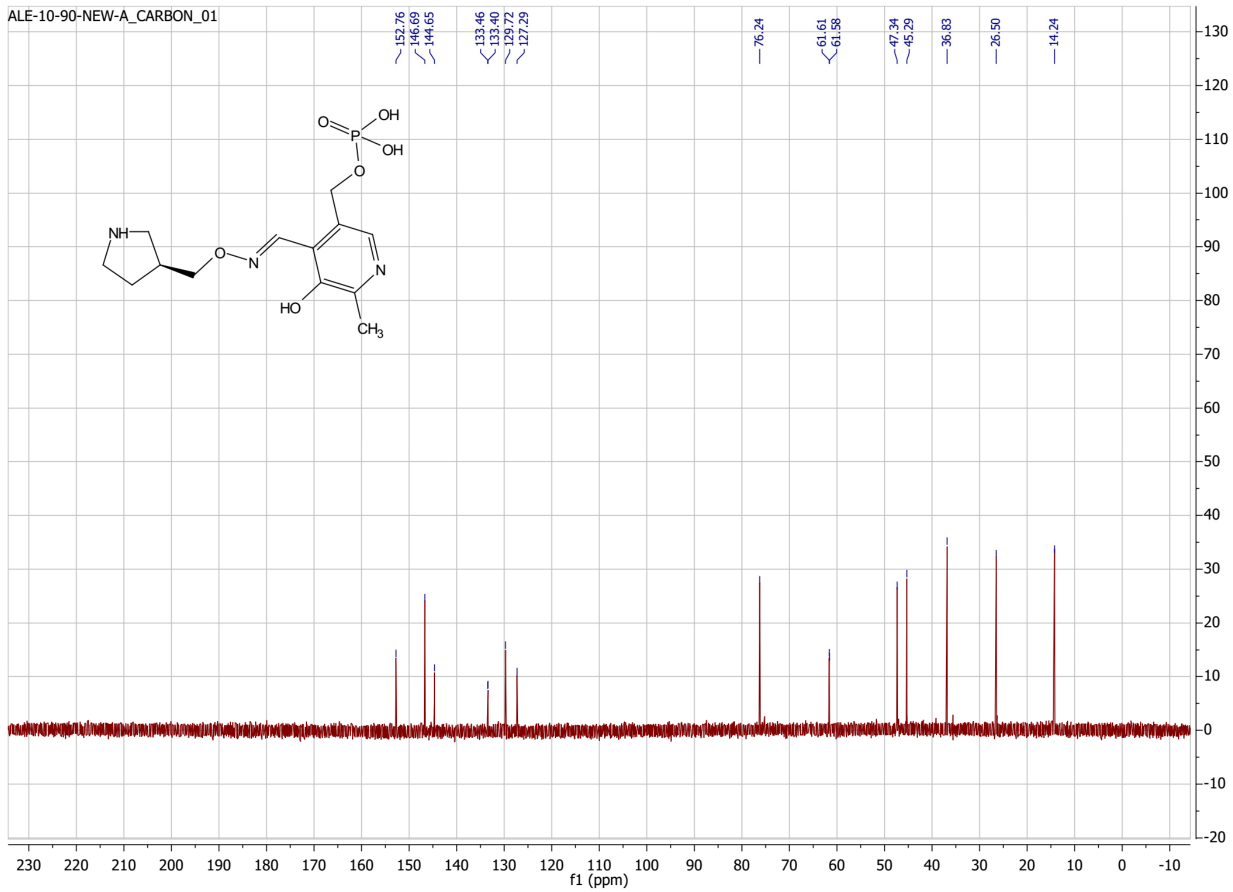

Compound 10*S*- ^{1}H NMR in D_2O

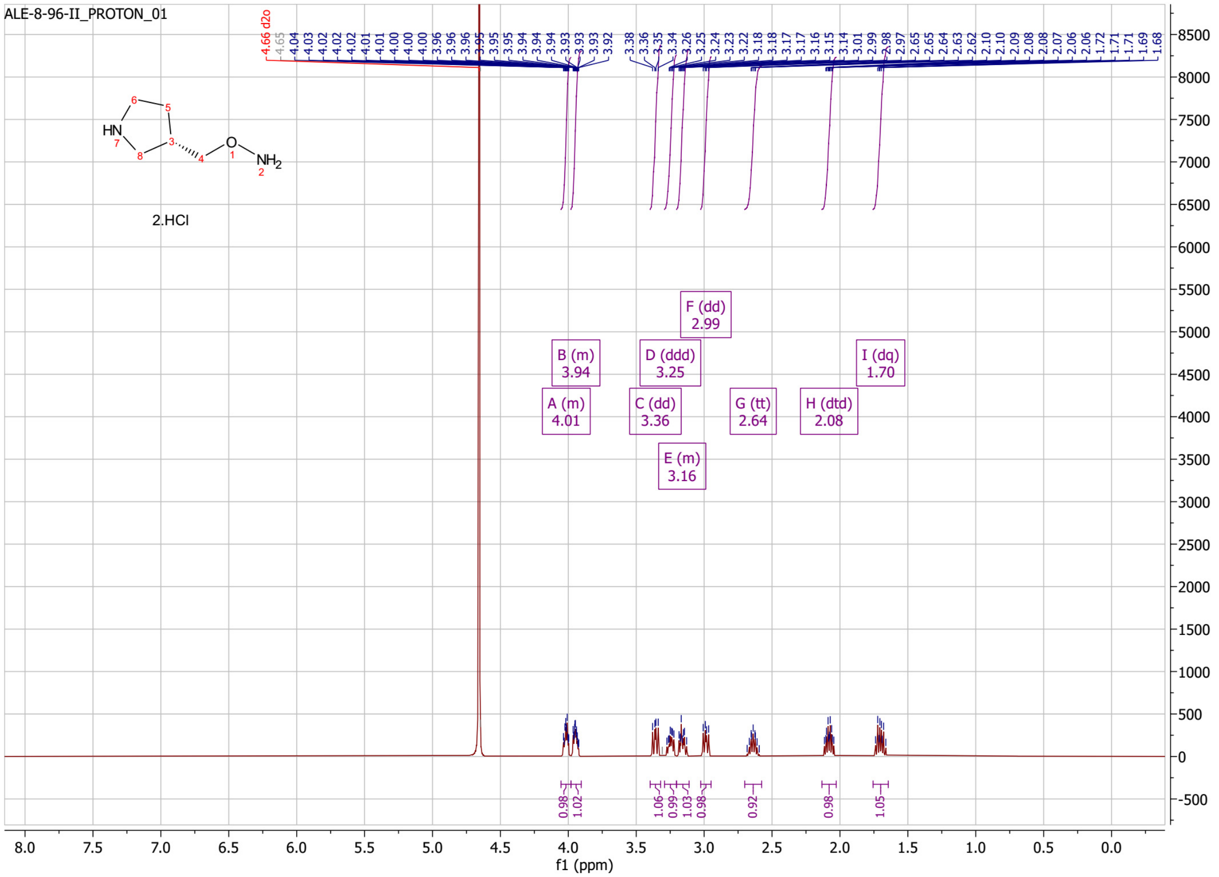

Compound 10*S*- ^{13}C NMR in D_2O

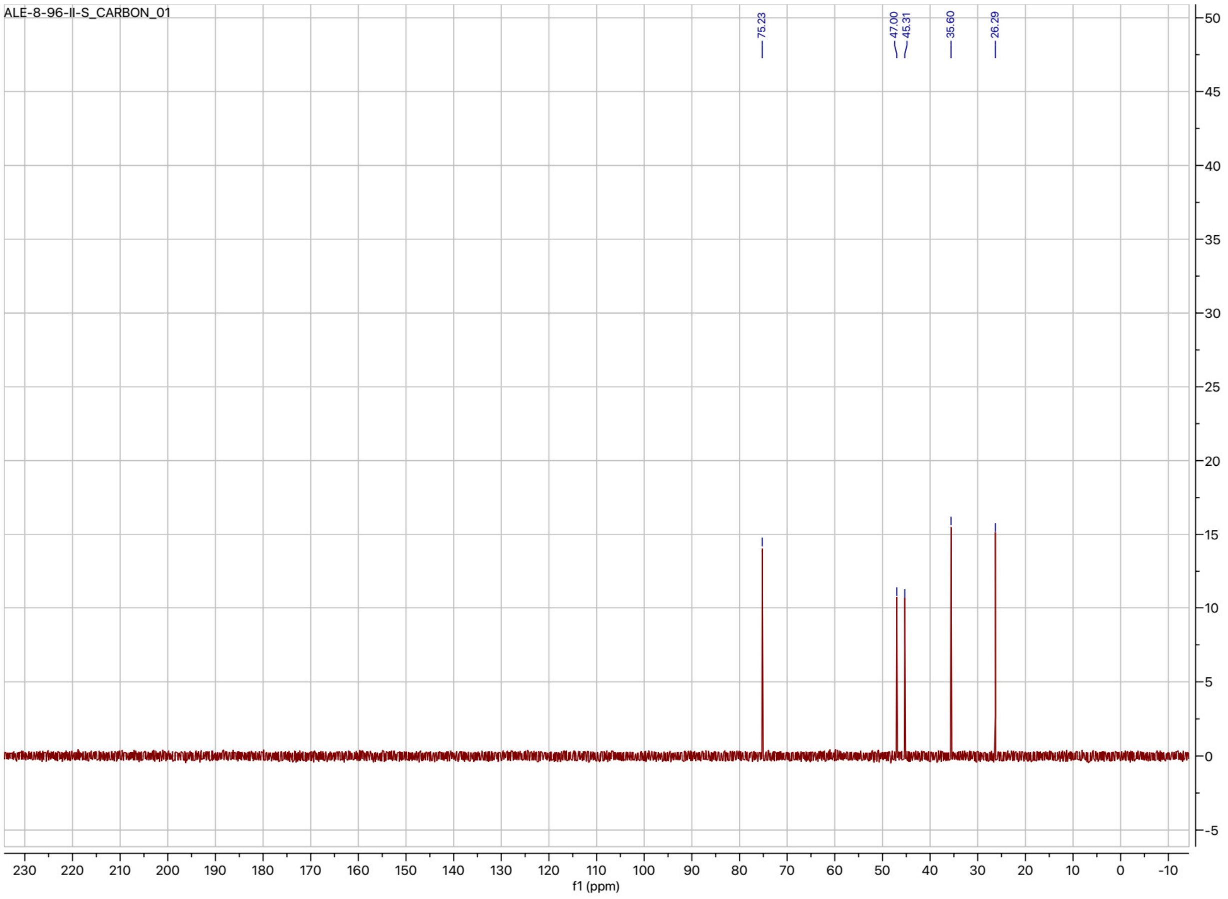

Compound 10*S*-PLP- ^{1}H NMR in D_2O

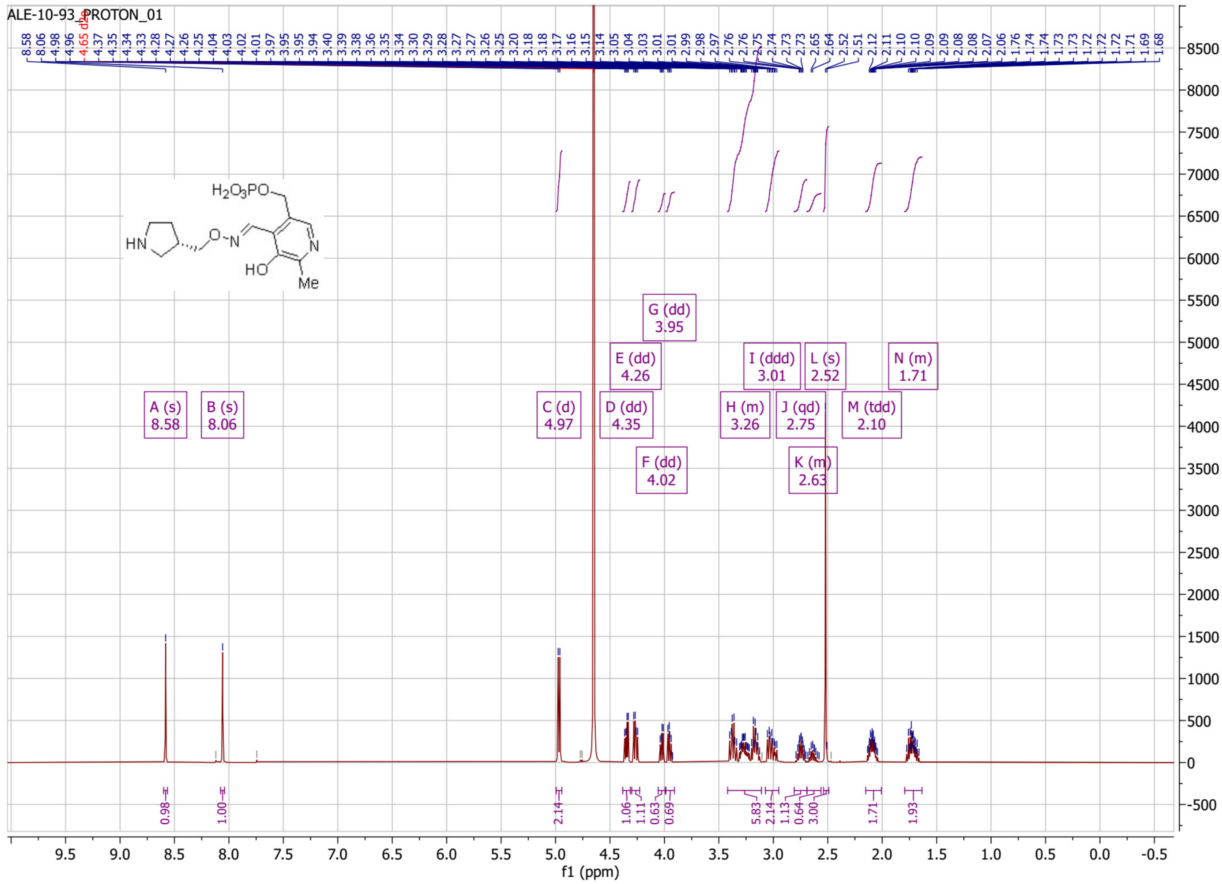

Compound 10*S*-PLP- ^{13}C NMR in D_2O

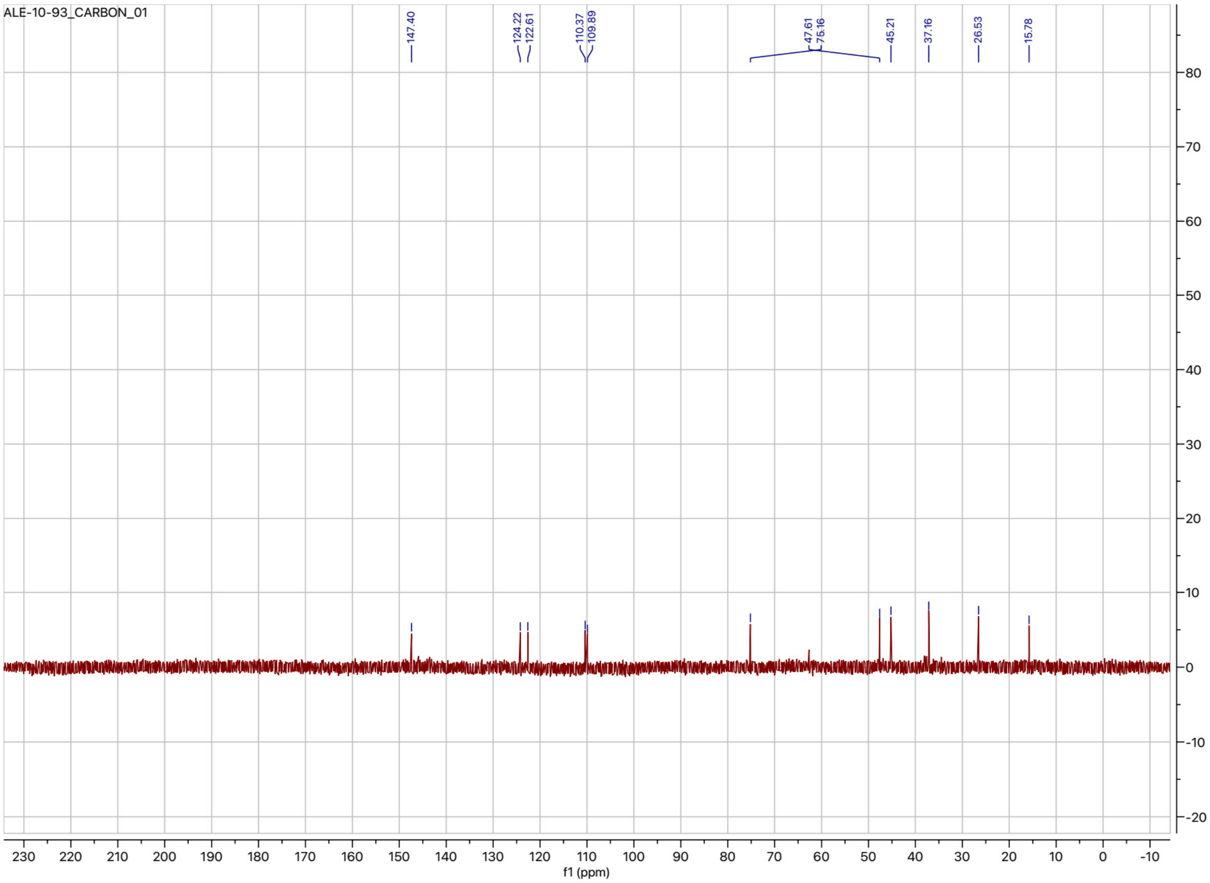

Compound 11- ^{1}H NMR in D_2O

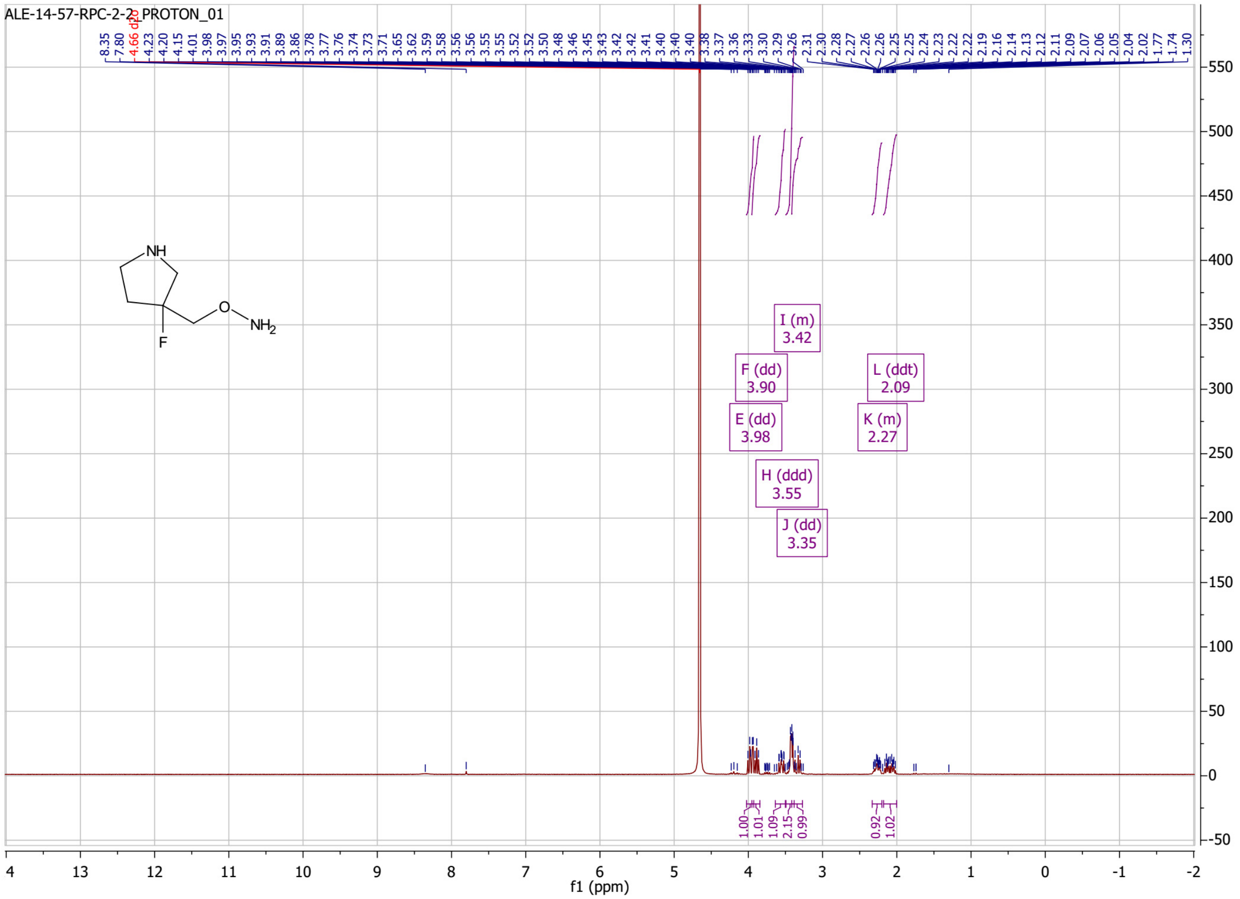

Compound 11- ^{13}C NMR in D_2O

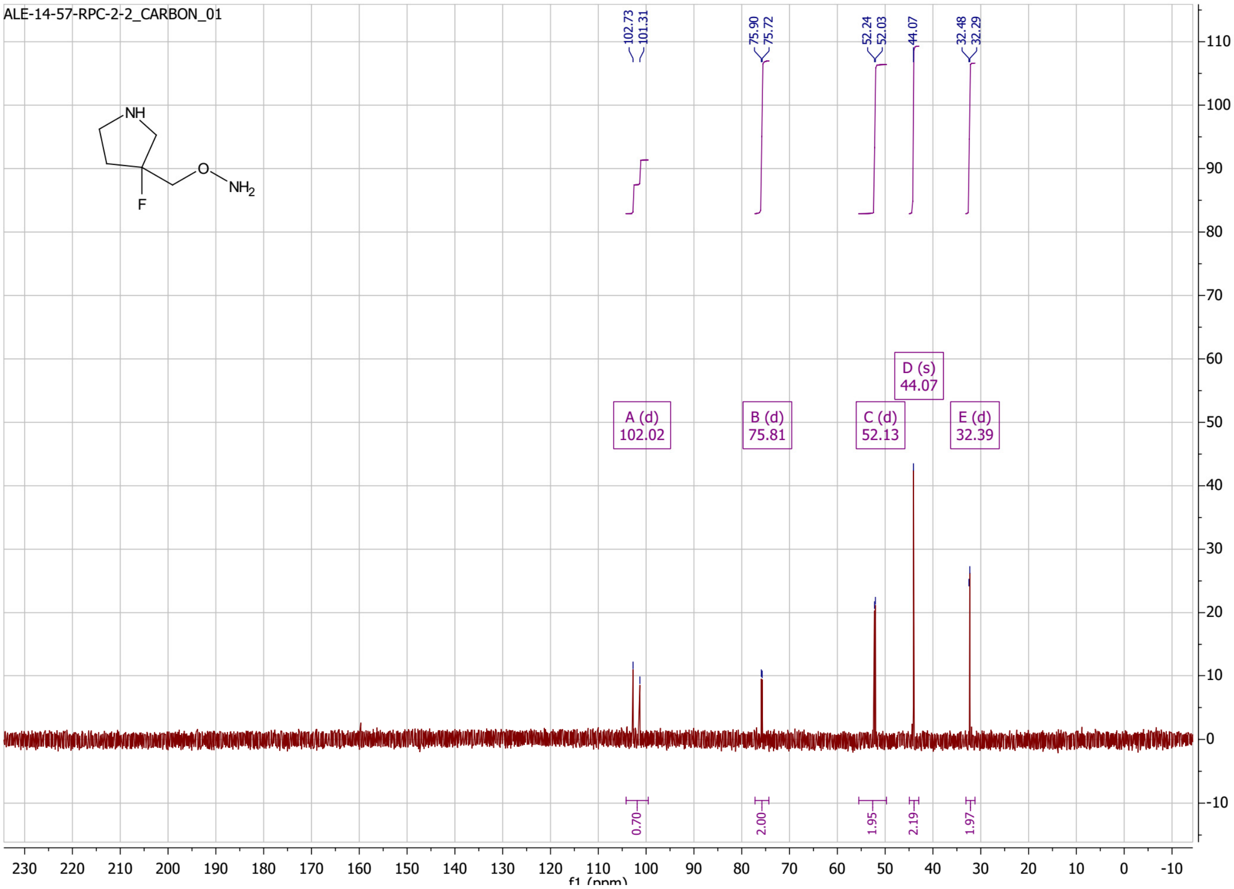

Compound 11-PLP- ^{1}H NMR in D_2O

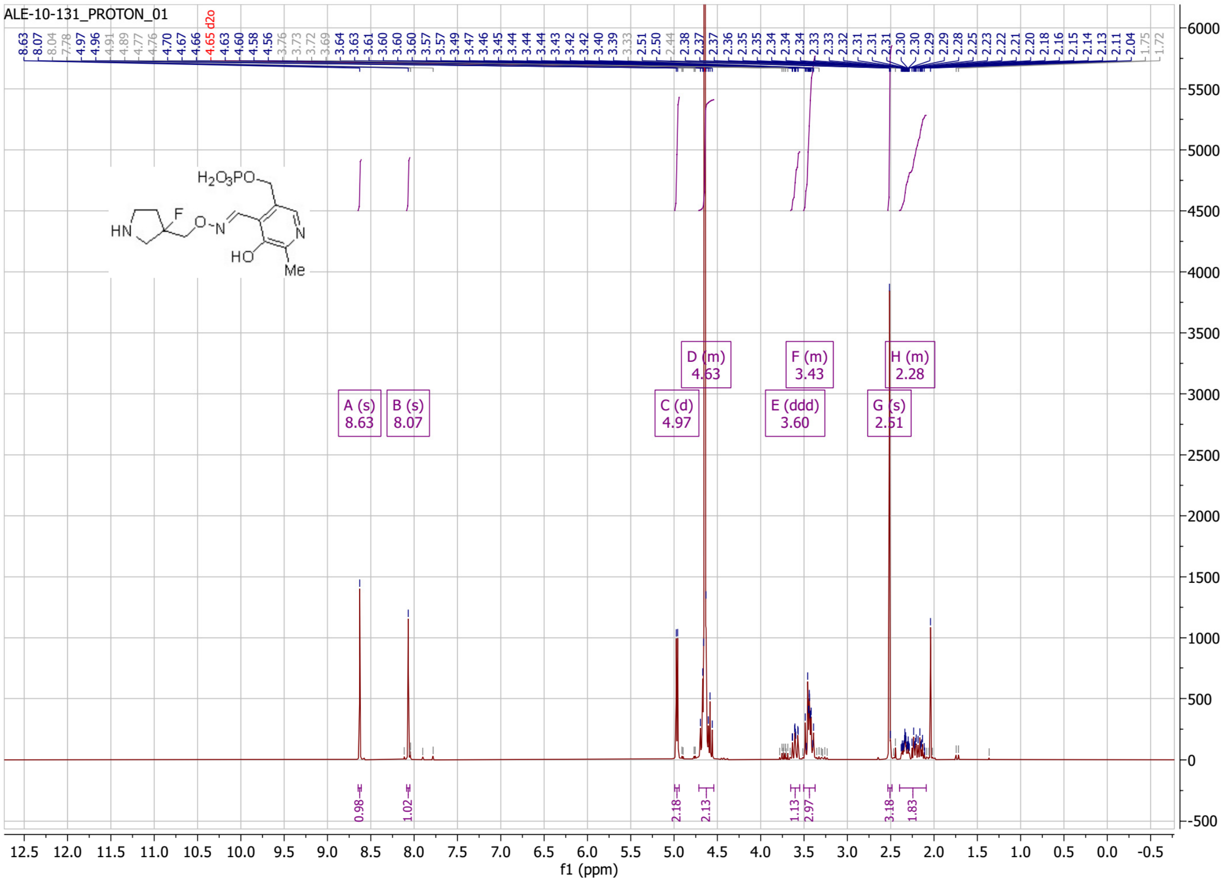

Compound 11-PLP- ^{13}C NMR in D_2O

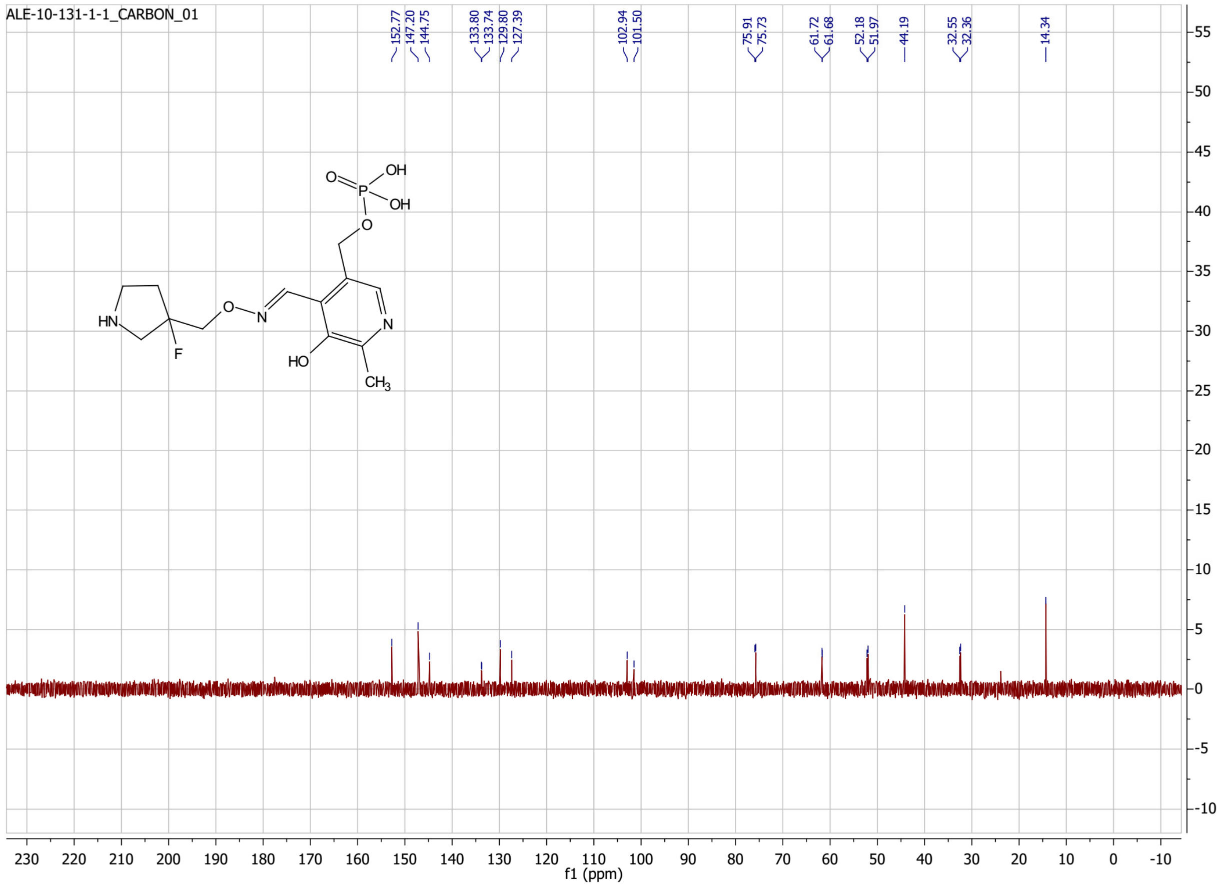

Appendix 2. HPLC chromatograms

Compound 10*R*-PLP-HPLC

```
=====================================================================
Acq. Operator   : BH
Acq. Instrument : Instrument 1                    Location :   -
Injection Date  : 12/10/2024 2:23:32 PM
Acq. Method     : C:\CHEM32\1\METHODS\BH-15ISO.M
Last changed    : 12/10/2024 2:03:17 PM by BH
Analysis Method : C:\CHEM32\1\METHODS\BH-TEST1-MCC42011-8.M
Last changed    : 9/8/2023 10:31:11 AM by BH
Sample Info     : Waters Delta Pak C18 300x3.9mm
                  1.00g/L NH4Ac 20uL
```

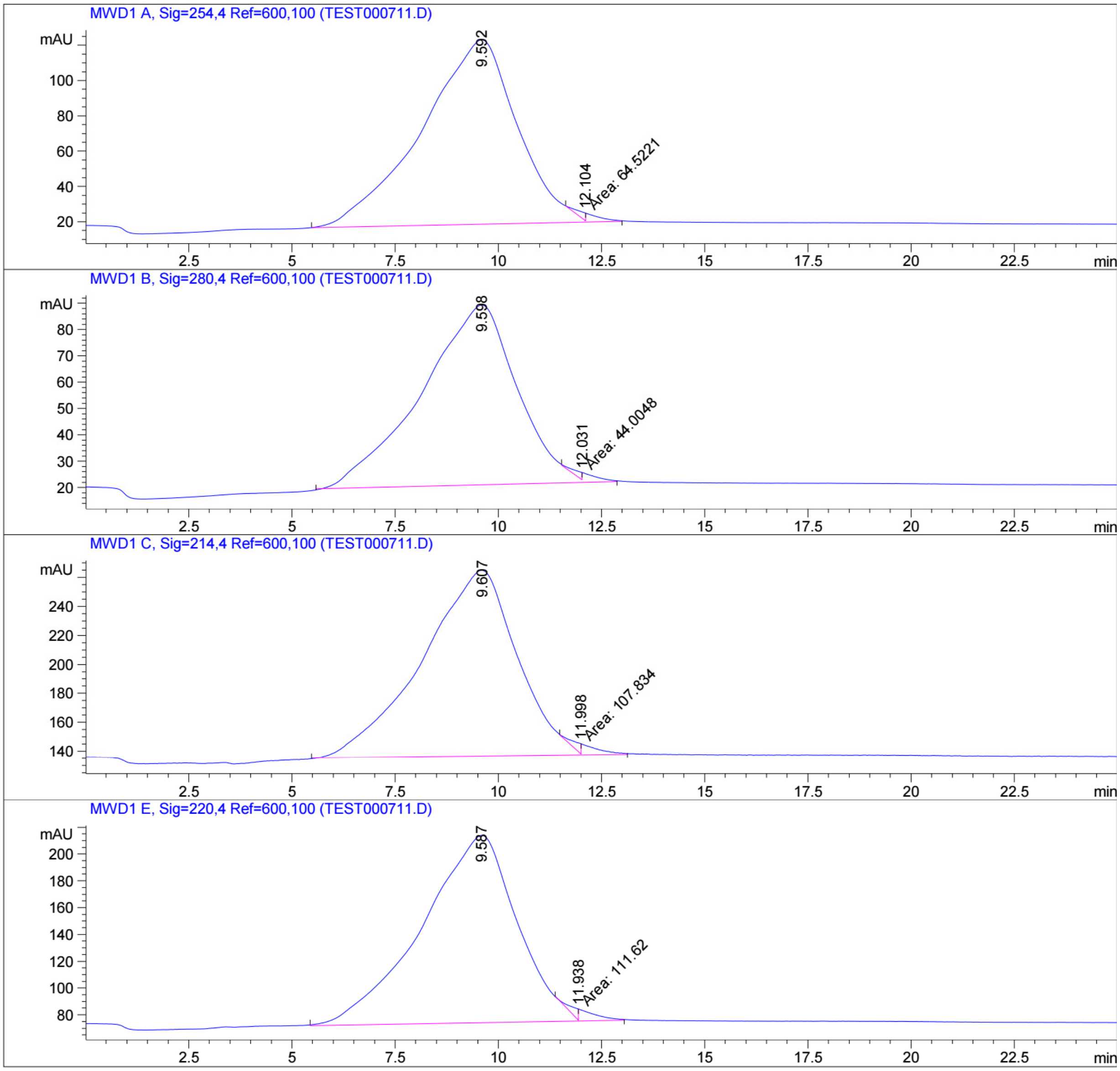

trument 1 12/11/2024 1:16:00 PM BH   Page 1 of 3

Signal 1: MWD1 A, Sig=254,4 Ref=600,100

Peak #	RetTime [min]	Type	Width [min]	Area [mAU*s]	Height [mAU]	Area %
1	9.592	MM R	2.6997	1.69386e4	104.57059	99.6205
2	12.104	MM T	0.2408	64.52214	4.46556	0.3795

Totals : 1.70031e4 109.03615

Signal 2: MWD1 B, Sig=280,4 Ref=600,100

Peak #	RetTime [min]	Type	Width [min]	Area [mAU*s]	Height [mAU]	Area %
1	9.598	MM R	2.7055	1.11020e4	68.39097	99.6052
2	12.031	MM T	0.2568	44.00480	2.85643	0.3948

Totals : 1.11460e4 71.24739

Signal 3: MWD1 C, Sig=214,4 Ref=600,100

Peak #	RetTime [min]	Type	Width [min]	Area [mAU*s]	Height [mAU]	Area %
1	9.607	MM R	2.6960	2.07963e4	128.56328	99.4841
2	11.998	MM T	0.2465	107.83448	7.29121	0.5159

Totals : 2.09041e4 135.85449

Signal 4: MWD1 E, Sig=220,4 Ref=600,100

Peak #	RetTime [min]	Type	Width [min]	Area [mAU*s]	Height [mAU]	Area %
1	9.587	MM R	2.6964	2.26524e4	140.01642	99.5097
2	11.938	MM T	0.2163	111.62032	8.59892	0.4903

Totals : 2.27640e4 148.61533

Compound 10*S*-PLP-HPLC

```
=====================================================================
Acq. Operator   : BH
Acq. Instrument : Instrument 1                    Location :   -
Injection Date  : 12/10/2024 3:50:42 PM
Acq. Method     : C:\CHEM32\1\METHODS\BH-15ISO.M
Last changed    : 12/10/2024 3:44:57 PM by BH
Analysis Method : C:\CHEM32\1\METHODS\BH-TEST1-MCC42011-8.M
Last changed    : 9/8/2023 10:31:11 AM by BH
Sample Info     : Waters Delta Pak C18 300x3.9mm
                  1.00g/L NH4Ac 20uL
```

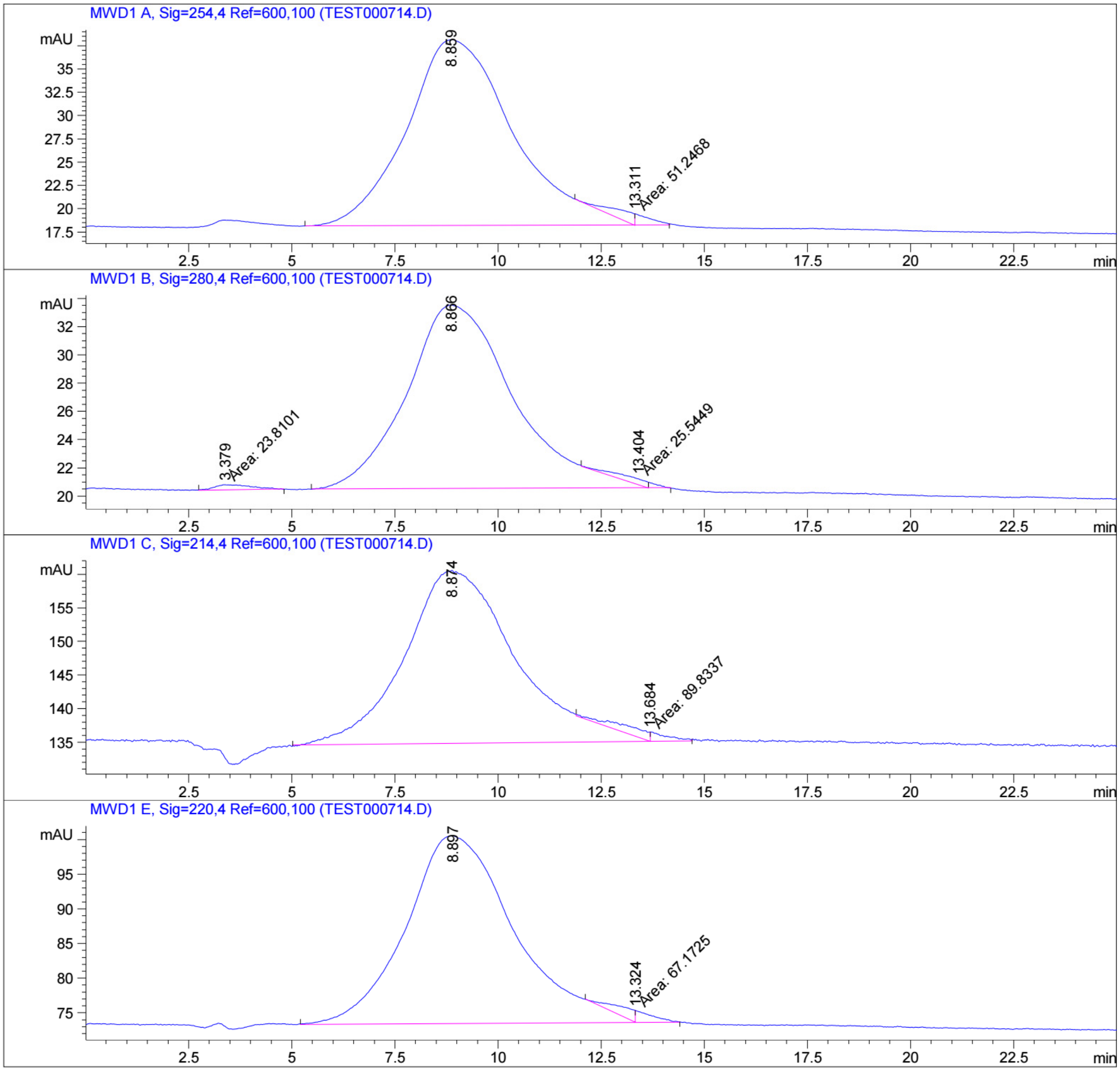

Signal 1: MWD1 A, Sig=254,4 Ref=600,100

Peak #	RetTime [min]	Type	Width [min]	Area [mAU*s]	Height [mAU]	Area %
1	8.859	MM R	3.0203	3618.55005	19.96766	98.6036
2	13.311	MM T	0.6826	51.24675	1.25127	1.3964
Totals :				3669.79680	21.21893	

Signal 2: MWD1 B, Sig=280,4 Ref=600,100

Peak #	RetTime [min]	Type	Width [min]	Area [mAU*s]	Height [mAU]	Area %
1	3.379	MM	1.0612	23.81009	3.73932e-1	0.9953
2	8.866	MM R	3.0044	2343.00562	12.99771	97.9370
3	13.404	MM T	1.1172	25.54486	4.27481e-1	1.0678
Totals :				2392.36056	13.79913	

Signal 3: MWD1 C, Sig=214,4 Ref=600,100

Peak #	RetTime [min]	Type	Width [min]	Area [mAU*s]	Height [mAU]	Area %
1	8.874	MM R	3.1436	4860.49902	25.76891	98.1853
2	13.684	MM T	1.0621	89.83371	1.40970	1.8147
Totals :				4950.33273	27.17862	

Signal 4: MWD1 E, Sig=220,4 Ref=600,100

Peak #	RetTime [min]	Type	Width [min]	Area [mAU*s]	Height [mAU]	Area %
1	8.897	MM R	3.0652	4989.15234	27.12831	98.6715
2	13.324	MM T	0.6465	67.17246	1.73161	1.3285

Totals : 5056.32481 28.85992

Compound 11-PLP-HPLC

```
=====================================================================
Acq. Operator   : BH
Acq. Instrument : Instrument 1                   Location :   -
Injection Date  : 12/10/2024 5:56:45 PM
Acq. Method     : C:\CHEM32\1\METHODS\BH-15ISO.M
Last changed    : 12/10/2024 5:42:54 PM by BH
Analysis Method : C:\CHEM32\1\METHODS\BH-TEST1-MCC42011-8.M
Last changed    : 9/8/2023 10:31:11 AM by BH
Sample Info     : Waters Delta Pak C18 300x3.9mm
                  1.00g/L NH4Ac 20uL
```

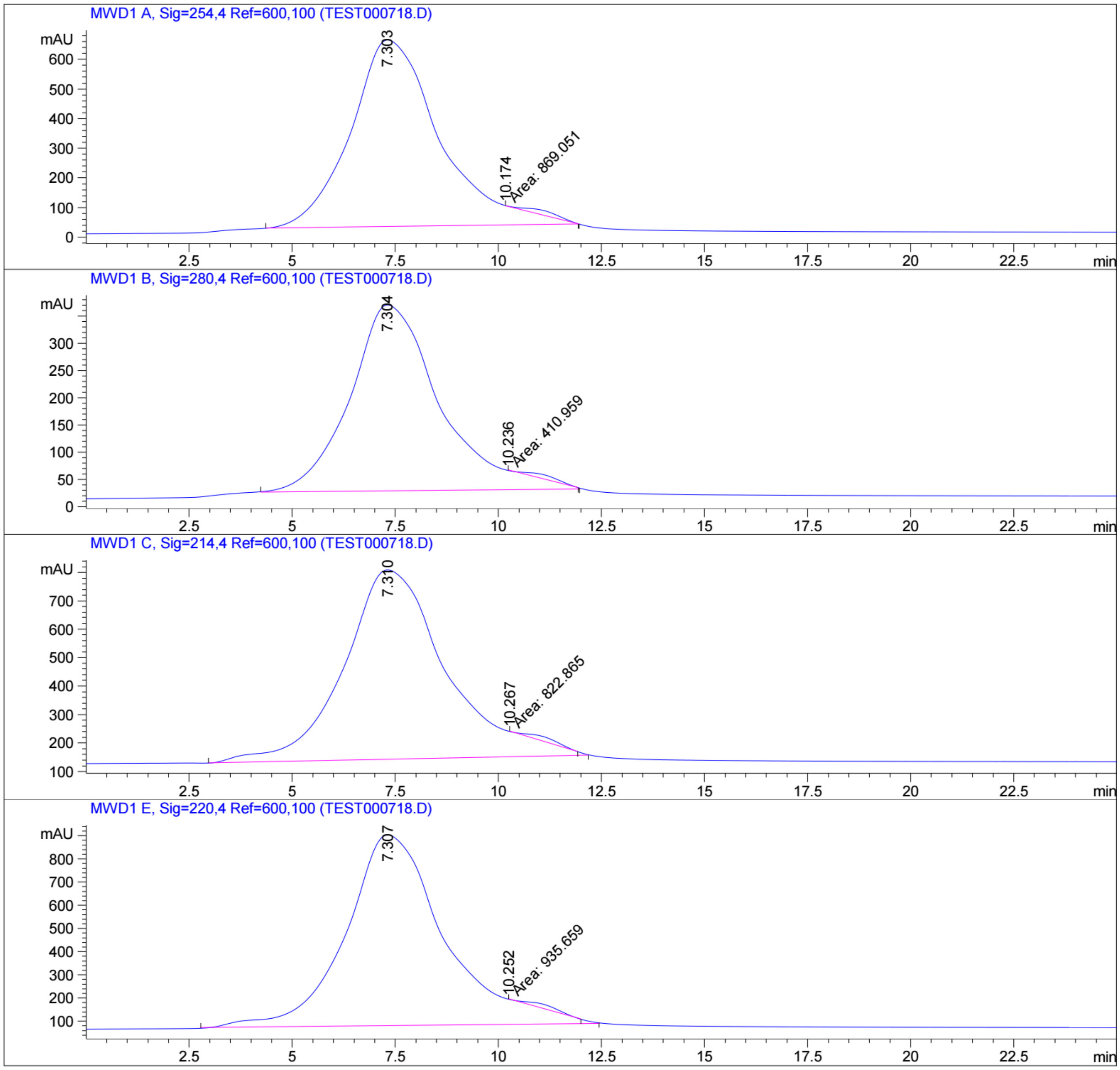

Signal 1: MWD1 A, Sig=254,4 Ref=600,100

Peak #	RetTime [min]	Type	Width [min]	Area [mAU*s]	Height [mAU]	Area %
1	7.303	MM R	2.5131	9.51328e4	630.92059	99.0948
2	10.174	MM T	13.1153	869.05060	7.73806e-1	0.9052

Totals : 9.60018e4 631.69440

Signal 2: MWD1 B, Sig=280,4 Ref=600,100

Peak #	RetTime [min]	Type	Width [min]	Area [mAU*s]	Height [mAU]	Area %
1	7.304	MM R	2.5328	5.19848e4	342.07965	99.2157
2	10.236	MM T	0.0000	410.95856	3.91687e-1	0.7843

Totals : 5.23957e4 342.47134

Signal 3: MWD1 C, Sig=214,4 Ref=600,100

Peak #	RetTime [min]	Type	Width [min]	Area [mAU*s]	Height [mAU]	Area %
1	7.310	MM R	2.8830	1.15351e5	666.84521	99.2917
2	10.267	MM T	0.0000	822.86517	1.04274	0.7083

Totals : 1.16174e5 667.88796

Compound APA-PLP-HPLC (E:Z ratio 96:4 by ^{1}H NMR)

```
=====================================================================
Acq. Operator   : BH
Acq. Instrument : Instrument 1                    Location :   -
Injection Date  : 12/10/2024 6:59:30 PM
Acq. Method     : C:\CHEM32\1\METHODS\BH-15ISO.M
Last changed    : 12/10/2024 6:53:48 PM by BH
Analysis Method : C:\CHEM32\1\METHODS\BH-TEST1-MCC42011-8.M
Last changed    : 9/8/2023 10:31:11 AM by BH
Sample Info     : Waters Delta Pak C18 300x3.9mm
                  1.00g/L NH4Ac 20uL
```

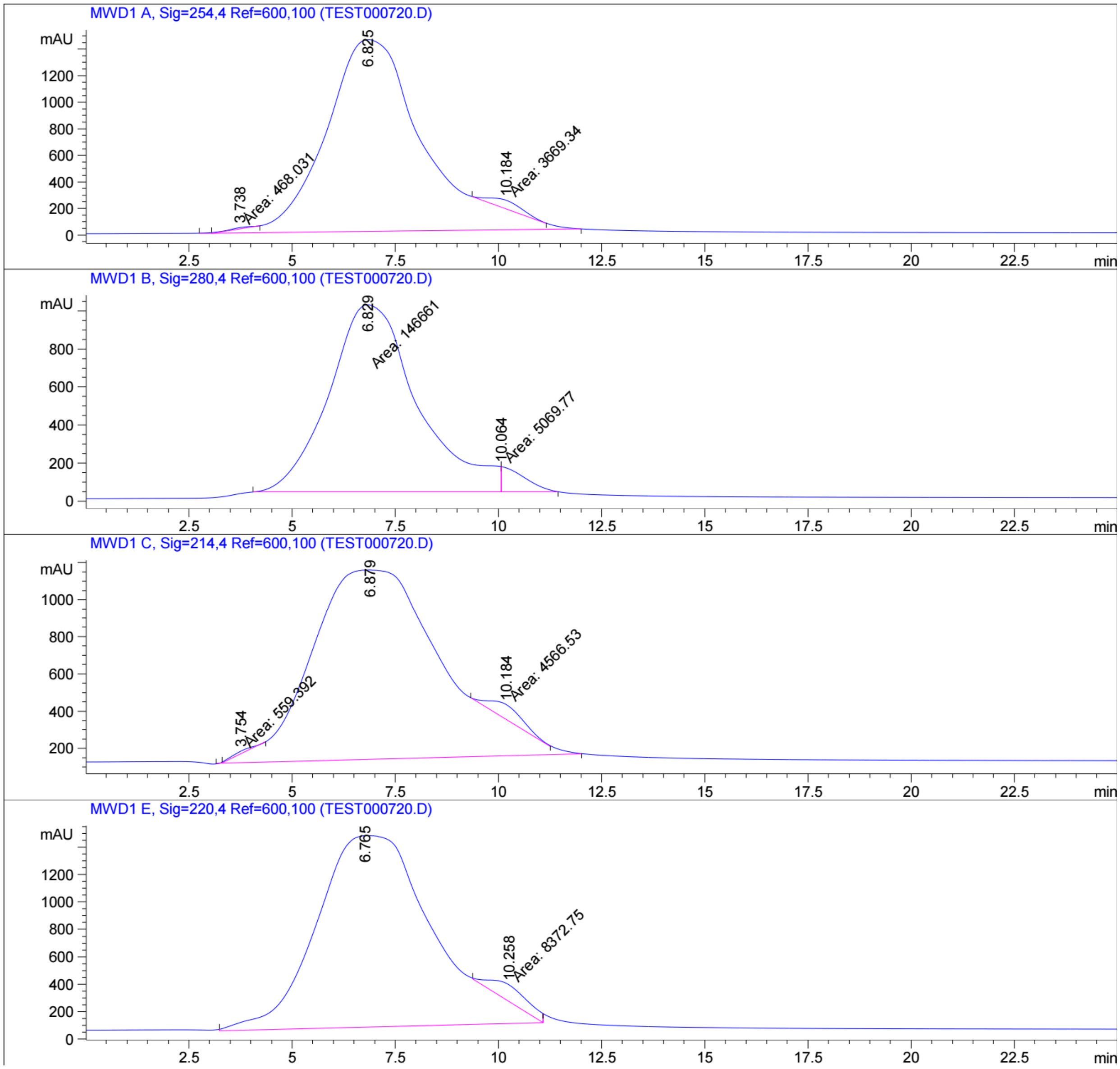

Signal 1: MWD1 A, Sig=254,4 Ref=600,100

Peak #	RetTime [min]	Type	Width [min]	Area [mAU*s]	Height [mAU]	Area %
1	3.738	MM T	0.7547	468.03070	11.48117	0.1917
2	6.825	MM R	2.7660	2.40003e5	1446.12463	98.3053
3	10.184	MM T	0.9314	3669.33740	65.66273	1.5030

Totals : 2.44140e5 1523.26854

Signal 2: MWD1 B, Sig=280,4 Ref=600,100

Peak #	RetTime [min]	Type	Width [min]	Area [mAU*s]	Height [mAU]	Area %
1	6.829	MF	2.4873	1.46661e5	982.72107	96.6587
2	10.064	FM	0.6389	5069.77197	132.25409	3.3413

Totals : 1.51730e5 1114.97516

Signal 3: MWD1 C, Sig=214,4 Ref=600,100

Peak #	RetTime [min]	Type	Width [min]	Area [mAU*s]	Height [mAU]	Area %
1	3.754	MM T	0.6008	559.39197	15.51724	0.2419
2	6.879	MM R	3.6927	2.26126e5	1020.60229	97.7834
3	10.184	MM T	0.9519	4566.52734	79.95071	1.9747

Totals : 2.31252e5 1116.07025

Appendix 3. HRMS spectra

Compound APA-PLP_HRMS_ESI+

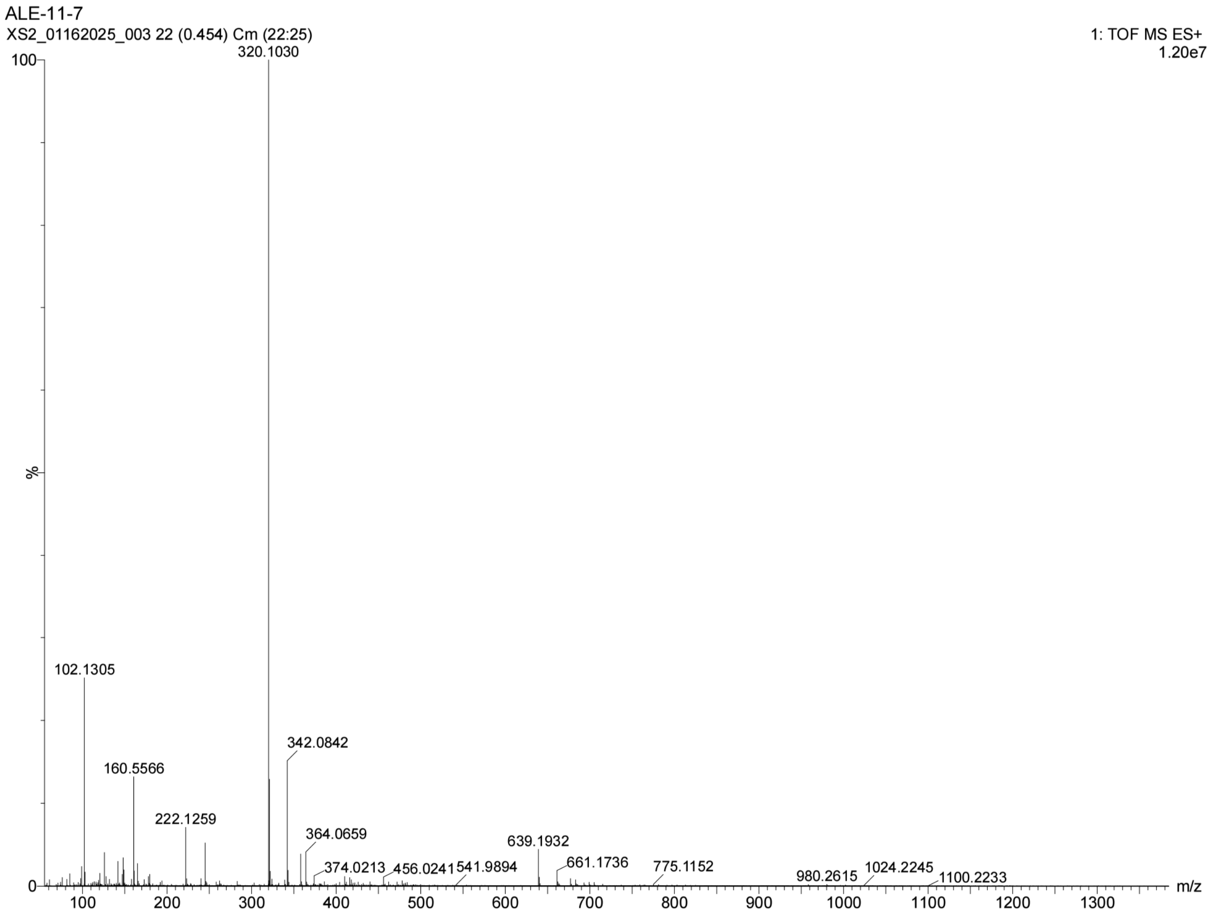

Compound 5_HRMS_ESI+

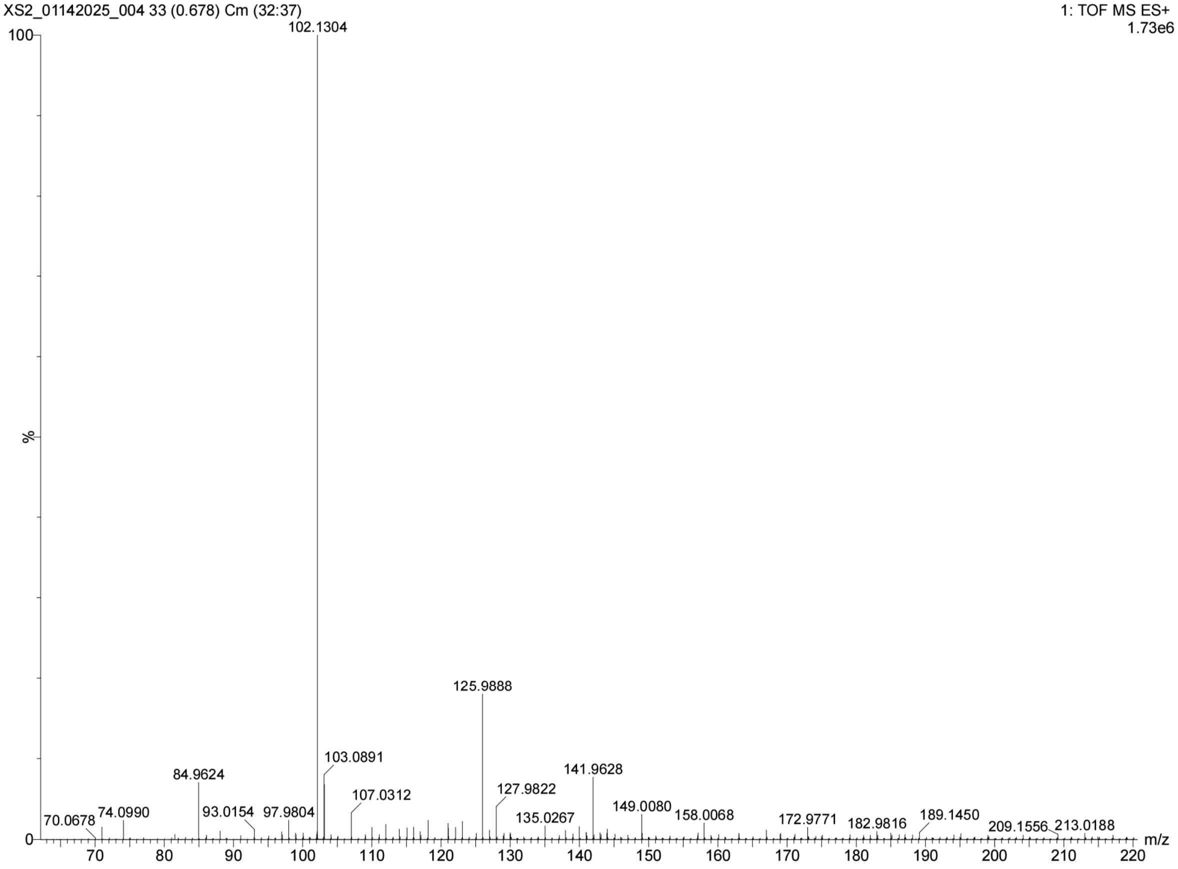

Compound 6_HRMS_ESI+

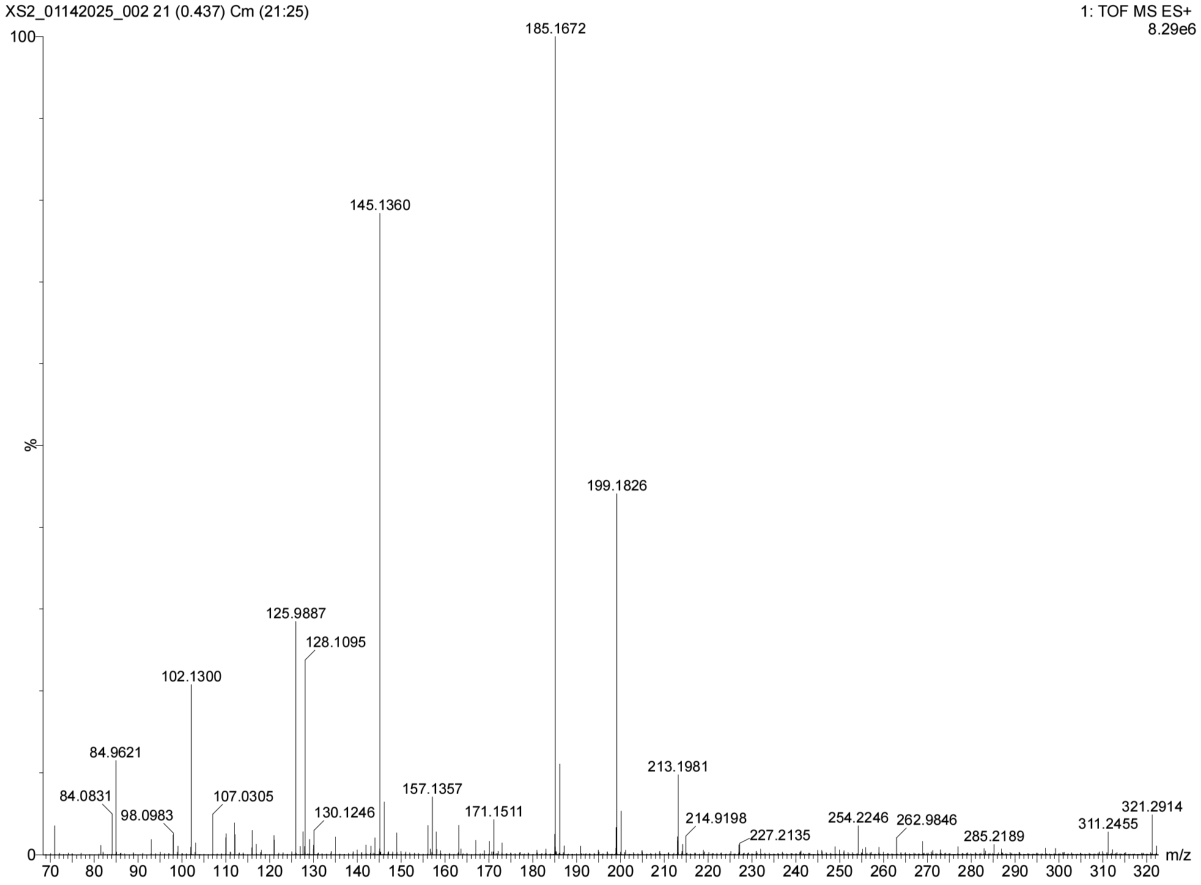

Compound 7_HRMS_ESI+

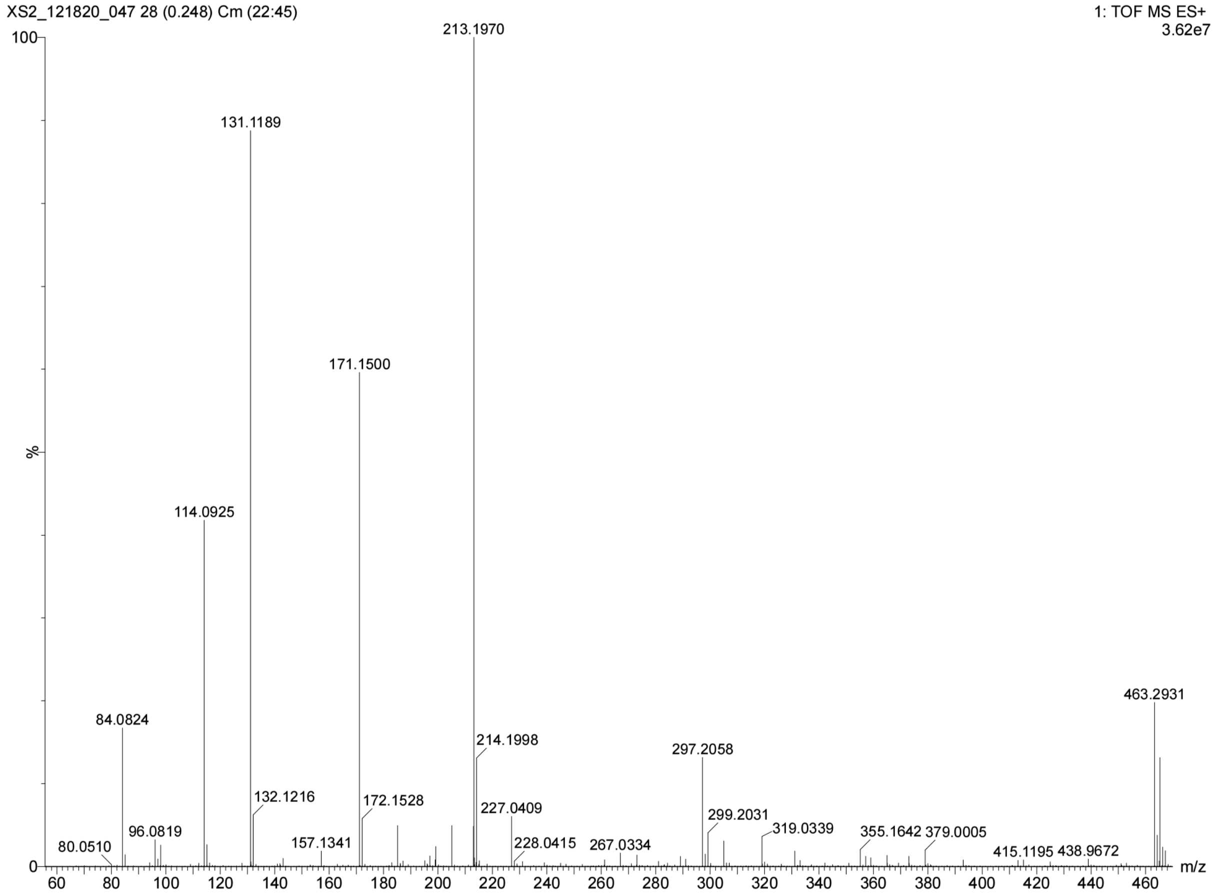

Compound 7-*R*_HRMS_ESI+

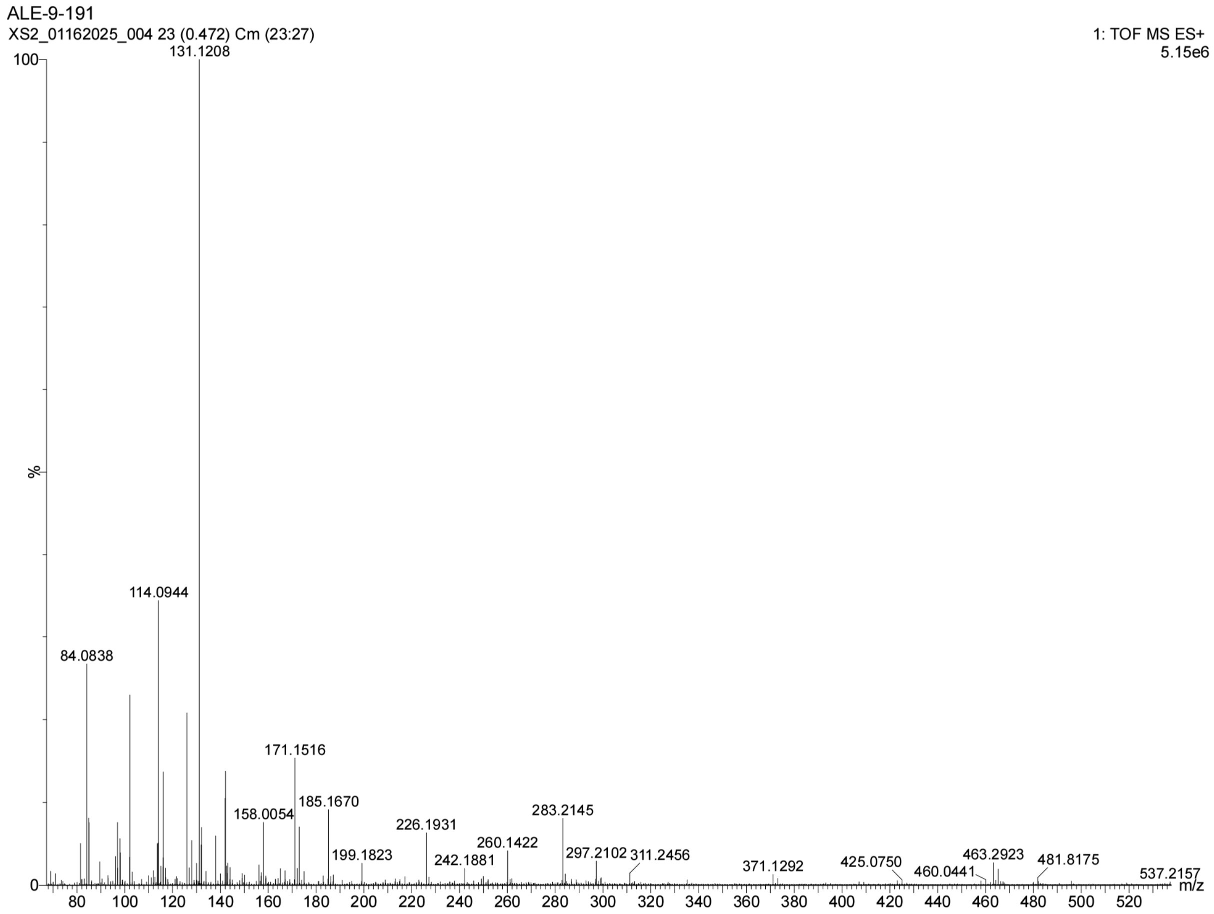

Compound 7-*S*_HRMS_ESI+

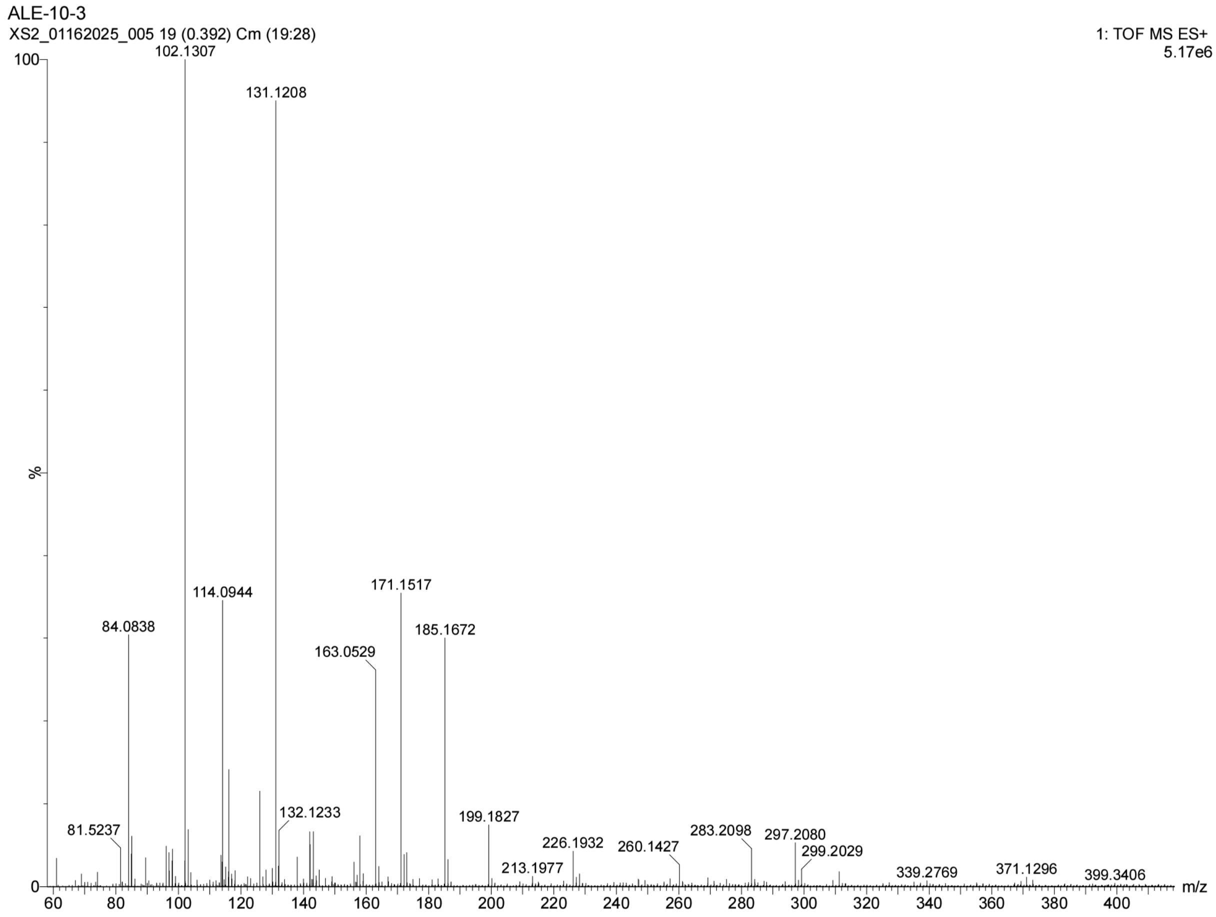

Compound 8_HRMS_ESI+

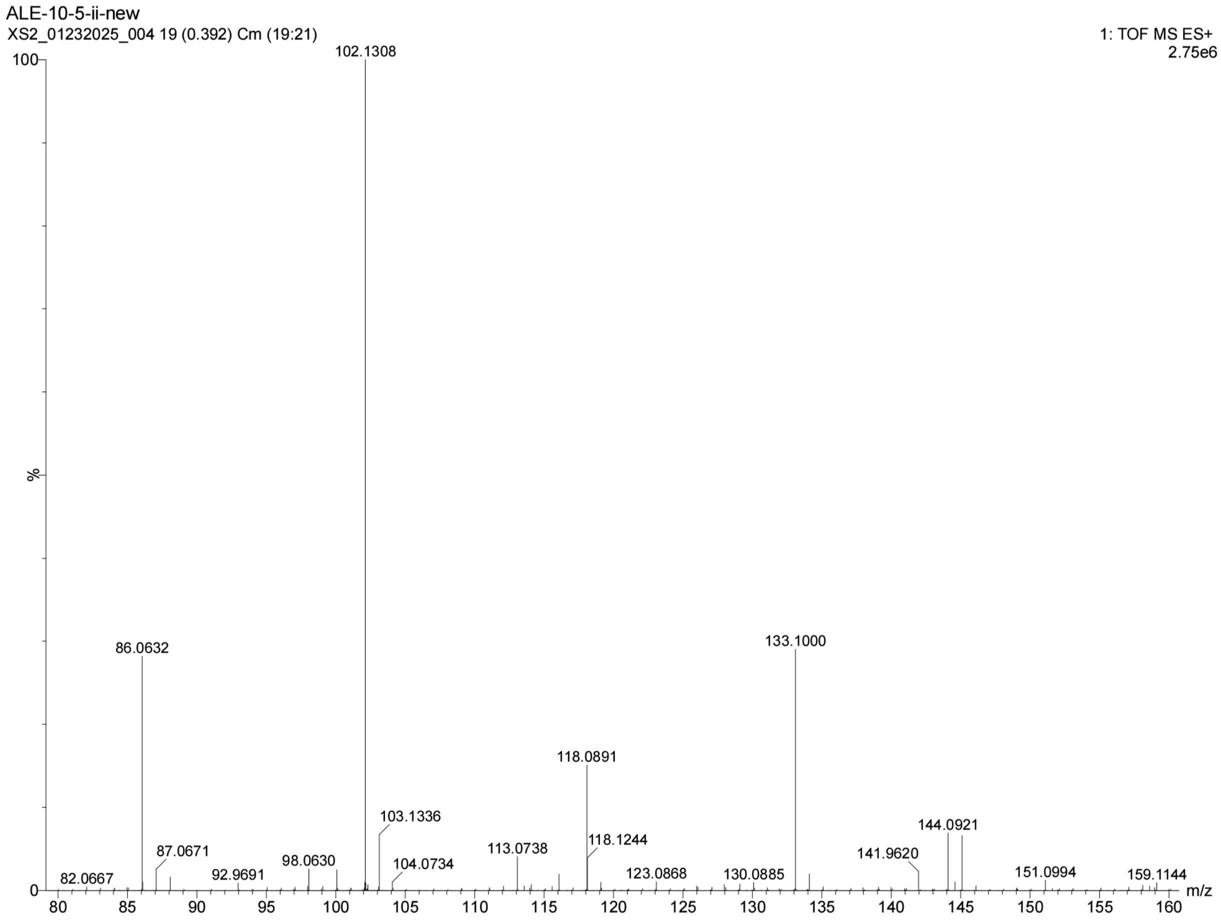

Compound 9_HRMS_ESI+

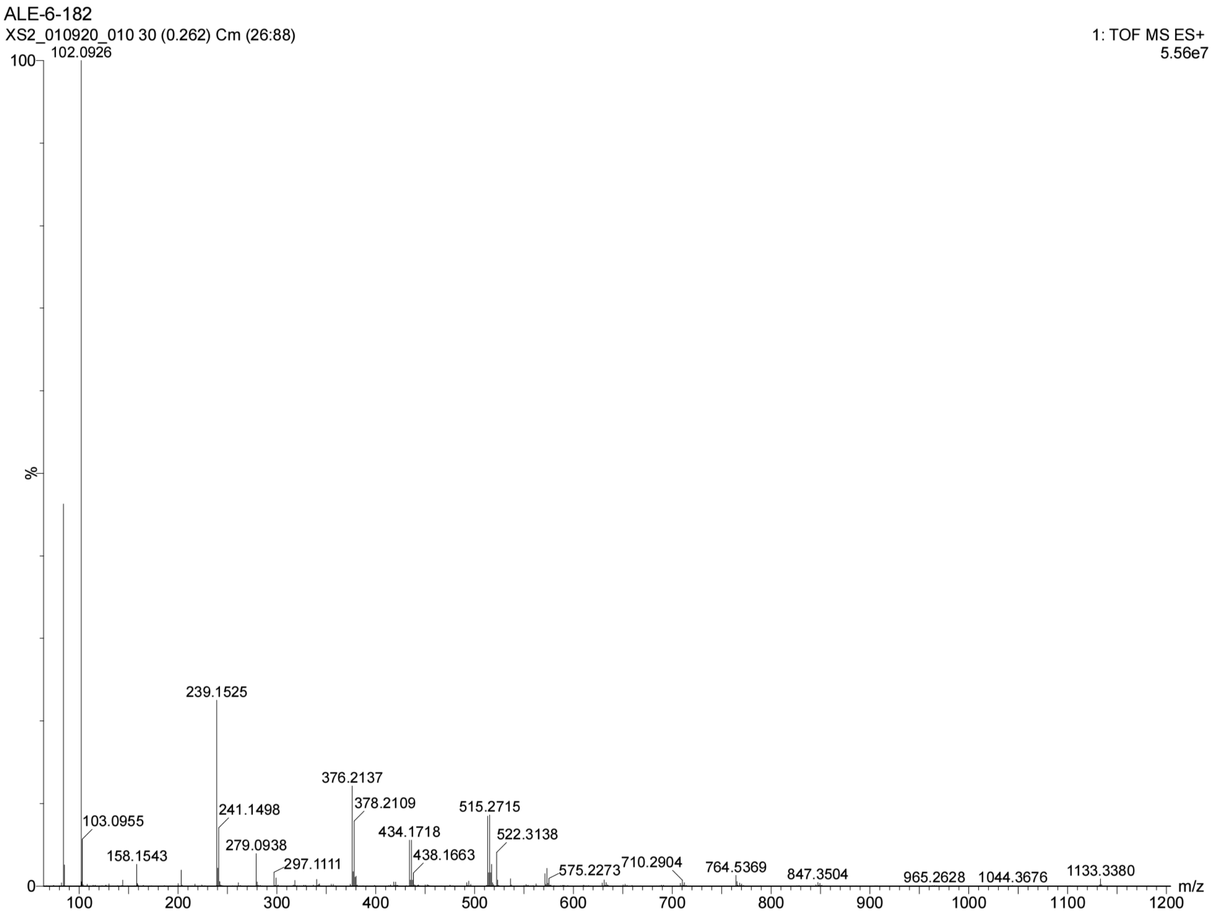

Compound 10_HRMS_ESI+

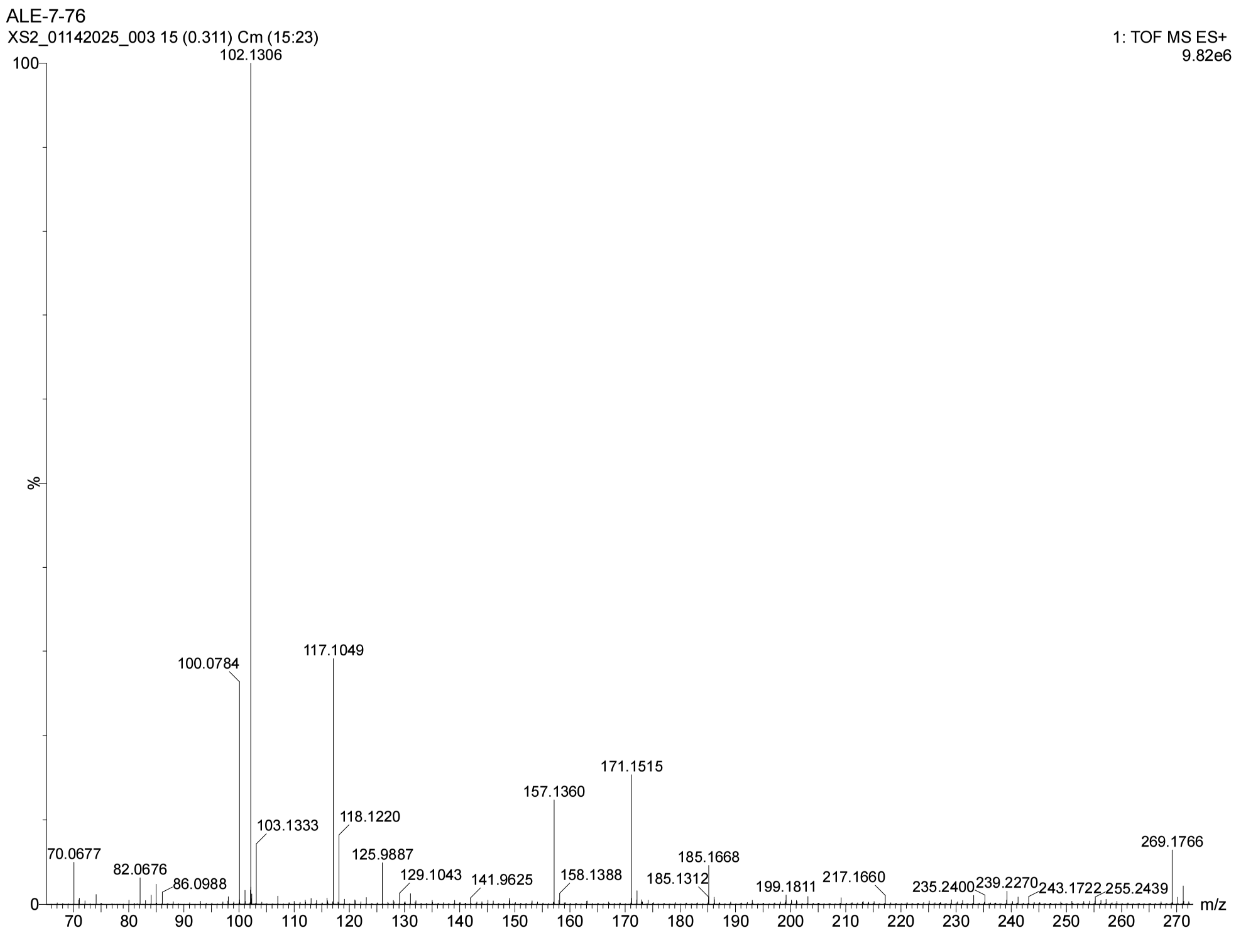

Compound 10-*R*_HRMS_ESI+

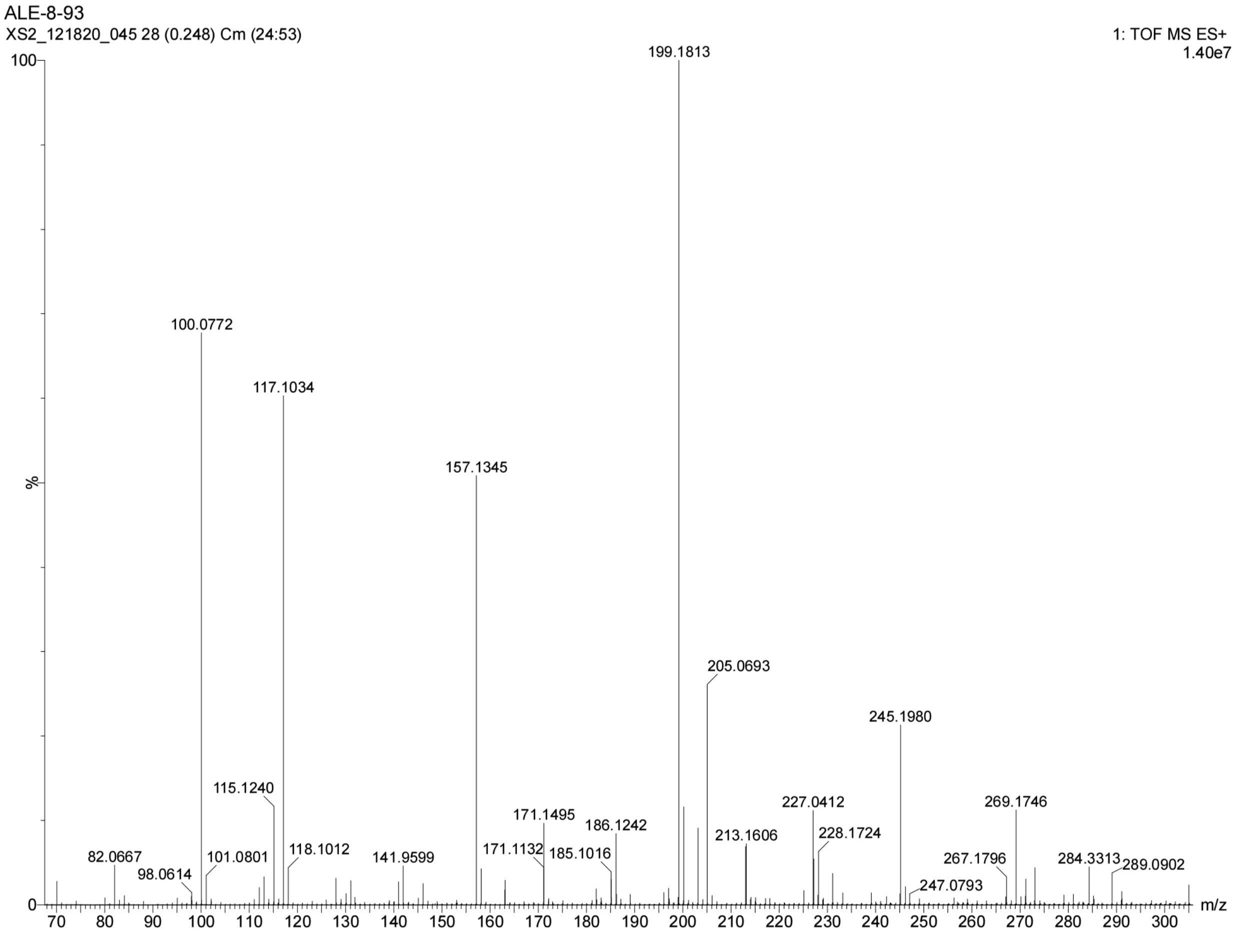

Compound 10-*S*_HRMS_ESI+

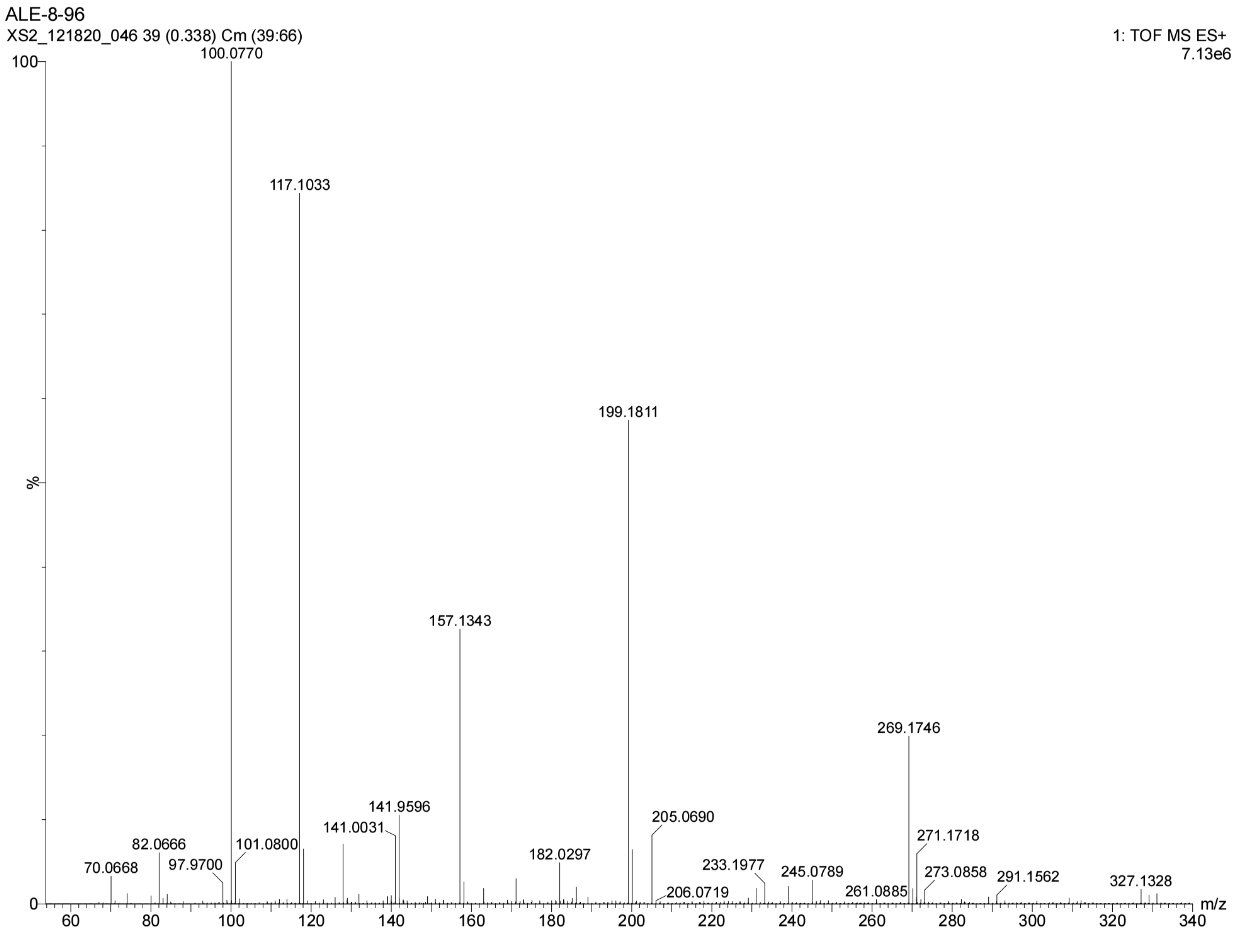

Compound 10-*S*-PLP_HRMS_ESI+

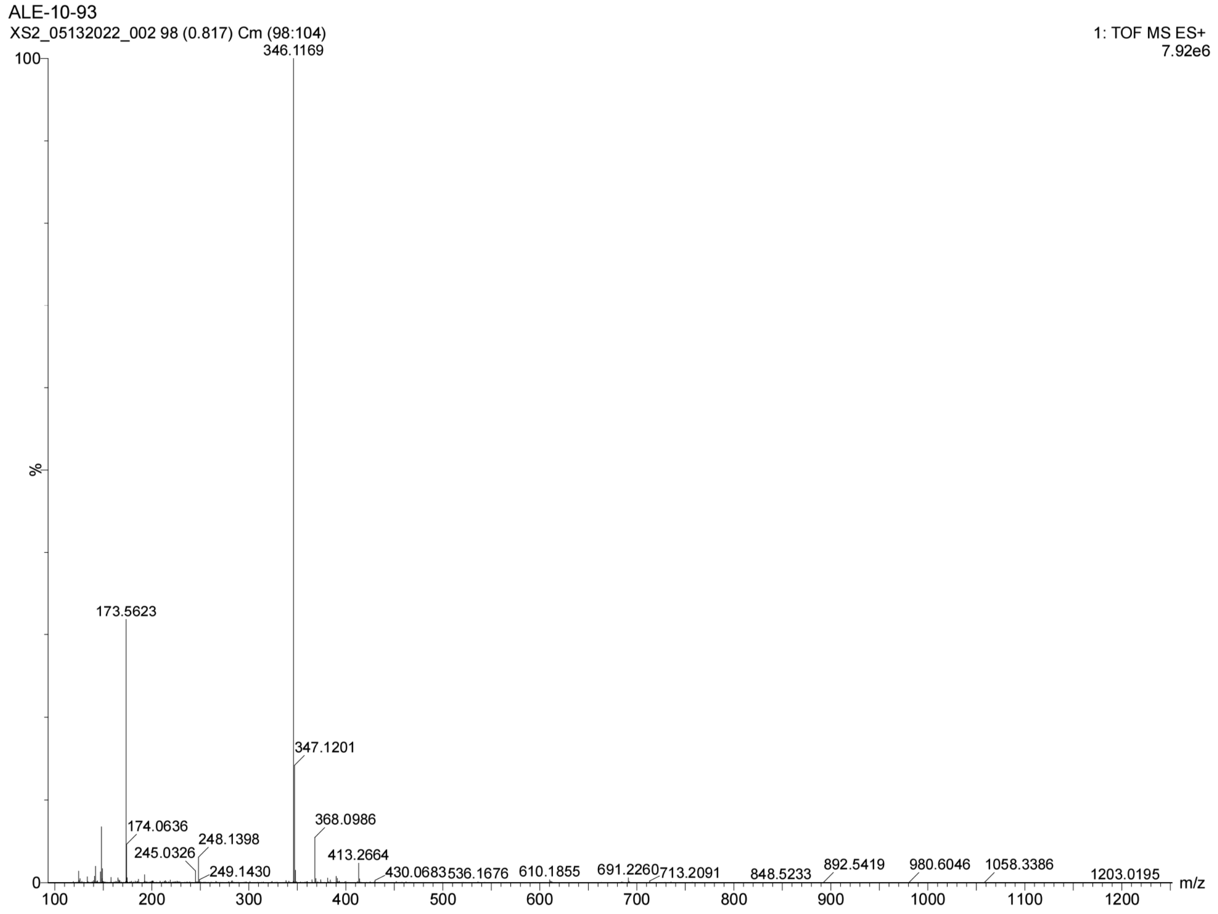

Compound 10-*R*-PLP_HRMS_ESI+

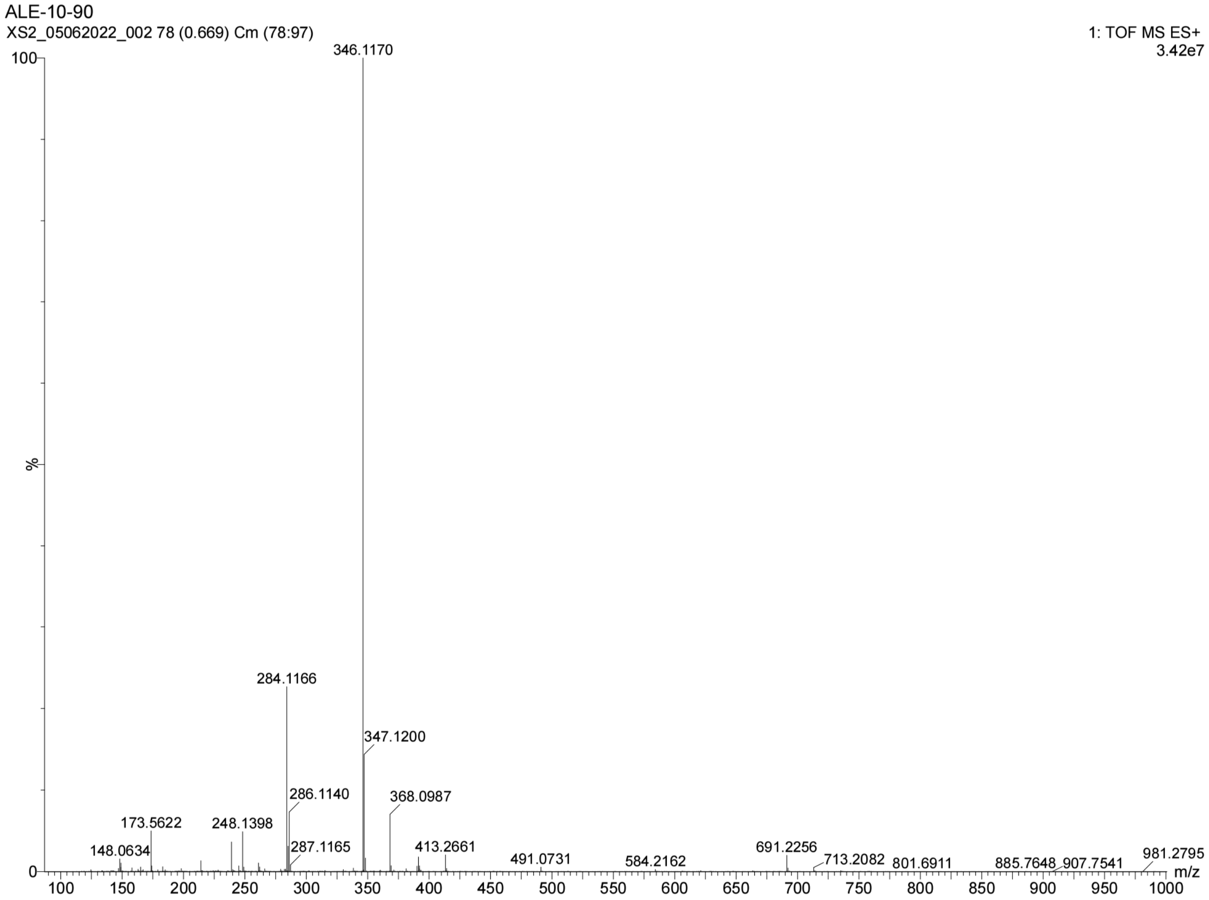

Compound 11_HRMS_ESI+

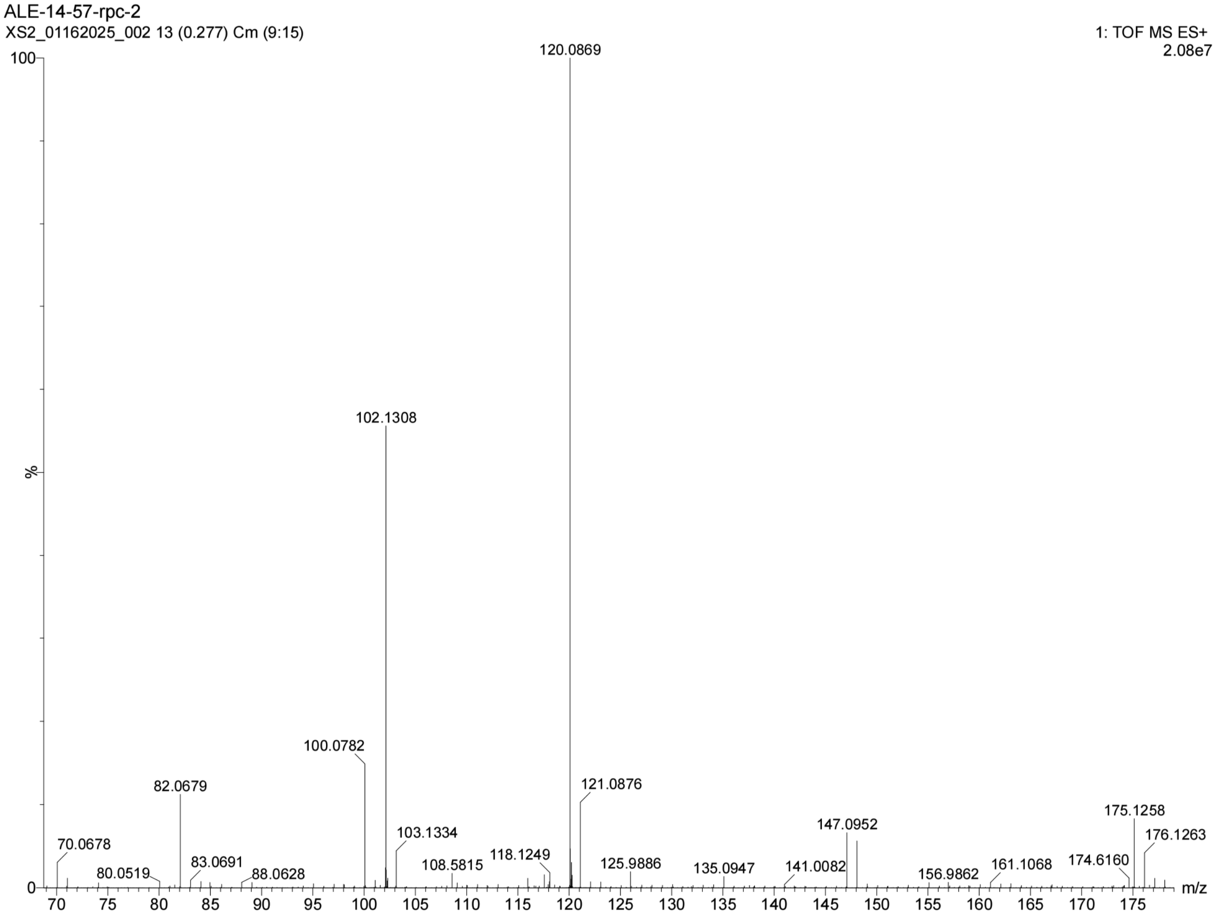

Compound 11-PLP_HRMS_ESI+

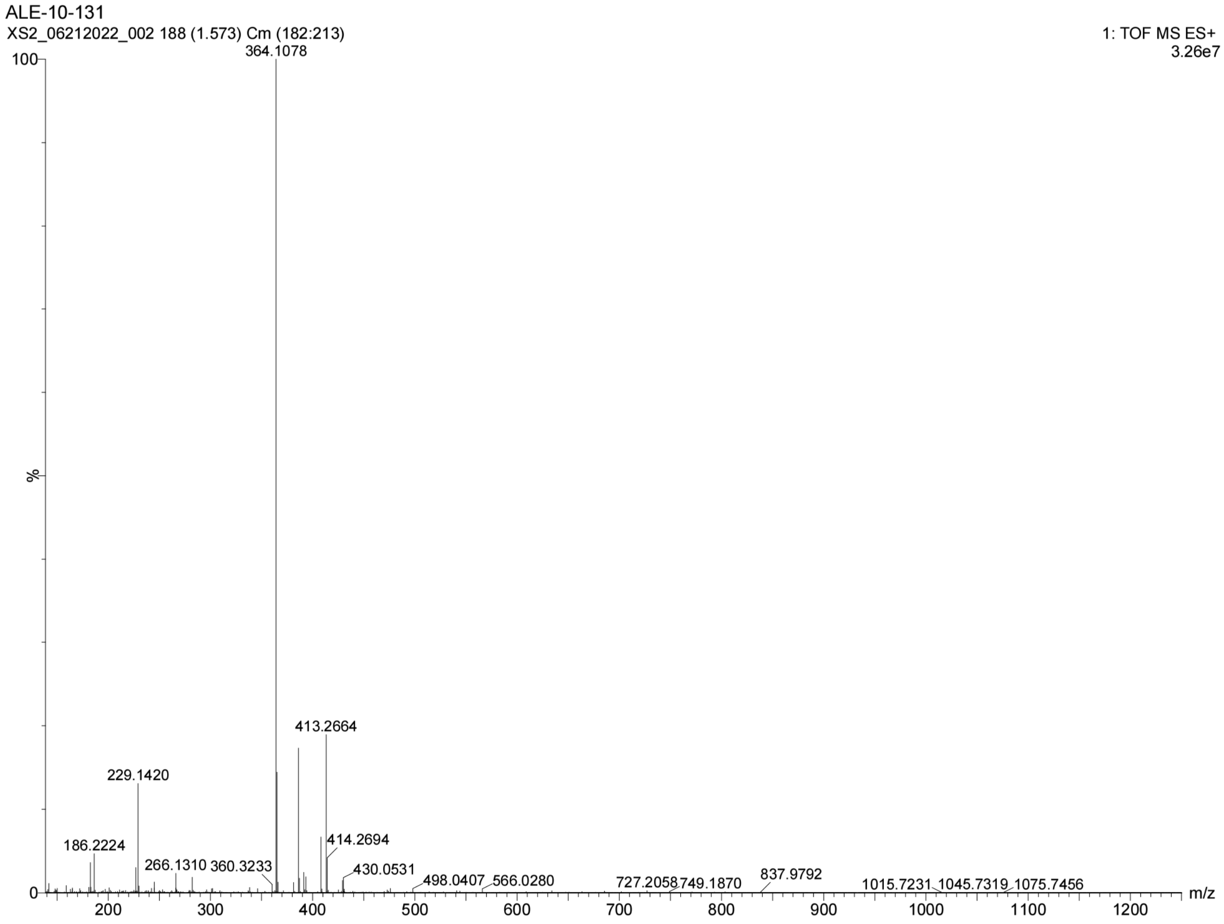

Supplement: Supplementary file 1 — jm4c03120_si_001.pdf [file jm4c03120_si_001.pdf]
